# Access and Modulation of Substituted Pyrrolo[3,4-*c*]pyrazole-4,6-(2*H*,5*H*)-diones

**DOI:** 10.3390/molecules28155811

**Published:** 2023-08-01

**Authors:** Abdelaziz Ejjoummany, Jonathan Elie, Ahmed El Hakmaoui, Mohamed Akssira, Sylvain Routier, Frédéric Buron

**Affiliations:** 1 Institut de Chimie Organique et Analytique, Université d’Orléans, UMR CNRS 7311, BP 6759, CEDEX 2, F-45067 Orléans, France; 2 Faculté des Sciences et Technique, Université Hassan II-Casablanca, BP 146, Mohammedia 28800, Morocco

**Keywords:** pyrrolo[3,4-*c*] pyrazole, cross-coupling, fused [5,5] ring systems

## Abstract

The first access to polyfunctionnalized pyrrolo[3,4-*c*]pyrazole-4,6-(2*H*,5*H*)-dione derivatives is reported. The series were generated from diethyl acetylenedicarboxylate and arylhydrazines, which afforded the key intermediates bearing two functional positions. The annellation to generate the maleimide moiety of the bicycle was studied. Moreover, an efficient palladium-catalyzed C-C and C-N bond formation via Suzuki–Miyaura or Buchwald–Hartwig coupling reactions in C-6 position was investigated from 6-chloropyrrolo[3,4-*c*]pyrazole-4,6-(2*H*,5*H*)–diones. This method provides novel access to various 1,6 di-substituted pyrrolo[3,4-*c*] pyrazole-4,6-(2*H*,5*H*)–diones.

## 1. Introduction

**Figure 1:**
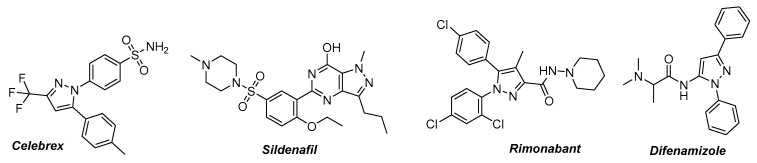
Some examples of pyrazole-based commercial drugs.

Pyrazole derivatives are an important class of five-membered heterocyclic compounds, which are widely encountered as the central core in a large panel of products used in various therapeutic areas such as antibacterial and antifungal agents, antibiotics and pesticides [1,2,3,4,5,6,7,8,9]. For example, the pyrazole ring is present in a variety of drugs such as Celebrex, Sildenafil (Viagra), Rimonabant and Difenamizole (Figure 1). For these reasons, their use as pharmacophores in medicinal chemistry programs has grown, in particular with a view to increasing molecular diversity and exploring innovative chemical spaces.

**Figure 2:**
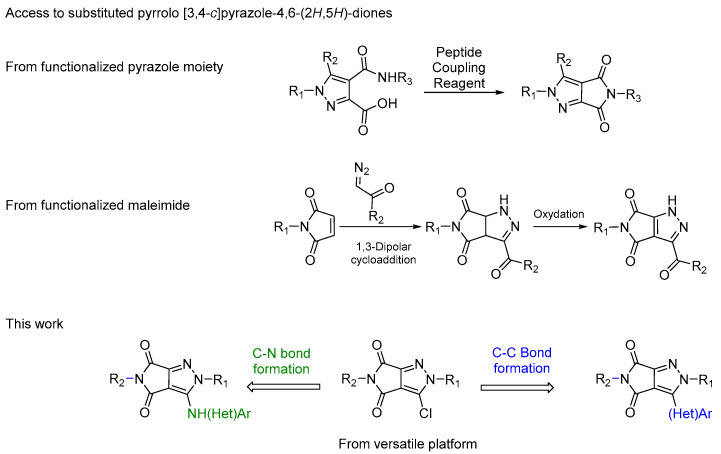
Access to polyfunctionalized pyrrolo[3,4-*c*]pyrazole-4,6-(2*H*,5*H*)-diones.

In contrast, bicyclic heterocycles containing a pyrazole moiety are relatively rare in nature but nonetheless prevalent in the pharmaceutical industry. Such a class is well represented by ring-contracted [5,5] bicyclic aromatic rings [10,11,12,13,14,15,16]. Among this heterocyclic family, the pyrrolo[3,4-*c*]pyrazole-4,6-(2*H*,5*H*)-dione nucleus stands out through the little attention it has been given, despite previous reports of interesting biological activities as a phosphatase inhibitor [16,17,18]. The classic and main method available to date to access this bicyclic system involves building the maleimide moiety using the appropriate functionalized pyrazole moiety [19,20]. Despite the apparent efficiency of this step, molecular diversity cannot be easily managed under this synthetic pathway due to the limitation in terms of access or commercial availability of pyrazole derivatives. In order to introduce a wide range of functional groups and to explore its multiple substitutions, a promising solution is to find an efficient method to selectively functionalize polyfunctionalized pyrrolo[3,4-*c*]pyrazole-4,6-(2*H*,5*H*)-diones at the C-3 position, an indispensable step to designing future original bioactive molecules (Figure 2).

## 2. Results

**Scheme 1:**
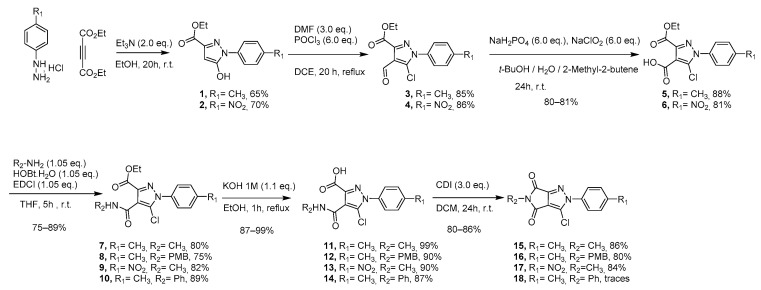
Steps in the synthesis of 2-aryl-3-chloropyrrolo[3,4-*c*]pyrazole-4,6-(*2H*, 5*H*)-diones **15**–**17**.

Based on diethyl acetylenedicarboxylate (DEAD) reactivity with arylhydrazine, new pyrrolo[3,4-*c*]pyrazole-4,6-(2*H*,5*H*)-diones were prepared in a few steps (Scheme 1) [21]. Condensation of the substituted phenylhydrazine chlorhydrate and diethyl acetylenedicarboxylate in ethanol led to 5-hydroxypyrazols **1** and **2** in 65 and 70% yields, respectively. In the next step, treatment of the derivatives **1** and **2** with POCl_3_ and DMF in DCE led to 4-formyl-5-chloropyrazoles **3** and **4** in yields of over 85%. A Pinnick oxidation using sodium chlorite under mild acidic conditions afforded the corresponding acids **5** and **6** in good yields [22,23,24]. An amide bond formation with HOBt and EDCI as peptide coupling reagents [25] was performed in the presence of several amines such as methylamine, aniline or PMBNH_2_ to afford the expected amides **7**–**10**. Saponification of the ester function with KOH furnished acids **11**–**14** in good yields. Finally, the formation of the maleimide moiety was carried out from amines **11**–**14** in the presence of 1,1′-carbonyldiimidazole to access 2-aryl-3-chloropyrrolo[3,4-*c*]pyrazole-4,6-(2*H*,5*H*)-diones **15**–**17** in yields of 80–86% [26]. Only the aromatic N-aryl derivative **18** was never observed, which is a limitation of this annellation method.

With these three compounds in hand, we then achieved the chlorine displacement by Suzuki–Miyaura cross-coupling to explore their reactivity and also access C-3 substituted pyrrolo[3,4-c]pyrazole-4.6-(2H,5H)-diones [27]. This prompted us to propose to the community a general and efficient catalytic system by optimizing the main reaction parameters (Table 1). First, we used **15** as starting material, Pd(OAc)_2_ as the palladium source, Xantphos as a ligand, K_2_CO_3_ as a base and 1.4-dioxane as the solvent under microwave irradiation at 130 °C for 1.5 h [28]. With these conditions, the desired product **19** was isolated in a low but encouraging yield (20%, entry 2), in contrast with PdCl_2_(PPh_3_)_2_ as a catalytic system, which totally inhibited reactivity (entry 1). When the palladium system was switched for Pd(PPh_3_)_4_, the reactivity was improved, and the desired compound **19** was obtained in 65% yield. A fine adjustment of the temperature coupled with an increase in the reaction time improved the reactivity, and the compound was isolated in 85% yield. In the following experiment, we used Cs_2_CO_3_ as a base, which induced a slight decrease in yield. Finally, the nature of the solvent was investigated, showing that toluene induced a drastic inhibition of the reactivity.

Next, the scope and potential limitations of the Pd-coupling step were investigated by modulation of the boron derivatives (Table 2). The use of electron-donating substituents as a methoxy group was well tolerated and afforded the derivative **20** in 79% yield. In contrast, the presence of electron-withdrawing substituents slightly decreased the efficiency of the reaction, and compounds **23** and **24** were isolated in 65% and 60% yields, respectively. Next, we investigated the influence of steric hindrance using the methoxy position switch on the phenyl ring. While the ortho orientation induced a dramatic decrease in yield (only traces of **22** were observed), the meta orientation led to the desired compound **21** in 67% yield. The introduction of electron-rich heterocycles was also studied with 2- or 3-furanyl boronic acids and 2-thienyl boronic acid, and the desired products **25**–**27** were isolated in satisfactory yields. The only identified limit concerned the use of a π-electron-deficient heterocycle such as 4-pyridinyl boronic acid, which drastically inhibited the reaction. Lastly, we evaluated the influence of the nature of the substituent in N-2 and N-5 positions. Remarkably, the presence of PMB substituent in N-5 position preserved the efficiency, and compound **29** was isolated in good yield. The same behavior was observed with a 4-nitrophenyl moiety in N-2 position and afforded **30** in 84% yield.

We next focused our attention on creating a C-N bond instead of a C-C bond under palladium catalysis by chlorine displacement [29]. We started with conditions that had proved their efficiency in the imidazodiazole series [30,31], namely Pd(OAc)_2_/Xantphos as a catalytic system, Cs_2_CO_3_ as a base and dioxane at 130 °C under microwave irradiation. However, in this case, with aniline as a partner, the desired product **31** was isolated in only 8% of yield (Table 3, entry 1). When the catalyst was switched for Pd_2_dba_3_, the reactivity was improved, and the desired compound **31** was obtained in an encouraging 56% yield (entry 2). The fine adjustment of the temperature and reaction time showed that 1h at 100 °C was the best condition, and **31** was isolated in 83% of yield (entry 4). Modifications of the nature of other parameters, such as the base or solvent, did not improve the efficiency of the reaction. Finally, to show that the amination follows a palladium-assisted mechanism without a concomitant S_N_Ar reaction, we carried out the transformation without any catalyst (Table 3, Entry 7), and, as expected, no reaction occurred.

Next, the scope and limitations of the amination were investigated by modulating the nature of the amines (Table 4). The use of electron-rich anilines was well tolerated and afforded derivative **32** in good yields (entries 2). In contrast, the presence of electron-withdrawing substituents such as trifluoromethyl slightly decreased the efficiency of the reaction, and compound **35** was isolated in 41% of yield. We next investigated the influence of steric hindrance using the methoxy position switch on the phenyl ring. While the ortho orientation induced a slight decrease in yield (**34**, 65% versus 32, 88%), the meta orientation did not alter the efficiency of the cross-coupling reaction, as product **33** was isolated in high yield. The only identified limit concerned the nature of the amine. The use of poorly nucleophilic lactams or morpholine as well as secondary alkylamines or 3-aminopyridine was prohibited.

Lastly, the influence of the nature of the substituent in N-2 and N-5 positions was explored. Remarkably, the presence of the PMB substituent in N-5 position or the 4-nitrophenyl moiety in N-1 position led to the same observation, i.e., a slight decrease in the reactivity, and compounds **39** and **40** were isolated in 68% and 51% of yields, respectively.

## 3. Materials and Methods

### 3.1. General Information

^1^H NMR and ^13^C NMR spectra were recorded on a Bruker DPX 400 Mhz instrument using CDCl_3_ and DMSO–*d*_6_. The chemical shifts are reported in parts per million (δ scale), and all coupling constant (J) values are reported in hertz. The following abbreviations were used for the multiplicities: s (singlet), d (doublet), t (triplet), q (quartet), p (pentuplet), m (multiplet), sext (sextuplet) and dd (doublet of doublets). All compounds were characterized by ^1^H NMR, and ^13^C NMR, which are consistent with those reported in the literature (Supplementary Materials). Melting points are uncorrected. IR absorption spectra were obtained on a PerkinElmer PARAGON 1000 PC, and the values are reported in inverse centimeters. HRMS spectra were acquired in positive mode with an ESI source on a Q–TOF mass by the “Fédération de Recherche” ICOA/CBM (FR2708) platform, and NMR data were generated on the Salsa platform. Monitoring of the reactions was performed using silica gel TLC plates (silica Merck 60 F 254). Spots were visualized by using UV light (254 nm and 356 nm). Column chromatography was performed using silica gel 60 (0.063–0.200 mm, Merck, Darmstadt, Germany). Microwave irradiation was carried out in sealed vessels placed in a Biotage Initiator or Biotage Initiator + system (400 W maximum power). The temperatures were measured externally by using IR. Pressure was measured by using a non-invasive sensor integrated into the cavity lid. All reagents were purchased from commercial suppliers and were used without further purification.

### 3.2. Synthesis and Characterization

#### 3.2.1. Ethyl 5-Hydroxy-1-(*p*-tolyl)-1*H*-pyrazole-3-carboxylate (**1**)

To a suspension of *p*-tolylphenylhydrazine hydrochloride (5.0 g, 31.5 mmol, 1.0 eq.) in EtOH (50 mL) was added diethyl acetylenedicarboxylate (6.05 mL, 37.83 mmol, 1.2 eq.) and then slowly Et_3_N (8.72 mL, 63.05 mmol, 2.0 eq.). The mixture was stirred for 20 h at room temperature. The solvent was removed, the residue was taken in EtOAc, and the organic layer was washed with aqueous HCl 6 M. The aqueous layer was extracted twice with EtOAc; organic layers were combined, dried over MgSO_4_, filtrated and concentrated; and the residue was precipitated and washed with Et_2_O to give the title product **1** (2.99 g, 65%) as a white solid. Rf = 0.3 (EtOAc:PE, 8:2). Mp: 194–196 °C. ^1^H NMR (250 MHz, DMSO-*d*_6_) δ 10.36 (s, OH), 7.19 (s, 4H), 6.20 (s, 1H), 4.10 (q, *J* = 7.1 Hz, 2H), 2.31 (s, 3H), 1.11 (t, *J* = 7.1 Hz, 3H). ^13^C NMR (101 MHz, DMSO-*d*_6_) δ 161.3 (CO), 158.9 (CO), 138.1 (Cq), 137.6 (Cq), 133.4 (Cq), 129.3 (2 × CH), 125.7 (2 × CH), 97. 8 (CH), 61.2 (CH_2_), 21.1 (CH_3_), 14.3 (CH_3_). IR (ATR diamond, cm^−1^) ν: 2985, 1722, 1557, 1462, 813, 764, 514. HRMS: *m*/*z* [M + H]^+^ calculated for C_13_H_15_N_2_O_3_: 247.1074, found: 247.1077.

#### 3.2.2. Ethyl 5-Hydroxy-1-(4-nitrophenyl)-1*H*-pyrazole-3-carboxylate (**2**)

To a suspension of 4-nitrophenylhydrazine (4.5 g, 29.40 mmol, 1.00 eq.) in EtOH (50 mL) was added diethyl acetylenedicarboxylate (3.07 mL, 35.28 mmol, 1.2 eq.) and then slowly Et_3_N (8.15 mL, 58.80 mmol, 2.0 eq.). The mixture was stirred for 24 h at room temperature. The solvent was removed, the residue was taken in EtOAc, and the organic layer was washed with aqueous HCl 6 M. The aqueous layer was extracted twice with EtOAc; organic layers were combined, dried over MgSO_4_, filtrated and concentrated; and the residue was precipitated and washed with Et_2_O to give the title product **2** (5.11 g, 70%) as a white solid. Mp: 246–248 °C. ^1^H NMR (400 MHz, DMSO-*d*_6_) δ 8.30 (d, *J* = 8.9 Hz, 2H), 8.08 (d, *J* = 8.9 Hz, 2H), 5.96 (s, 1H), 4.28 (q, *J* = 7.1 Hz, 2H), 1.29 (t, *J* = 7.1 Hz, 3H). ^13^C NMR (101 MHz, DMSO-*d*_6_) *δ* 161.9 (CO), 155.1 (Cq), 145.3 (Cq), 144.1 (Cq), 143.6 (Cq), 125.2 (2 × CH), 121.7 (2 × CH), 90.1 (CH), 60.9 (CH_2_), 14.6 (CH_3_). IR (ATR diamond, cm^−1^) ν: 2955, 1724, 1595, 1421, 1155, 1023, 854, 767. HRMS: *m*/*z* [M + H]^+^ calculated for C_12_H_12_N_3_O_5_: 278.0768, found: 278.0771.

#### 3.2.3. Ethyl 5-Chloro-4-formyl-1-(*p*-tolyl)-1*H*-pyrazole-3-carboxylate (**3**)

To a suspension of compound **1** (2.20 g, 9.01 mmol, 1.0 eq.) in DCE (60 mL) was added DMF (2.13 mL, 34.8 mmol, 3.0 eq.) and POCl_3_ (1.51 mL, 15.76 mmol, 1.75 eq.). The mixture was stirred and refluxed for 1.5 h. After cooling, POCl_3_ (3.8 mL, 39.64 mmol, 4.4 eq.) was added a second time and stirred and refluxed for 18 h. After cooling, water was added slowly, and then the aqueous layer was extracted three times with DCM. Organic layers were combined, dried over MgSO_4_, filtrated and concentrated to give the title product **3** (2.63 g, 85%) as a white solid. Mp: 168–170 °C. ^1^H NMR (400 MHz, CDCl_3_) δ 10.51 (s, 1H_Ald_), 7.41 (d, *J* = 8.4 Hz, 2H), 7.32 (d, *J* = 8.4 Hz, 2H), 4.49 (q, *J* = 7.1 Hz, 2H), 2.43 (s, 3H), 1.43 (t, *J* = 7.1 Hz, 3H). ^13^C NMR (101 MHz, CDCl_3_) δ 185.2 (CH_Ald_), 160.9 (CO), 144.1 (Cq), 140.4 (Cq), 134.0 (Cq), 131.8 (Cq), 129.9 (2 × CH), 125.6 (2 × CH), 119.4 (Cq), 62.1 (CH_2_), 21.3 (CH_3_), 14.3 (CH_3_). IR (ATR diamond, cm^−1^) ν: 2982, 2928, 1740, 1516, 1422, 1259, 1028, 827. HRMS: *m*/*z* [M + H]^+^ calculated for C_14_H_14_ClN_2_O_3_: 293.0685, found: 293.0687.

#### 3.2.4. Ethyl 5-Chloro-4-formyl-1-(4-nitrophenyl)-1*H*-pyrazole-3-carboxylate) (**4**)

To a suspension of compound **2** (3.00 g, 10.13 mmol, 1.0 eq.) in DCE (60 mL) was added DMF (2.4 mL, 30.39 mmol, 3.0 eq.) and POCl_3_ (1.71 mL, 17.72 mmol, 1.75 eq.). The mixture was stirred and refluxed for 1.5 h. After cooling, POCl_3_ (4.27 mL, 44.57 mmol, 4.4 eq.) was added a second time and stirred and refluxed for 18 h. After cooling, water was added slowly and then the aqueous layer was extracted three times with DCM. Organic layers were combined, dried over MgSO_4_, filtrated and concentrated to give the title product **4** (2.63 g, 86%) as a white solid. Mp: 174–176 °C. ^1^H NMR (400 MHz, DMSO-*d*_6_) δ 10.34 (s, H_Ald_), 8.47 (d, *J* = 8.5 Hz, 2H), 8.01 (d, *J* = 8.5 Hz, 2H), 4.42 (q, *J* = 7.1 Hz, 2H), 1.35 (t, *J* = 7.1 Hz, 3H). ^13^C NMR (101 MHz, DMSO-*d*_6_) δ 185.4 (CH_Ald_), 160.8 (CO), 148.3 (Cq), 145.0 (Cq), 141.3 (Cq), 132.1 (Cq), 127.7 (2 × CH), 125.4 (2 × CH), 119.7 (Cq), 62.3 (CH_2_), 14.5 (CH_3_).IR (ATR diamond, cm^−1^) ν: 3115, 2988, 1723, 1535, 1321, 1025, 860, 687. HRMS: *m*/*z* [M + H]^+^ calculated for C_13_H_11_ClN_3_O_5_: 324.0378, found: 324.0381.

#### 3.2.5. 5-Chloro-3-(ethoxycarbonyl)-1-(*p*-tolyl)-1*H*-pyrazole-4-carboxylic acid (**5**)

To a suspension of **3** (2.63 g, 9.01 mmol, 1.0 eq.) in a mixture of *t*-BuOH/H_2_O/2-methyl-2-butene (45 mL/45 mL/27 mL) was added NaH_2_PO_4_ (6.48 g, 54.06 mmol, 6.0 eq.) and NaClO_2_ (4.89 g, 54.06 mmol, 6.0 eq.). The mixture was stirred for 24 h at room temperature. Then, the mixture was poured into a funnel with EtOAc (50 mL) and water (30 mL). The aqueous layer was extracted twice with EtOAc. The aqueous layer was acidified with HCl 12 M, and the precipitate was filtrated, washed with cold water and dried with Et_2_O. Organics layers were combined, dried over MgSO_4_ and concentrated; the residue was triturated in EtOAc (2 mL); and Petroleum Ether (30 mL) was added. The resulting precipitate was filtrated and combined with the first solid to give the title compound **5** (2.77 g, 88%) as a white solid. Mp: 172–174 °C. ^1^H NMR (400 MHz, DMSO-*d*_6_) δ 7.43 (d, *J* = 8.4 Hz, 2H), 7.38 (d, *J* = 8.4 Hz, 2H), 4.26 (q, *J* = 7.1 Hz, 2H), 2.40 (s, 3H), 1.27 (t, *J* = 7.1 Hz, 3H). ^13^C NMR (101 MHz, DMSO-*d*_6_) δ 165.4 (CO), 163.1 (CO), 144.1 (Cq), 139.4 (Cq), 135.4 (Cq), 130.2 (2 × CH), 126.6 (Cq), 125.9 (2 × CH), 121.4 (Cq), 61.2 (CH_2_), 21.2 (CH_3_), 14.5 (CH_3_). IR (ATR diamond, cm^−1^) ν: 3011, 2752, 1734, 1452, 1300, 1223, 1027, 826, 763. HRMS: *m*/*z* [M + H]^+^ calculated for C_14_H_14_ClN_2_O_4_: 309.0999, found: 309.1000.

#### 3.2.6. 5-Chloro-3-(ethoxycarbonyl)-1-(4-nitrophenyl)-1*H*-pyrazole-4-carboxylic acid (**6**)

To a suspension of **4** (2.00 g, 6.22 mmol, 1.00 eq.) in a mixture of t-BuOH/H_2_O/2-methyl-2-butene (45 mL/45 mL/27 mL) was added NaH_2_PO_4_ (4.48 g, 37.37 mmol, 6.00 eq.) and NaClO_2_ (3.38 g, 37.37 mmol, 6.00 eq.). The mixture was stirred for 24 h at room temperature. Then, the mixture was poured into a funnel with EtOAc (50 mL) and water (30 mL). The aqueous layer was extracted twice with EtOAc. The aqueous layer was acidified with HCl 12 M, and the precipitate was filtrated, washed with cold water and dried with Et_2_O. Organic layers were combined, dried over MgSO_4_ and concentrated; the residue was triturated in EtOAc (2 mL); and Petroleum Ether (30 mL) was added. The resulting precipitate was filtrated and combined with the first solid to give the title compound **6** (1.04g, 81%) as a white solid. Mp: 174–176 °C. ^1^H NMR (250 MHz, DMSO-*d*_6_) δ 8.44 (d, *J* = 8.9 Hz, 2H), 7.99 (d, *J* = 8.9 Hz, 2H), 4.34 (q, *J* = 7.1 Hz, 2H), 1.29 (t, *J* = 7.1 Hz, 3H). ^13^C NMR (101 MHz, DMSO-*d*_6_) δ 161.9 (CO), 161.5 (CO), 148.1 (Cq), 145.5 (Cq), 141.8 (Cq), 131.1 (Cq), 127.4 (2 × CH), 125.3 (2 × CH), 114.2 (Cq), 62.2 (CH_2_), 14.3 (CH_3_). IR (ATR diamond, cm^−1^) ν: 3086, 2662, 1746, 1414, 1302, 1234, 852, 753. HRMS: *m*/*z* [M + H]^+^ calculated for C_13_H_11_ClN_3_O_6_: 340.0331, found: 340.0330.

#### 3.2.7. Ethyl 5-Chloro-4-(methylcarbamoyl)-1-(*p*-tolyl)-1*H*-pyrazole-3-carboxylate (**7**)

To a suspension of **5** (2.00 g, 6.49 mmol, 1.00 eq.) in THF (30 mL) was added HOBt·H_2_O (1.043 g, 7.78 mmol, 1.20 eq.), methylamine (3.4 mL, 6.81 mmol, 1.05 eq.) and then EDCI (1.19, 7.13 mmol, 1.10 eq.). The mixture was stirred for 5 h at room temperature. Then, Et_2_O (40 mL) was added, and the precipitate was filtered, washed with EtOAc and dried under vacuum to give **7** (1.66 g, 80%) as a white solid. Mp: 190–192 °C. ^1^H NMR (400 MHz, CDCl_3_) δ 9.02 (q, *J* = 4.7 Hz, 1H), 7.37 (d, *J* = 8.2 Hz, 2H), 7.30 (d, *J* = 8.2 Hz, 2H), 4.47 (q, *J* = 7.1 Hz, 2H), 2.99 (d, *J* = 4.7 Hz, 3H), 2.43 (s, 3H), 1.43 (t, *J* = 7.1 Hz, 3H). ^13^C NMR (101 MHz, CDCl_3_) δ 163.8 (CO), 160.9 (CO), 140.2 (Cq), 139.9 (Cq), 134.6 (Cq), 133.6 (Cq), 129.8 (2 × CH), 125.9 (2 × CH), 116.3 (Cq), 62.6 (CH_2_), 26.2 (NCH_3_), 21.3 (CH_3_), 14.2 (CH_3_). IR (ATR diamond, cm^−1^) ν: 3295, 1722, 1642, 1568, 1315, 1230, 1120, 1030, 826. HRMS: *m*/*z* [M + H]^+^ calculated for C_15_H_17_ClN_3_O_3_: 322.0958, found: 322.0952.

#### 3.2.8. Ethyl 5-Chloro-4-[(4-methoxyphenyl)methylcarbamoyl]-1-(*p*-tolyl)-1*H*-pyrazole-3-carboxylate (**8**)

To a suspension of **5** (1.00 g, 3.25 mmol, 1.00 eq.) in THF (30 mL) was added HOBt·H_2_O (0.50 g, 3.89 mmol, 1.20 eq.), 4-methoxybenzylamine (0.50 mL, 3.41 mmol, 1.05 eq.) and then EDCI (0.59 mL, 3.36 mmol, 1.10 eq.). The mixture was stirred for 5 h at room temperature. Then, Et2O (40 mL) was added, and the precipitate was filtered, washed with EtOAc and dried under vacuum to give **8** (0.80 g, 75%) as a white solid. Mp: 168–170 °C. ^1^H NMR (400 MHz, CDCl_3_) δ 9.44 (t, *J* = 5.5 Hz, 1H), 7.40 (d, *J* = 8.1 Hz, 2H), 7.37–7.30 (m, 4H), 6.90 (d, *J* = 8.1 Hz, 2H), 4.60 (d, *J* = 5.5 Hz, 2H), 4.46 (q, *J* = 7.1 Hz, 2H), 3.82 (s, 3H), 2.45 (s, 3H), 1.42 (t, *J* = 7.1 Hz, 3H). ^13^C NMR (101 MHz, CDCl_3_) δ 163.6 (CO), 160.0 (CO), 158.9 (Cq), 140.2 (Cq), 140.0 (Cq), 134.6 (Cq), 133.8 (Cq), 130.6 (Cq), 129.8 (2 × CH), 129.2 (2 × CH), 125.9 (2 × CH), 116.2 (Cq), 114.0 (2 × CH), 62.6 (CH_2_), 55.3 (OCH_3_), 43.0 (NCH_2_), 21.3 (CH_3_), 14.2 (CH_3_). IR (ATR diamond, cm^−1^) ν: 3557, 3304, 1721, 1636, 1302, 1255, 1041, 854, 838. HRMS: *m*/*z* [M + H]^+^ calculated for C_22_H_23_ClN_3_O_4_: 428.1369, found: 428.1371.

#### 3.2.9. Ethyl 5-Chloro-4-(methylcarbamoyl)-1-(4-nitrophenyl)-1*H*-pyrazole-3-carboxylate (**9**)

To a suspension of **6** (1.00 g, 3.05 mmol, 1.00 eq.) in THF (30 mL) was added HOBt·H2O (0.55 g, 3.65 mmol, 1.20 eq.), methylamine (1.60 mL, 3.20 mmol, 1.05 eq.) and then EDCI (0.79 mL, 4.42 mmol, 1.10 eq.). The mixture was stirred for 5 h at room temperature. Then, Et2O (40 mL) was added, and the precipitate was filtered, washed with EtOAc and dried under vacuum to give **9** (1.902 g, 82%) as a white solid. Mp: 156–158 °C. ^1^H NMR (250 MHz, DMSO-*d*_6_) δ 8.41–8.49 (m, 3H), 7.97 (d, *J* = 8.5 Hz, 2H), 4.31 (q, *J* = 6.8 Hz, 2H), 2.77 (d, *J* = 4.1 Hz, 3H), 1.28 (t, *J* = 6.8 Hz, 3H). ^13^C NMR (101 MHz, DMSO-*d*_6_) δ 160.8 (CO), 160.6 (CO), 147.9 (Cq), 142.3 (Cq), 141.9 (Cq), 128.2 (Cq), 126.9 (2 × CH), 125.4 (2 × CH), 120.4 (Cq), 61.7 (CH_2_), 26.4 (NCH_3_), 14.4 (CH_3_). IR (ATR diamond, cm^−1^) ν: 3086, 2662, 1746, 1414, 12341, 1157, 1040, 836. HRMS: *m*/*z* [M + H]^+^ calculated for C_14_H_14_ClN_4_O_5_: 353.0645, found: 353.0647.

#### 3.2.10. Ethyl 5-Chloro-4-(phenylcarbamoyl)-1-(*p*-tolyl)-1*H*-pyrazole-3-carboxylate (**10**)

To a suspension of **5** (1.00 g, 3.25 mmol, 1.00 eq.) in THF (30 mL) was added HOBt·H_2_O (0.50 g, 3.89 mmol, 1.20 eq.), phenylamine (0.55 mL, 3.41 mmol, 1.05 eq.) and then EDCI (0.59 mL, 3.36 mmol, 1.10 eq.). The mixture was stirred for 5 h at room temperature. Then, Et_2_O (40 mL) was added, and the precipitate was filtered, washed with EtOAc and dried under vacuum to give **10** (1.1 g, 89%) as a white solid. Mp: 176–178 °C. ^1^H NMR (400 MHz, CDCl_3_) δ 11.41 (s, 1H), 7.78 (d, *J* = 7.9 Hz, 2H), 7.37 (m, 6H), 7.13 (t, *J* = 7.9 Hz, 1H), 4.54 (q, *J* = 7.1 Hz, 2H), 2.45 (s, 3H), 1.46 (t, *J* = 7.1 Hz, 3H). ^13^C NMR (101 MHz, CDCl_3_) δ 164.4 (CO), 158.0 (CO), 140.3 (Cq), 139.6 (Cq), 138.4 (Cq), 134.7 (Cq), 134.5 (Cq), 129.9 (2 x CH), 128.9 (2 × CH), 126.0 (2 × CH), 124.2 (CH), 120.1 (2 × CH), 116.5 (Cq), 63.0 (CH_2_), 21.3 (CH_3_), 14.2 (CH_3_). IR (ATR diamond, cm^−1^) ν: 3134, 3274, 172Ç, 1636, 1354, 1195, 1044, 879, 889. HRMS (EI-MS): *m*/*z* calculated for C_20_H_19_ClN_3_O_3_: 384.1013 [M + H]^+^, found: 384.1017.

#### 3.2.11. 5-Chloro-4-(methylcarbamoyl)-1-(*p*-tolyl)-1*H*-pyrazole-3-carboxylic acid (**11**)

To a suspension of **7** (0.56 g, 1.75 mmol, 1.0 eq.) in EtOH (10 mL) was added a KOH aqueous solution, 1M (1.93 mL, 1.93 mmol, 1.1 eq.). The mixture was refluxed for 1 h, and after cooling, the solvent was removed partially and then poured into three volumes of cold water. The aqueous mixture was acidified with HCl 12 M and then the precipitate was filtered off and then solubilized in EtOAc. The organic filtrate was dried over MgSO_4_, filtered and concentrated to give the title compound **11** (0.51 g, 99%) as a white solid. Mp: 228–230 °C. ^1^H NMR (400 MHz, DMSO-*d*_6_) δ 8.70 (q, *J* = 3.9 Hz, 1NH), 7.46 (d, *J* = 8.1 Hz, 2H), 7.40 (d, *J* = 8.1 Hz, 2H), 2.76 (d, *J* = 3.9 Hz, 3H), 2.40 (s, 3H). ^13^C NMR (400 MHz, DMSO-*d*_6_) δ 162.7 (CO), 161.41 (CO), 142.4 (Cq), 140.0 (Cq), 134.9 (Cq), 130.3 (2 × CH), 128.3 (Cq), 126.0 (2 × CH), 118.6 (Cq), 26.4 (NCH_3_), 21.2 (CH_3_). IR (ATR diamond, cm^−1^) ν: 3368, 1731, 1558, 1257, 1030, 824, 650. HRMS: *m*/*z* [M + H]^+^ calculated for C_13_H_13_ClN_3_O_3_: 294.0640, found: 294.0639.

#### 3.2.12. 5-Chloro-4-((4-methoxybenzyl)carbamoyl)-1-(*p*-tolyl)-1*H*-pyrazole-3-carboxylic acid (**12**)

To a suspension of **8** (1.34 g, 3.25 mmol, 1.0 eq.) in EtOH (10 mL) was added a KOH aqueous solution, 1M (2.83 mL, 3.57 mmol, 1.1 eq.). The mixture was refluxed for 1 h, and after cooling, the solvent was removed partially and then poured into three volumes of cold water. The aqueous mixture was acidified with HCl 12 M, and then the precipitate was filtered off and then solubilized in EtOAc. The organic filtrate was dried over MgSO_4_, filtered and concentrated to give the title compound **12** (1.19 g, 90%) as a white solid. Mp: 202–204 °C. ^1^H NMR (250 MHz, DMSO-*d*_6_) δ 12.11 (t, *J* = 5.3 Hz, 1NH), 7.30–735 (m, 4H), 7.26 (d, *J* = 8.2 Hz, 2H), 6.89 (d, *J* = 8.2 Hz, 2H), 4.39 (d, *J* = 5.3 Hz, 2H), 3.73 (s, 3H), 2.36 (s, 3H). ^13^C NMR (400 MHz, DMSO-*d*_6_) δ 164.0 (CO), 160.7 (CO), 158.60 (Cq), 139.7 (Cq), 134.7 (Cq), 134.6 (Cq), 131.9 (Cq), 130.0 (2 × CH), 129.9 (Cq), 129.1 (2 × CH), 126.7 (Cq), 126.6 (2 × CH), 114.2 (2 × CH), 55.5 (OCH_3_), 42.1 (NCH_2_), 21.2 (CH_3_). IR (ATR diamond, cm^−1^) ν: 3347, 1752, 1560, 1338, 1176, 1001, 856, 765. HRMS: *m*/*z* [M + H]^+^ calculated for C_20_H_19_ClN_3_O_4_: 400.1059, found: 400.1058.

#### 3.2.13. 5-Chloro-4-(methylcarbamoyl)-1-(4-nitrophenyl)-1*H*-pyrazole-3-carboxylic acid (**13**)

To a suspension of **9** (0.470 g, 1.38 mmol, 1.0 eq.) in EtOH (10 mL) was added a KOH aqueous solution, 1M (1.59 mL, 1.51 mmol, 1.1 eq.). The mixture was refluxed for 1 h, and after cooling, the solvent was removed partially and then poured into three volumes of cold water. The aqueous mixture was acidified with HCl 12N, and then the precipitate was filtered off and then solubilized in EtOAc. The organic filtrate was dried over MgSO_4_, filtered and concentrated to give the title compound **13** (0.45 g, 90%) as a white solid. Mp: 282–264 °C. ^1^H NMR (250 MHz, DMSO-*d*_6_) δ 8.54 (q, *J* = 4.5 Hz, 1NH), 8.46 (d, *J* = 8.6 Hz, 2H), 7.97 (d, *J* = 8.6 Hz, 2H), 2.77 (d, *J* = 4.5 Hz, 3H). ^13^C NMR (400 MHz, DMSO-*d*_6_) δ 162.3 (CO), 161.0 (CO), 147.9 (Cq), 143.2 (Cq), 142.0 (Cq), 128.3(Cq), 126.9 (2 × CH), 125.4 (2 × CH), 120.0 (Cq), 26.5 (NCH_3_). IR (ATR diamond, cm^−1^) ν: 3128, 2924, 1767, 1606, 1500, 1356, 1005, 606. HRMS: *m*/*z* [M + H]^+^ calculated for C_12_H_10_ClN_4_O_5_: 325.0332, found: 325.0334.

#### 3.2.14. 5-Chloro-4-(phenylcarbamoyl)-1-(*p*-tolyl)-1*H*-pyrazole-3-carboxylic acid (**14**)

To a suspension of **10** (1.1 g, 2.87 mmol, 1.0 eq.) in EtOH (10 mL) was added a KOH aqueous solution, 1M (3.16 mL, 3.16 mmol, 1.1 eq.). The mixture was refluxed for 1 h, and after cooling, the solvent was removed partially and then poured into three volumes of cold water. The aqueous mixture was acidified with HCl 12 M, and then the precipitate was filtered off and then solubilized in EtOAc. The organic filtrate was dried over MgSO_4_, filtered and concentrated to give the title compound **14** (886 mg, 87%) as a white solid. Mp: 218–220 °C. ^1^H NMR (250 MHz, DMSO-*d*_6_) δ 10.81 (s, 1H), 7.69 (d, *J* = 7.9 Hz, 2H), 7.51 (d, *J* = 8.0 Hz, 2H), 7.43 (d, *J* = 8.0 Hz, 2H), 7.36 (t, *J* = 7.9 Hz, 2H), 7.12 (t, *J* = 7.9 Hz, 1H), 2.42 (s, 3H). ^13^C NMR (101 MHz, CDCl_3_) δ 162.6 (CO), 159.4 (CO), 142.2 (Cq), 140.1 (Cq), 139.5 (Cq), 134.9 (Cq), 130.4 (2 × CH), 129.3 (2 × CH), 128.5 (Cq), 125.9 (2 × CH), 124.2 (CH), 119.7 (2 × CH), 119.3 (Cq), 21.2 (CH_3_). IR (ATR diamond, cm^−1^) ν: 3145, 1761, 1569, 1588, 1170, 1007, 876, 744. HRMS (EI-MS): *m*/*z* calculated for C_18_H_15_ClN_3_O_3_: 356.0747 [M + H]^+^, found: 356.0749.

#### 3.2.15. 3-Chloro-5-methyl-2-(*p*-tolyl)pyrrolo[3,4-*c*]pyrazole-4,6-(2*H*,5*H*)-dione (**15**)

To a suspension of **11** (0.50 g, 1.7 mmol, 1.0 eq.) in dry DCM (10 mL) under inert gas was added CDI (0.830 g, 5.11 mmol, 3.0 eq.). The mixture was stirred for 24 h at room temperature. The solvent was removed, and the crude was purified by using flash chromatography with EP/EtOAc (9/1) as eluent to give **15** (0.403 g, 86%) as a white solid. Mp: 208–210 °C. ^1^H NMR (400 MHz, CDCl_3_) δ 7.44 (d, *J* = 8.3 Hz, 2H), 7.34 (d, *J* = 8.3 Hz, 2H), 3.15 (s, 3H), 2.45 (s, 3H). ^13^C NMR (101 MHz, CDCl_3_) δ 161.1 (CO), 160.5 (CO), 152.4 (Cq), 140.4 (Cq), 134.5 (Cq), 130.0 (2 × CH), 125.3 (2 × CH), 125.1 (Cq), 116.7 (Cq), 24.5 (NCH_3_), 21.3 (CH_3_). IR (ATR diamond, cm^−1^) ν: 3385, 1715, 1556, 1530, 1357, 1260, 1136, 1177. HRMS: *m*/*z* [M + H]^+^ calculated for C_13_H_11_ClN_3_O_2_: 276.0536, found: 276.0534.

#### 3.2.16. 3-Chloro-5-(4-methoxybenzyl)-2-(*p*-tolyl)pyrrolo[3,4-*c*]pyrazole-4,6-(2*H*,5*H*)-dione (**16**)

To a suspension of **12** (0.4g, 1.00 mmol, 1.0 eq.) in dry DCM (10 mL) under inert gas was added CDI (0.486 g, 3.00 mmol, 3.0 eq.). The mixture was stirred for 24 h at room temperature. The solvent was removed, and the crude was purified by using flash chromatography with EP/EtOAc (7/3) as eluent to give **16** (0.306 g, 80%) as a white solid. Mp: 158–160 °C. ^1^H NMR (400 MHz, CDCl_3_) δ 7.41 (d, *J* = 8.1 Hz, 2H), 7.37 (d, *J* = 8.4 Hz, 2H), 7.32 (d, *J* = 8.1 Hz, 2H), 6.84 (d, *J* = 8.4 Hz, 2H), 4.73 (s, 2H), 3.78 (s, 3H), 2.44 (s, 3H). ^13^C NMR (101 MHz, CDCl_3_) δ 160.5 (CO), 159.9 (CO), 159.1 (Cq), 152.0 (Cq), 140.2 (Cq), 134.2 (Cq), 130.1 (2 × CH), 129.8 (2 × CH), 128.4 (Cq), 125.1 (2 × CH), 125.0 (Cq), 116.4 (Cq), 113.8 (2 × CH), 55.1 (OCH_3_), 41.3 (NCH_2_), 21.1 (CH_3_). IR (ATR diamond, cm^−1^) ν: 2934, 2838, 1710, 1576, 1241, 1030, 916, 816, 760. HRMS: *m*/*z* [M + H]^+^ calculated for C_20_H_17_ClN_3_O_3_: 382.0952, found: 382.0952.

#### 3.2.17. 3-Chloro-5-methyl-2-(4-nitrophenyl)pyrrolo[3,4-*c*]pyrazole-4,6-(2*H*,5*H*)-dione (**17**)

To a suspension of **13** (0.5g, 1.54 mmol, 1.0 eq.) in dry DCM (10 mL) under inert gas was added CDI (0.750 g, 4.62 mmol, 3.0 eq.). The mixture was stirred for 24 h at room temperature. The solvent was removed, and the crude was purified by flash chromatography with EP/EtOAc (7/3) as eluent to give **17** (0.366 g, 84%) as a white solid. Mp: 206–208 °C. ^1^H NMR (400 MHz, CDCl_3_) δ 8.46 (d, *J* = 8.7 Hz, 2H), 7.90 (d, *J* = 8.7 Hz, 2H), 3.20 (s, 3H). ^13^C NMR (101 MHz, CDCl_3_) δ 160.4 (CO), 159.7 (CO), 153.3 (Cq), 148.0 (Cq), 141.6 (Cq), 125.8 (2 × CH), 125.2 (Cq), 124.9 (2 × CH), 117.7 (Cq), 24.7 (CH_3_). IR (ATR diamond, cm^−1^) ν: 3120, 3008, 1731, 1577, 1530, 1350, 982, 733, 674. HRMS: *m*/*z* [M + H]^+^ calculated for C_12_H_8_ClN_4_O_4_: 307.6605, found: 307.6610.

#### 3.2.18. 5-Methyl-2,3-di-*p*-tolylpyrrolo[3,4-*c*]pyrazole-4,6-(2*H*,5*H*)-dione (**19**)

In a microwave vial, 0.5–2 mL with a stir bar was charged: **15** (0.05 g, 0.18 mmol, 1.00 eq.), *p*-tolylboronic acid (0.037 g, 0.27 mmol, 1.5 eq.), K_2_CO_3_ (0.075 g, 0.054, 3.0 eq.) and dry dioxane (3.0 mL). The mixture was degassed for 15 min, and then Pd(PPh_3_)_4_ (0.021 g, 0.018 mmol, 0.10 eq.) was added. The vial was sealed and then put in a microwave cavity. After 2 h of irradiation at 150 °C, the mixture was concentrated and purified by using flash chromatography with Petroleum Ether/EtOAc (1/9) as eluent to give **19** (0.048 g, 85%) as a white solid. M.p: 170–172 °C. ^1^H NMR (400 MHz CDCl_3_) δ 7.37 (d, *J* = 8.1 Hz, 2H), 7.27 (d, *J* = 8.1 Hz, 2H), 7.24 (d, *J* = 8.1 Hz, 2H), 7.18 (d, *J* = 8.1 Hz, 2H), 3.17 (s, 3H), 2.43 (s, 3H), 2.38 (s, 3H). ^13^C NMR (101 MHz, CDCl_3_) δ 162.6 (CO), 162.11 (CO), 152.5 (Cq), 141.3 (Cq), 140.6 (Cq), 139.5 (Cq), 137.0 (Cq), 130.0 (2 × CH), 129.5 (2 × CH), 129.2 (2 × CH), 125.6 (2 × CH), 123.7 (Cq), 117.2 (Cq), 24.3 (NCH_3_), 21.4 (CH_3_), 21.2 (CH_3_). IR (ATR diamond, cm^−1^) ν: 2922, 2851, 1760, 1703, 1513, 1356, 1019, 851, 793. HRMS: *m*/*z* [M + H]^+^ calculated for C_20_H_18_N_3_O_2_: 332.1397, found: 332.1393.

#### 3.2.19. 3-(4-Methoxyphenyl)-5-methyl-2-(*p*-tolyl)pyrrolo[3,4-*c*]pyrazole-4,6-(2*H*,5*H*)-dione (**20**)

In a microwave vial, 0.5–2 mL with a stir bar was charged: **15** (0.05 g, 0.18 mmol, 1.00 eq.), 4-methoxyphenylboronic acid (0.042 g, 0.27 mmol, 1.5 eq.), K_2_CO_3_ (0.075 g, 0.054, mmol, 3.0 eq.) and dry dioxane (3.0 mL). The mixture was degassed for 15 min, and then Pd(PPh_3_)_4_ (0.021 g, 0.018 mmol, 0.10 eq.) was added. The vial was sealed and then put in a microwave cavity. After 2 h of irradiation at 150 °C, the mixture was concentrated and purified by using flash chromatography with Petroleum Ether/EtOAc (1/9) as eluent to give **20** (0.048 g, 79%) as a white solid. M.p: 150–152 °C. ^1^H NMR (400 MHz, CDCl_3_) δ 7.37 (d, *J* = 8.8 Hz, 2H), 7.21 (d, *J* = 8.8 Hz, 2H), 7.18 (d, *J* = 8.8 Hz, 2H), 6.81 (d, *J* = 8.8 Hz, 2H), 3.77 (s, 3H), 3.10 (s, 3H), 2.37 (s, 3H). ^13^C NMR (101 MHz, CDCl_3_) δ 162.7 (CO), 162.1 (CO), 161.0 (Cq), 152.5 (Cq), 141.2 (Cq), 139.5 (Cq), 137.1 (Cq), 130.9 (2 × CH), 130.0 (2 × CH), 125.7 (2 × CH), 118.8 (Cq), 116.7 (Cq), 114.2 (2 × CH), 55.35 (OCH_3_), 24.3 (NCH_3_), 21.3 (CH_3_). IR (ATR diamond, cm^−1^) ν: 2923, 2848, 1763, 1700, 1499, 990, 1180, 803, 517. HRMS: *m*/*z* [M + H]^+^ calculated for C_20_H_18_N_3_O_3_: 348.1343, found: 348.1342.

#### 3.2.20. 3-(3-Methoxyphenyl)-5-methyl-2-(*p*-tolyl)pyrrolo[3,4-*c*]pyrazole-4,6-(2*H*,5*H*)-dione (**21**)

In a microwave vial, 0.5–2 mL with a stir bar was charged: **15** (0.05 g, 0.18 mmol, 1.00 eq.), 3-methoxyphenylboronic acid (0.042 g, 0.27 mmol, 1.5 eq.), K_2_CO_3_ (0.075 g, 0.054, mmol, 3.0 eq.) and dry dioxane (3.0 mL). The mixture was degassed for 15 min, and then Pd(PPh_3_)_4_ (0.021 g, 0.018 mmol, 0.10 eq.) was added. The vial was sealed and then put in a microwave cavity. After 2 h of irradiation at 150 °C, the mixture was concentrated and purified by using flash chromatography with Petroleum Ether/EtOAc (1/9) as eluent to give **21** (0.046 g, 67%) as a white solid. M.p: 168–170 °C. ^1^H NMR (400 MHz, CDCl_3_) δ 7.31–7.28 (m, 3H), 7.26 (d, *J* = 8.0 Hz, 2H), 7.15 (dd, *J* = 2.6, 1.6 Hz, 1H), 6.99–6.93 (m, 2H), 3.75 (s, 3H), 3.18 (s, 3H), 2.44 (s, 3H). ^13^C NMR (101 MHz, CDCl_3_) δ 162.8 (CO), 162.3 (CO), 159.9 (Cq), 152.8 (Cq), 141.4 (Cq), 140.0 (Cq), 137.3 (Cq), 130.3 (2 × CH), 130.1 (CH), 127.9 (Cq), 126.0 (2 × CH), 121.7 (CH), 117.9 (Cq), 117.2 (CH), 114.5 (CH), 55.6 (OCH_3_), 24.6 (NCH_3_), 21.6 (CH_3_). IR (ATR diamond, cm^−1^) ν: 2929, 2921, 1758, 1705, 1448, 1360, 962, 787, 547 HRMS: *m*/*z* [M + H]^+^ calculated for C_20_H_18_N_3_O_3_: 348.1460, found: 348.1343.

#### 3.2.21. 3-(4-Fluorophenyl)-5-methyl-2-(*p*-tolyl)pyrrolo[3,4-*c*]pyrazole-4,6-(2*H*,5*H*)-dione (**23**)

In a microwave vial, 0.5–2 mL with a stir bar was charged: **15** (0.05 g, 0.18 mmol, 1.00 eq.), 4-fluorophenylboronic acid (0.038 g, 0.27 mmol, 1.5 eq.), K_2_CO_3_ (0.075 g, 0.054 mmol, 3.0 eq.) and dry dioxane (3.0 mL). The mixture was degassed for 15 min, and then Pd(PPh_3_)_4_ (0.021 g, 0.018 mmol, 0.10 eq.) was added. The vial was sealed and then put in a microwave cavity. After 2 h of irradiation at 150 °C, the mixture was concentrated and purified by using flash chromatography with Petroleum Ether/EtOAc (1/9) as eluent to give **23** (0.038 g, 65%) as a white solid. M.p: 178–180 °C. ^1^H NMR (250 MHz, Chloroform-*d*) δ 7.50–7.41 (m, 2H), 7.22–7.24 (m, 4H), 7.12–6.97 (m, 2H), 3.15 (s, 3H), 2.42 (s, 3H). ^13^C NMR (63 MHz, CDCl_3_) δ 163.49 (d, *J* = 252.5 Hz, Cq), 162.4 (CO), 161.9 (CO), 152.5 (Cq), 140.0 (Cq), 139.8 (Cq), 136.7 (Cq), 131.5 (d, *J* = 8.7 Hz, 2 × CH), 130.1 (2 × CH), 125.6 (2 × CH), **122**.7 (d, *J* = 3.5 Hz, Cq), 117.5 (Cq), 116.1 (d, *J* = 22.1 Hz, 2 × CH), 24.3 (NCH_3_), 21.2 (CH_3_). IR (ATR diamond, cm^−1^) ν: 2924, 2920, 1767, 1710, 1448, 1356, 1107, 879, 643, 532. HRMS: *m*/*z* [M + H]^+^ calculated for C_19_H_15_FN_3_O_2_: 336.1144, found: 336.1142.

#### 3.2.22. 3-(4-Cyanophenyl)-5-methyl-2-(*p*-tolyl)pyrrolo[3,4-*c*]pyrazole-4,6-(2*H*,5*H*)-dione (**24**)

In a microwave vial, 0.5–2 mL with a stir bar was charged: **15** (0.050 g, 0.18 mmol, 1.00 eq.), 4-cyanophenylboronic acid (0.053 g, 0.27 mmol, 1.5 eq.), K_2_CO_3_ (0.075 g, 0.054 mmol, 3.0 eq.) and dry dioxane (3.0 mL). The mixture was degassed for 15 min, and then Pd(PPh_3_)_4_ (0.021 g, 0.018 mmol, 0.10 eq.) was added. The vial was sealed and then put in a microwave cavity. After 2 h of irradiation at 150 °C, the mixture was concentrated and purified by using flash chromatography with Petroleum Ether/EtOAc (1/9) as eluent to give **24** (0.035 g, 60%) as a white solid. M.p: 178–180 °C. ^1^H NMR (250 MHz, CDCl_3_) δ 7.65 (d, *J* = 8.5 Hz, 2H), 7.59 (d, *J* = 8.5 Hz, 2H), 7.27 (d, *J* = 8.5 Hz, 2H), 7.22 (d, *J* = 8.5 Hz, 2H), 3.17 (s,3H), 2.44 (s, 3H). ^13^C NMR (63 MHz, CDCl_3_) δ 162.1 (CO), 161.5 (CO), 152.7 (Cq), 140.4 (Cq), 138.6 (Cq), 136.3 (Cq), 132.5 (2 × CH), 130.8 (Cq), 130.4 (2 × CH), 129.8 (2 × CH), 125.6 (2 × CH), 118.7 (Cq), 117.9 (Cq), 113.7 (Cq), 24.5 (NCH_3_), 21.3 (CH_3_). IR (ATR diamond, cm^−1^) ν: 2923, 2227, 1764, 1709, 1494, 1352, 1279, 1105, 973, 643, 551. HRMS: *m*/*z* [M + H]^+^ calculated for C_20_H_15_N_4_O_2_: 343.1192, found: 343.1189.

#### 3.2.23. 3-(FuraN-2-yl)-5-methyl-2-(*p*-tolyl)pyrrolo[3,4-*c*]pyrazole-4,6-(2*H*,5*H*)-dione (**25**)

In a microwave vial, 0.5–2 mL with a stir bar was charged: **15** (0.05 g, 0.18 mmol, 1.00 eq.), 2-furanylboronic acid (0.030 g, 0.27 mmol, 1.5 eq.), K_2_CO_3_ (0.075 g, 0.054 mmol, 3.0 eq.) and dry dioxane (3.0 mL). The mixture was degassed for 15 min, and then Pd(PPh_3_)_4_ (0.021 g, 0.018 mmol, 0.10 eq.) was added. The vial was sealed and then put in a microwave cavity. After 2 h of irradiation at 150 °C, the mixture was concentrated and purified by using flash chromatography with Petroleum Ether/EtOAc (1/9) as eluent to give **25** (0.042 g, 76%) as a white solid. M.p: 172–174 °C. ^1^H NMR (400 MHz CDCl_3_) δ 7.46 (d, *J* = 1.8 Hz, 1H), 7.38 (d, *J* = 8.2 Hz, 2H), 7.33 (d, *J* = 8.2 Hz, 2H), 6.89 (d, *J* = 3.4 Hz, 1H), 6.49 (dd, *J* = 3.4, 1.8 Hz, 1H), 3.19 (s, 3H), 2.49 (s, 3H). ^13^C NMR (101 MHz, CDCl_3_) δ 161.9 (CO), 161.8 (CO), 152.6 (Cq), 144.7 (CH), 141.7 (Cq), 140.1 (Cq), 137.0 (Cq), 131.5 (Cq), 129.8 (2 × CH), 126.0 (2 × CH), 115.5 (Cq), 115.0 (CH), 112.0 (CH), 24.3 (NCH_3_), 21.4 (CH_3_). IR (ATR diamond, cm^−1^) ν: 2926, 2920, 1770, 1712, 1445, 1355, 1116, 897, 647, 564. HRMS: *m*/*z* [M + H]^+^ calculated for C_17_H_14_N_3_O_3_: 308.1029, found: 308.1028.

#### 3.2.24. 3-(FuraN-3-yl)-5-methyl-2-(*p*-tolyl)pyrrolo[3,4-*c*]pyrazole-4,6-(2*H*,5*H*)-dione (**26**)

In a microwave vial, 0.5–2 mL with a stir bar was charged: **15** (0.05 g, 0.18 mmol, 1.00 eq.), 3-furanylboronic acid (0.030 g, 0.27 mmol, 1.5 eq.), K_2_CO_3_ (0.075 g, 0.054 mmol, 3.0 eq.) and dry dioxane (3.0 mL). The mixture was degassed for 15 min, and then Pd(PPh_3_)_4_ (0.021 g, 0.018 mmol, 0.10 eq.) was added. The vial was sealed and then put in a microwave cavity. After 2 h of irradiation at 150 °C, the mixture was concentrated and purified by using flash chromatography with Petroleum Ether/EtOAc (1/9) as eluent to give **26** (0.039 g, 56%) as a white solid. M.p: 190–192 °C. ^1^H NMR (400 MHz, CDCl_3_) δ 7.61 (s, 1H), 7.35–7.38 (m, 5H), 7.54 (d, *J* = 1.1 Hz, 1H), 3.16 (s, 3H), 2.48 (s, 3H). ^13^C NMR (101 MHz, CDCl_3_) δ 162.5 (CO), 162.1 (CO), 152.6 (Cq), 143.8 (CH), 143.5 (CH), 140.8 (Cq), 136.7 (Cq), 134.5 (Cq), 130.3 (2 × CH), 126.4 (2 × CH), 116.1 (Cq), 113.6 (Cq), 109.6 (CH), 24.3 (NCH_3_), 21.4 (CH_3_). IR (ATR diamond, cm^−1^) ν: 2924, 2921, 1765, 1712, 1447, 1357, 1116, 977, 648, 594. HRMS: *m*/*z* [M + H]^+^ calculated for C_17_H_14_N_3_O_3_: 308.1029, found: 308.1032.

#### 3.2.25. 5-Methyl-3-(thiopheN-3-yl)-2-(*p*-tolyl)pyrrolo[3,4-*c*]pyrazole-4,6-(2*H*,5*H*)-dione (**27**)

In a microwave vial, 0.5–2 mL with a stir bar was charged: **15** (0.05 g, 0.18 mmol, 1.00 eq.), 3-thienylboronic acid (0.041 g, 0.27 mmol, 1.5 eq.), K_2_CO_3_ (0.075 g, 0.054 mmol, 3.0 eq.) and dry dioxane (3.0 mL). The mixture was degassed for 15 min, and then Pd(PPh_3_)_4_ (0.021 g, 0.018 mmol, 0.10 eq.) was added. The vial was sealed and then put in a microwave cavity. After 2 h of irradiation at 150 °C, the mixture was concentrated and purified by using flash chromatography with Petroleum Ether/EtOAc (1/9) as eluent to give **27** (0.026 g, 45%) as a white solid. M.p: 198–200 °C. ^1^H NMR (400 MHz, CDCl_3_) δ 7.63 (dd, *J* = 3.0, 1.3 Hz, 1H), 7.33–729 (m, 4H), 7.28 (dd, *J* = 5.2, 3.0 Hz, 1H), 7.21 (dd, *J* = 5.1, 1.3 Hz, 1H), 3.17 (s, 3H), 2.47 (s, 3H). ^13^C NMR (101 MHz, CDCl_3_) δ 162.7 (CO), 162.0 (CO), 152.5 (Cq), 140.5 (CH), 137.0 (CH), 136.9 (Cq), 130.3 (2 × CH), 128.3 (CH), 127.5 (CH), 127.3 (Cq), 126.3 (2 × CH), 126.3 (CH), 116.3 (Cq), 24.3 (NCH_3_), 21.4 (CH_3_). IR (ATR diamond, cm^−1^) ν: 3087, 2923, 2921, 1759, 1700, 1498, 1356, 1116, 971, 792, 509. HRMS: *m*/*z* [M + H]^+^ calculated for C_17_H_14_N_3_O_2_S: 324.0802, found: 324.0801.

#### 3.2.26. 5-(4-Methoxybenzyl)-2,3-di-p-tolylpyrrolo[3,4-*c*]pyrazole-4,6-(2*H*,5*H*)-dione (**29**)

In a microwave vial, 0.5–2 mL with a stir bar was charged: **16** (0.050 g, 0.114 mmol, 1.00 eq.), *p*-tolylphenylboronic acid (0.026 g, 0.171 mmol, 1.5 eq.), K_2_CO_3_ (0.047 g, 0.342 mmol, 3.0 eq.) and dry dioxane (3.0 mL). The mixture was degassed for 15 min, and then Pd(PPh_3_)_4_ (0.013 g, 0.011 mmol, 0.10 eq.) was added. The vial was sealed and then put in a microwave cavity. After 2 h of irradiation at 150 °C, the mixture was concentrated and purified by using flash chromatography with Petroleum Ether/EtOAc (5/5) as eluent to give **29** (0.035 g, 70%) as a white solid. M.p: 160–162 °C. ^1^H NMR (400 MHz, CDCl_3_) δ 7.39 (d, *J* = 7.8 Hz, 2H), 7.34 (d, *J* = 7.5 Hz, 2H), 7.22–7.26 (m, 4H), 7.14 (d, *J* = 7.5 Hz, 2H), 6.84 (d, *J* = 7.7 Hz, 2H), 4.75 (s, 2H), 3.77 (s, 3H), 2.41 (s, 3H), 2.34 (s, 3H). ^13^C NMR (63 MHz, CDCl_3_) δ 162.3 (CO), 161.7 (CO), 159.1 (Cq), 152.4 (Cq), 144.3 (Cq), 140.6 (Cq), 139.5 (Cq), 137.0 (Cq), 130.2 (2 × CH), 130.0 (2 × CH), 129.5 (2 × CH), 129.2 (2 × CH), 129.1 (Cq), 125.6 (2 × CH), 123.6 (Cq),117.2 (Cq), 114.0 (2 × CH), 55.3 (OCH_3_), 41.3 (CH_2_), 21.2 (CH_3_), 21.2 (CH_3_). IR (ATR diamond, cm^−1^) ν: 2922, 1764, 1707, 1513, 1313, 1150, 916, 845, 775. HRMS: *m*/*z* [M + H]^+^ calculated for C_27_H_24_N_3_O_3_: 438.1782, found: 438.1783.

#### 3.2.27. 5-Methyl-2-(4-nitrophenyl)-3-(*p*-tolyl)pyrrolo[3,4-*c*]pyrazole-4,6-(2*H*,5*H*)-dione (**30**)

In a microwave vial, 0.5–2 mL with a stir bar was charged: **17** (0.050 g, 0.16 mmol, 1.00 eq.), *p*-tolylphenylboronic acid (0.033 g, 0.245 mmol, 1.5 eq.), K_2_CO_3_ (0.067 g, 0.489 mmol, 3.0 eq.) and dry dioxane (3.0 mL). The mixture was degassed for 15 min and then Pd(PPh_3_)_4_ (0.022 g, 0.019 mmol, 0.10 eq.) was added. The vial was sealed and then put in a microwave cavity. After 2 h of irradiation at 150 °C, the mixture was concentrated and purified by using flash chromatography with Petroleum Ether/EtOAc (1/9) as eluent to give **30** (0.057 g, 84%) as a white solid. M.p: 222–224 °C. ^1^H NMR (400 MHz, CDCl_3_) δ 8.31 (d, *J* = 8.8 Hz, 2H), 7.61 (d, *J* = 8.8 Hz, 2H), 7.35 (d, *J* = 7.9 Hz, 2H), 7.26 (d, *J* = 7.9 Hz, 2H), 3.20 (s, 3H), 2.42 (s, 3H). ^13^C NMR (63 MHz, CDCl_3_) δ 161.8 (CO), 161.5 (CO), 153.6 (Cq), 147.4 (Cq), 144.1 (Cq), 141.8 (Cq), 141.6 (Cq), 130.0 (2 × CH), 129.2 (2 × CH), 126.1 (2 × CH), 124.8 (2 × CH), 122.9 (Cq), 118.3 (Cq), 24.5 (NCH_3_), 21.5 (CH_3_). IR (ATR diamond, cm^−1^) ν: 3077, 2919, 1764, 1715, 1447, 1343, 1104, 988, 754, 504. HRMS: *m*/*z* [M + H]^+^ calculated for C_19_H_15_N_3_O_4_: 363.1083, found: 363.1087.

#### 3.2.28. 5-Methyl-3-(phenylamino)-2-(*p*-tolyl)pyrrolo[3,4-*c*]pyrazole-4,6-(2*H*,5*H*)-dione (**31**)

A solution of the 3-chloro-5-methyl-2-(*p*-tolyl)pyrrolo[3,4-*c*]pyrazole-4,6-dione **15** (0.181 mmol, 1.0 eq.), cesium carbonate (0.553 mmol, 3.0 eq.) and the aniline (0.273 mmol, 1.5 eq.) in dry 1,4-dioxane (4 mL) was degassed by bubbling argon through the mixture for 15 min. Xantphos (0.1 eq.) and Pd_2_dba_3_ (0.05 eq.) were then added, and the mixture was heated at 100 °C for 1 h under microwave irradiation. The mixture was concentrated and purified by using flash chromatography on a silica gel column with Petroleum Ether/EtOAc (3/7) as eluent to give **31** (0.049 g, 83%) as a yellow solid. M.p: 214–216 °C. ^1^H NMR (400 MHz, CDCl_3_) δ 7.47 (d, *J* = 8.0 Hz, 2H), 7.43–7.34 (m, 4H), 7.20–7.16 (m, 3H), 6.37 (s, 1H), 3.08 (s, 3H), 2.46 (s, 3H). ^13^C NMR (101 MHz, CDCl_3_) δ 162.2 (CO), 161.0 (CO), 153.0 (Cq), 140.1 (Cq), 139.2 (Cq), 138.1 (Cq), 134.3 (Cq), 130.7 (2 × CH), 129.3 (2 × CH), 125.2 (2 × CH), 124.6 (CH), 120.0 (2 × CH), 100.7 (Cq), 24.2 (CH_3_), 21.3 (CH_3_). IR (ATR diamond, cm^−1^) ν: 3375, 1781, 1707, 1553, 1341, 1133, 966, 885, 747. HRMS: *m*/*z* [M + H]^+^ calculated for C_19_H_17_N_4_O_2_: 333.1342, found: 333.1346.

#### 3.2.29. 3-((4-Methoxyphenyl)amino)-5-methyl-2-(*p*-tolyl)pyrrolo[3,4-*c*]pyrazole-4,6-(2*H*,5*H*)-dione (**32**)

A solution of the 3-chloro-5-methyl-2-(*p*-tolyl)pyrrolo[3,4-*c*]pyrazole-4,6-dione **15** (0.181 mmol, 1.0 eq.), cesium carbonate (0.553 mmol, 3.0 eq.) and the *p*-anisidine (0.273 mmol, 1.5 eq.) in dry 1,4-dioxane (4 mL) was degassed by bubbling argon through the mixture for 15 min. Xantphos (0.1 eq.) and Pd_2_dba_3_ (0.05 eq.) were then added, and the mixture was heated at 100 °C for 1 h under microwave irradiation. The mixture was concentrated and purified by using flash chromatography on a silica gel column with Petroleum Ether/EtOAc (3/7) as eluent to give **32** (0.058 g, 88%) as a white solid. M.p: 204–206 °C. ^1^H NMR (400 MHz, CDCl_3_) δ 7.46 (d, *J* = 8.3 Hz, 2H), 7.37 (d, *J* = 8.3 Hz, 2H), 7.14 (d, *J* = 8.9 Hz, 2H), 6.92 (d, *J* = 8.9 Hz, 2H), 6.30 (s, 1H), 3.83 (s, 3H), 3.04 (s, 3H), 2.46 (s, 3H). ^13^C NMR (101 MHz, CDCl_3_) δ 162.3 (CO), 161.0 (CO), 157.4 (Cq), 152.0 (Cq), 141.0 (Cq), 139.9 (Cq), 134.4 (Cq), 131.1 (Cq), 130.7 (2 × CH), 125.1 (2 × CH), 123.3 (2 × CH), 114.4 (2 × CH), 99.4 (Cq), 55.5 (OCH_3_), 24.1 (CH_3_), 21.3 (CH_3_). IR (ATR diamond, cm^−1^) ν: 3323, 1760, 1704, 1506, 1361, 1231, 822, 7444. HRMS: *m*/*z* [M + H]^+^ calculated for C_20_H_19_N_4_O_3_: 363.1451, found: 363.1452.

#### 3.2.30. 3-((3-Methoxyphenyl)amino)-5-methyl-2-(*p*-tolyl)pyrrolo[3,4-*c*]pyrazole-4,6-(2*H*,5*H*)-dione (**33**)

A solution of the 3-chloro-5-methyl-2-(*p*-tolyl)pyrrolo[3,4-*c*]pyrazole-4,6-dione **15** (0.181 mmol, 1.0 eq.), cesium carbonate (0.553 mmol, 3.0 eq.) and the *m*-anisidine (0.273 mmol, 1.5 eq.) in dry 1,4-dioxane (4 mL) was degassed by bubbling argon through the mixture for 15 min. Xantphos (0.1 eq.) and Pd_2_dba_3_ (0.05 eq.) were then added, and the mixture was heated at 100 °C for 1 h under microwave irradiation. The mixture was concentrated and purified by using flash chromatography on a silica gel column with Petroleum Ether/EtOAc (3/7) as eluent to give **33** (0.055 g, 84%) as a white solid. M.p: 180–182 °C. ^1^H NMR (400 MHz, CDCl_3_) δ 7.46 (d, *J* = 7.8 Hz, 2H), 7.37 (d, *J* = 7.8 Hz, 2H), 7.26 (t, *J* = 9.8 Hz, 1H), 6.77 (s, 1H), 6.70 (t, *J* = 6.9 Hz, 2H), 6.39 (s, 1H), 3.83 (s, 3H), 3.09 (s, 3H), 2.46 (s, 3H). ^13^C NMR (101 MHz, CDCl_3_) δ 162.2 (CO), 161.0 (CO), 160.5 (Cq), 152.0 (Cq), 140.1 (Cq), 139.3 (Cq), 138.7 (Cq), 134.3 (Cq), 130.7 (2 × CH), 129.9 (CH), 125.2 (2 × CH), 111.7 (CH), 109.8 (CH), 105.5 (CH), 101.0 (Cq), 55.4 (OCH_3_), 24.2 (CH_3_), 21.3 (CH_3_). IR (ATR diamond, cm**^−1^**) ν: 3302, 1755, 1699, 1552, 1366, 1199, 966, 764, 743. HRMS: *m*/*z* [M + H]^+^ calculated for C_20_H_19_N_4_O_3_: 363.1451, found: 363.1453.

#### 3.2.31. 3-((2-Methoxyphenyl)amino)-5-methyl-2-(*p*-tolyl)pyrrolo[3,4-*c*]pyrazole-4,6-(2*H*,5*H*)-dione (**34**)

A solution of the 3-chloro-5-methyl-2-(*p*-tolyl)pyrrolo[3,4-*c*]pyrazole-4,6-dione **15** (0.181 mmol, 1.0 eq.), cesium carbonate (0.553 mmol, 3.0 eq.) and the *o*-anisidine (0.273 mmol, 1.5 eq.) in dry 1,4-dioxane (4 mL) was degassed by bubbling argon through the mixture for 15 min. Xantphos (0.1 eq.) and Pd_2_dba_3_ (0.05 eq.) were then added, and the mixture was heated at 100 °C for 1 h under microwave irradiation. The mixture was concentrated and purified by using flash chromatography on a silica gel column with Petroleum Ether/EtOAc (4/6) as eluent to give **34** (0.043 g, 65%) as a white solid. M.p: 218–220 °C. ^1^H NMR (400 MHz, CDCl_3_) δ 7.55 (dd, *J* = 7.3, 2.1 Hz, 1H), 7.50 (d, *J* = 8.3 Hz, 2H), 7.39 (d, *J* = 8.3 Hz, 2H), 7.15–7.05 (m, 2H), 6.90 (dd, *J* = 7.3, 2.1 Hz, 1H), 6.86 (s, 1H), 3.82 (s, 3H), 3.12 (s, 3H), 2.48 (s, 3H). ^13^C NMR (101 MHz, CDCl_3_) δ 162.2 (CO), 161.4 (CO), 152.0 (Cq), 148.7 (Cq), 139.8 (Cq), 138.6 (Cq), 134.5 (Cq), 130.6 (2 × CH), 127.7 (Cq), 125.0 (2 × CH), 123.9 (CH), 121.1 (CH), 118.7 (CH), 110.2 (CH), 100.7 (Cq), 55.7 (OCH_3_), 24.2 (CH_3_), 21.3 (CH_3_). IR (ATR diamond, cm^−1^) ν: 3390, 1757, 1707, 1550, 1357, 1196, 985, 748, 736. HRMS: *m*/*z* [M + H]^+^ calculated for C_20_H_19_N_4_O_3_: 363.1451, found: 363.1452.

#### 3.2.32. 5-Methyl-2-(*p*-tolyl)-3-((4-(trifluoromethyl)phenyl)amino)pyrrolo[3,4-*c*]pyrazole-4,6-(2*H*,5*H*)-dione (**35**)

A solution of the 3-chloro-5-methyl-2-(*p*-tolyl)pyrrolo[3,4-*c*]pyrazole-4,6-dione **15** (0.181 mmol, 1.0 eq.), cesium carbonate (0.553 mmol, 3.0 eq.) and the *o*-anisidine (0.273 mmol, 1.5 eq.) in dry 1,4-dioxane (4 mL) was degassed by bubbling argon through the mixture for 15 min. Xantphos (0.1 eq.) and Pd_2_dba_3_ (0.05 eq.) were then added, and the mixture was heated at 100 °C for 1 h under microwave irradiation. The mixture was concentrated and purified by using flash chromatography on a silica gel column with Petroleum Ether/EtOAc (4/6) as eluent to give **35** (0.030 g, 41%) as a blue solid. M.p: 190–192 °C. ^1^H NMR (400 MHz, CDCl_3_) δ 7.60 (d, *J* = 8.3 Hz, 2H), 7.41 (d, *J* = 7.9 Hz, 2H), 7.35 (d, *J* = 7.9 Hz, 2H), 7.18 (d, *J* = 8.3 Hz, 2H), 6.51 (s, 1H), 3.09 (s, 3H), 2.44 (s, 3H). ^13^C NMR (101 MHz, CDCl_3_) δ 161.9 (CO), 161.1 (CO), 151.9 (Cq), 141.20 (q, *J* = 1.1 Hz, Cq), 140.5 (Cq), 137.1 (Cq), 134.0 (Cq), 130.8 (2 × CH), 126.6 (q, *J* = 3.7 Hz, 2 × CH), 125.6 (q, *J* = 32.7 Hz, Cq), 125.2 (2 × CH), 123.9 (CH), 118.0 (2 × CH), 102.4 (Cq), 24.3 (CH_3_), 21.3 (CH_3_). IR (ATR diamond, cm^−1^) ν: 3309, 1763, 1713, 1541, 1323, 1108, 1506, 831. HRMS: *m*/*z* [M + H]^+^ calculated for C_20_H_16_F_3_N_4_O_2_: 401.1220, found: 441.1219.

#### 3.2.33. 5-(4-Methoxybenzyl)-3-(anilino)-2-(*p*-tolyl)pyrrolo[3,4-*c*]pyrazole-4,6-(2*H*,5*H*)-dione (**39**)

A solution of the **16** (0.113 mmol, 1.0 eq.), cesium carbonate (0.339 mmol, 3.0 eq.) and the aniline (0.226 mmol, 2.0 eq.) in dry 1,4-dioxane (4 mL) was degassed by bubbling argon through the mixture for 15 min. Xantphos (0.1 eq.) and Pd_2_dba_3_ (0.05 eq.) were then added, and the mixture was heated at 100 °C for 1 h under microwave irradiation. The mixture was concentrated and purified by using flash chromatography on a silica gel column with Petroleum Ether/EtOAc (3/7) as eluent to give **39** (0.034g, 68%) as a yellow solid. M.p: 234–236 °C. ^1^H NMR (400 MHz, DMSO-*d*_6_) δ 9.45 (s, 1H), 8.13 (d, *J* = 8.7 Hz, 2H), 7.93 (d, *J* = 8.6 Hz, 2H), 7.29 (t, *J* = 7.9 Hz, 2H), 7.19 (d, *J* = 8.7 Hz, 2H), 7.15 (d, *J* = 7.6 Hz, 2H), 7.03 (t, *J* = 7.3 Hz, 1H), 6.87 (d, *J* = 8.7 Hz, 2H), 4.57 (s, 2H), 3.71 (s, 3H), 3.30 (s, 3H). ^13^C NMR (101 MHz, DMSO-*d*_6_) δ 161.9 (CO), 160.2 (CO), 159.0 (Cq), 152.7 (Cq), 142.3 (Cq), 140.9 (Cq), 140.9 (Cq), 140.2 (Cq), 133.0 (Cq), 129.5 (2 x CH), 129.3 (2 x CH), 129.2 (2 × CH), 129.10, 126.0 (2 × CH), 123.2 (CH), 119.3 (2 × CH), 114.4 (2 × CH), 102.3 (Cq), 55.5 (OCH_3_), 43.9 (CH_3_), 40.9 (CH_2_). IR (ATR diamond, cm^−1^) ν: 3174, 1786, 1707, 1553, 1356, 1133, 971, 885, 733. HRMS: *m*/*z* [M + H]^+^ calculated for C_26_H_22_N_4_O_3_: 439.1745, found: 439.1747.

#### 3.2.34. 5-Methyl-2-(4-nitrophenyl)-3-(phenylamino)pyrrolo[3,4-*c*]pyrazole-4,6-(2*H*,5*H*)-dione (**40**)

A solution of the **17** (0.163 mmol, 1.0 eq.), cesium carbonate (0.326 mmol, 3.0 eq.) and the aniline (0.196mmol, 1.5 eq.) in dry 1,4-dioxane (4 mL) was degassed by bubbling argon through the mixture for 15 min. Xantphos (0.1 eq.) and Pd_2_dba_3_ (0.05 eq.) were then added, and the mixture was heated at 100 °C for 1 h under microwave irradiation. The mixture was concentrated and purified by using flash chromatography on a silica gel column with Petroleum Ether/EtOAc (3/7) as eluent to give **40** (0.030 g, 51%) as a yellow solid. M.p: 240–242 °C. ^1^H NMR (400 MHz, Acetone-*d*_6_) δ 8.56 (s, 1H), 8.43 (d, *J* = 8.6 Hz, 2H), 8.03 (d, *J* = 8.6 Hz, 2H), 7.31 (t, *J* = 7.7 Hz, 2H), 7.20 (d, *J* = 8.1 Hz, 2H), 7.07 (t, *J* = 7.4 Hz, 1H), 2.99 (s, 3H). ^13^C NMR (101 MHz, Acetone-*d*_6_) δ 161.6 (CO), 160.1 (CO) 153.3 (Cq), 147.2 (Cq), 143.3 (Cq), 139.9 (Cq), 139.8 (Cq), 128.9 (2 × CH), 125.7 (2 × CH), 124.9 (2 × CH), 123.1 (CH), 119.0 (2 × CH), 103.1 (Cq), 23.3 (CH_3_). IR (ATR diamond, cm^−1^) ν: 3375, 1781, 1707, 1553, 1341, 1133, 966, 885, 747. HRMS: *m*/*z* [M + H]^+^ calculated for C_18_H_14_N_5_O_4_: 364.1033, found: 364.1036.

## 4. Conclusions

In summary, we have described in this work a synthetic pathway for the preparation of an original pyrrolo[3,4-c]pyrazole-4,6-(2*H*,5*H*)-dione platform and have developed several arylations/amination at its C-3 position. First, a reactivity study of these derivatives with respect to Suzuki–Miyaura coupling reactions has shown that the reaction is compatible with various arylboronic acids. A strong influence of electronic effect and steric hindrance has also been shown. A study of the Buchwald–Hartwig cross-coupling in C-3 position was also performed. The scope was investigated and showed its limitation to aniline derivatives. Secondly, this work allows access to a novel class of substituted pyrrolo[3,4-c]pyrazole-4,6-(2*H*,5*H*)-diones, which will undoubtedly have a major impact on the further synthesis of new bioactive compounds that contain the rare pyrrolo[3,4-c]pyrazole scaffold as the central skeleton. Finally, efforts to achieve these objectives, and particularly to study the reactivity of the maleimide moiety involved in the bicyclic system, are currently in progress.

## Figures and Tables

**Table 1:** Optimization of Suzuki–Miyaura cross-coupling reaction.

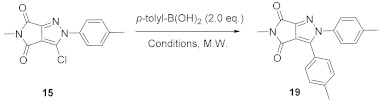						
Entry	Catalyst System (10 mol %)	Base (3.0 eq.)	T (°C)	Solvent	Time (h)	19, Yield (%) ^a^
1	PdCl_2_(PPh_3_)_2_	K_2_CO_3_	130	Dioxane	1.5	0
2	Pd(OAc)_2_/Xantphos (0.2 eq.)	K_2_CO_3_	130	Dioxane	1.5	20
3	Pd(PPh_3_)_4_	K_2_CO_3_	130	Dioxane	1.5	65
4	Pd(PPh_3_)_4_	K_2_CO_3_	150	Dioxane	1.5	80
5	Pd(PPh_3_)_4_	K_2_CO_3_	150	Dioxane	2.0	85
7	Pd(PPh_3_)_4_	Cs_2_CO_3_	150	Dioxane	2.0	79
6	Pd(PPh_3_)_4_	K_2_CO_3_	150	Toluene	2.0	0

^a^ Isolated yield.

**Table 2:** Synthesis of **19**–**30**.

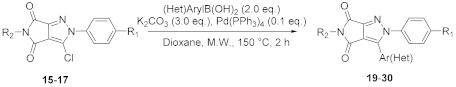					
Entry	Product	Yield (%) ^a^	Entry	Product	Yield (%) ^a^
1	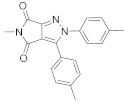 **19**	85%	7	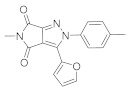 **25**	76%
2	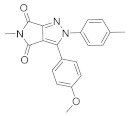 **20**	79%	8	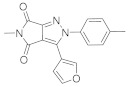 **26**	56%
3	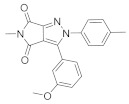 **21**	67%	9	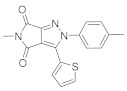 **27**	45%
4	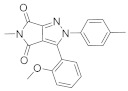 **22**	Traces	10	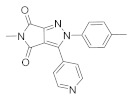 **28**	-
5	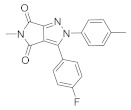 **23**	65%	11	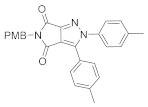 **29**	70%
6	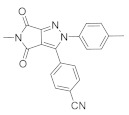 **24**	60%	12	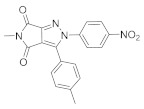 **30**	84%

^a^ Isolated yield.

**Table 3:** Optimization of the conditions for the formation of **31**.

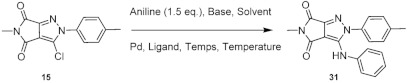							
Entry	Catalyst (10 mol %)	Ligand (20 mol %)	Base (3.0 eq.)	T (°C) M.W.	Solvent	Time (h)	31, Yield (%) ^a^
1	Pd(OAc)_2_	Xantphos	Cs_2_CO_3_	130	Dioxane	0.5	8
2	Pd_2_dba_3_	Xantphos	Cs_2_CO_3_	130	Dioxane	0.5	56
3	Pd_2_dba_3_	Xantphos	Cs_2_CO_3_	130	Dioxane	1	50
4	Pd_2_dba_3_	Xantphos	Cs_2_CO_3_	100	Dioxane	1	83
5	Pd_2_dba_3_	Xantphos	K_2_CO_3_	100	Dioxane	1	76
6	Pd_2_dba_3_	Xantphos	Cs_2_CO_3_	100	Toluene	1	N.D. ^b^
7	-	Xantphos	K_2_CO_3_	100	Dioxane	1	0

^a^ Isolated yield. ^b^ Not detected.

**Table 4:** Synthesis of **31**–**40**.

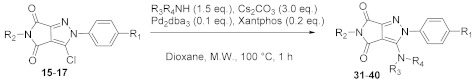					
Entry	Product	Yield (%) ^a^	Entry	Product	Yield (%) ^a^
1	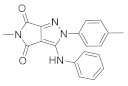 **31**	83%	6	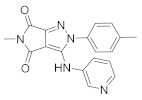 **36**	-
2	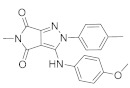 **32**	88%	7	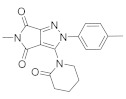 **37**	-
3	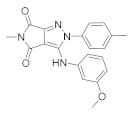 **33**	84%	8	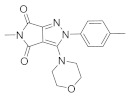 **38**	-
4	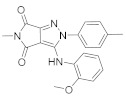 **34**	65%	9	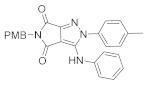 **39**	68%
5	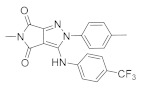 **35**	41%	10	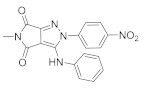 **40**	51%

^a^ Isolated yield.

## Data Availability

Data will be available upon request to the corresponding author.

## Supplementary Materials

The following supporting information can be downloaded at: https://www.mdpi.com/article/10.3390/molecules28155811/s1, Figures S1–S34: ^1^H and ^13^C NMR of all synthesized compounds.
