# Supplementary material for: Access and Modulation of Substituted Pyrrolo[3,4-c]pyrazole-4,6-(2H,5H)-diones
Source: Molecules. 2023 Aug 1;28(15):5811. doi: 10.3390/molecules28155811 (PMC10421423; doi:10.3390/molecules28155811)

Access and Modulation of Substituted Pyrrolo[3,4-*c*]pyrazole-4,6-(2*H*,5*H*)-diones

Abdelaziz Ejjoummany [1,2], Jonathan Elie [1], Ahmed El Hakmaoui [2], Mohamed Akssira [2], Sylvain Routier [1,*] and Frédéric Buron [1,*]

[1] Institut de Chimie Organique et Analytique, Université d'Orléans, UMR CNRS 7311, BP 6759, CEDEX 2, F-45067 Orléans, France
[2] Faculté des Sciences et Technique, Université Hassan II-Casablanca, BP 146, Mohammedia 28800, Morocco
* Correspondence: sylvain.routier@univ-orleans.fr (S.R.); frederic.buron@univ-orleans.fr (F.B.)

Figure S1: Ethyl 5-hydroxy-1-(p-tolyl)-1*H*-pyrazole-3-carboxylate (1)

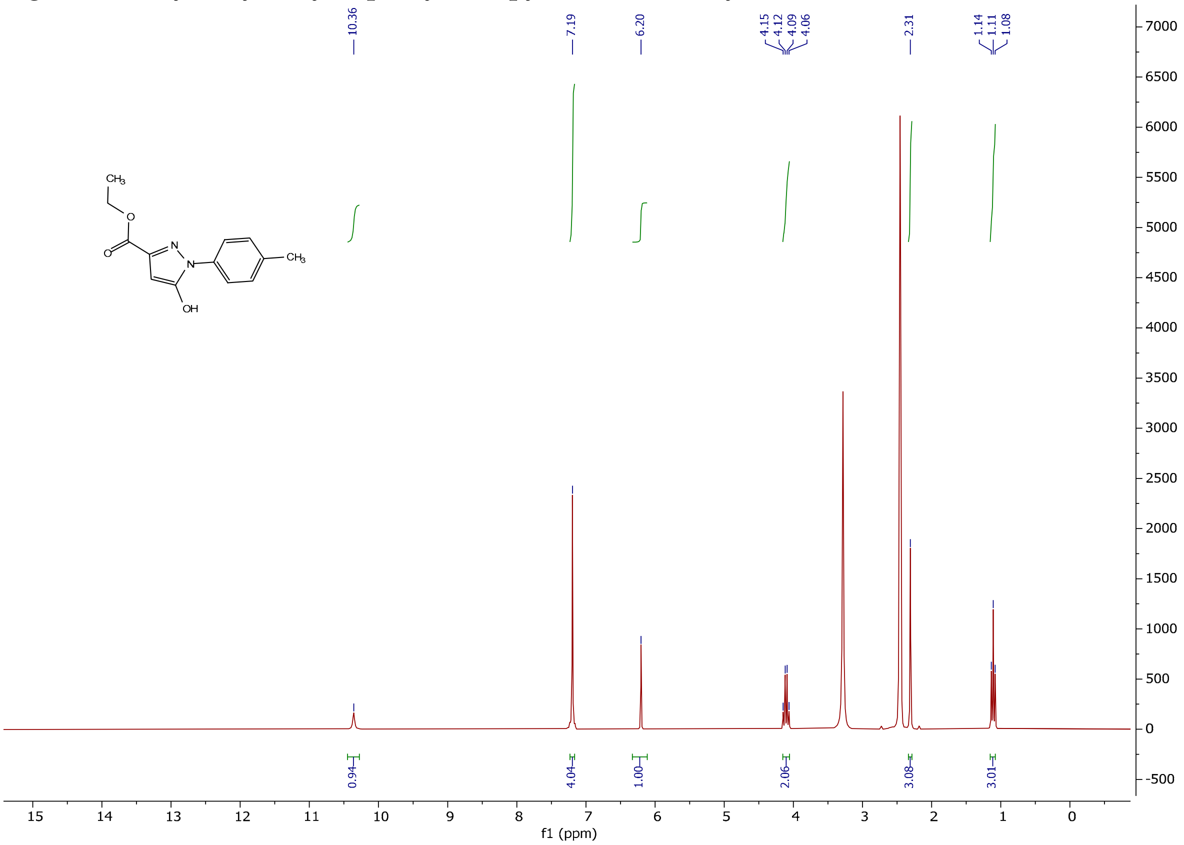

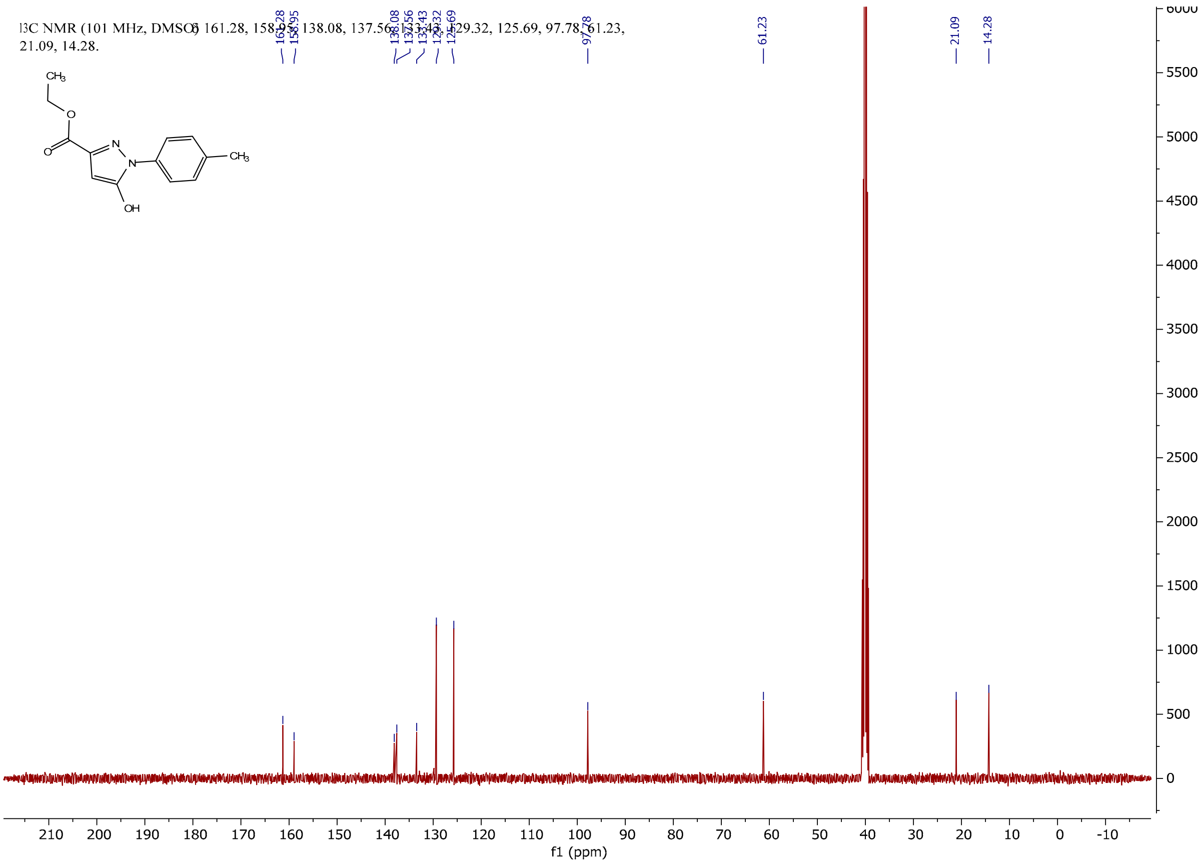

Figure S2: Ethyl 5-hydroxy-1-(4-nitrophenyl)-1*H*-pyrazole-3-carboxylate (2)

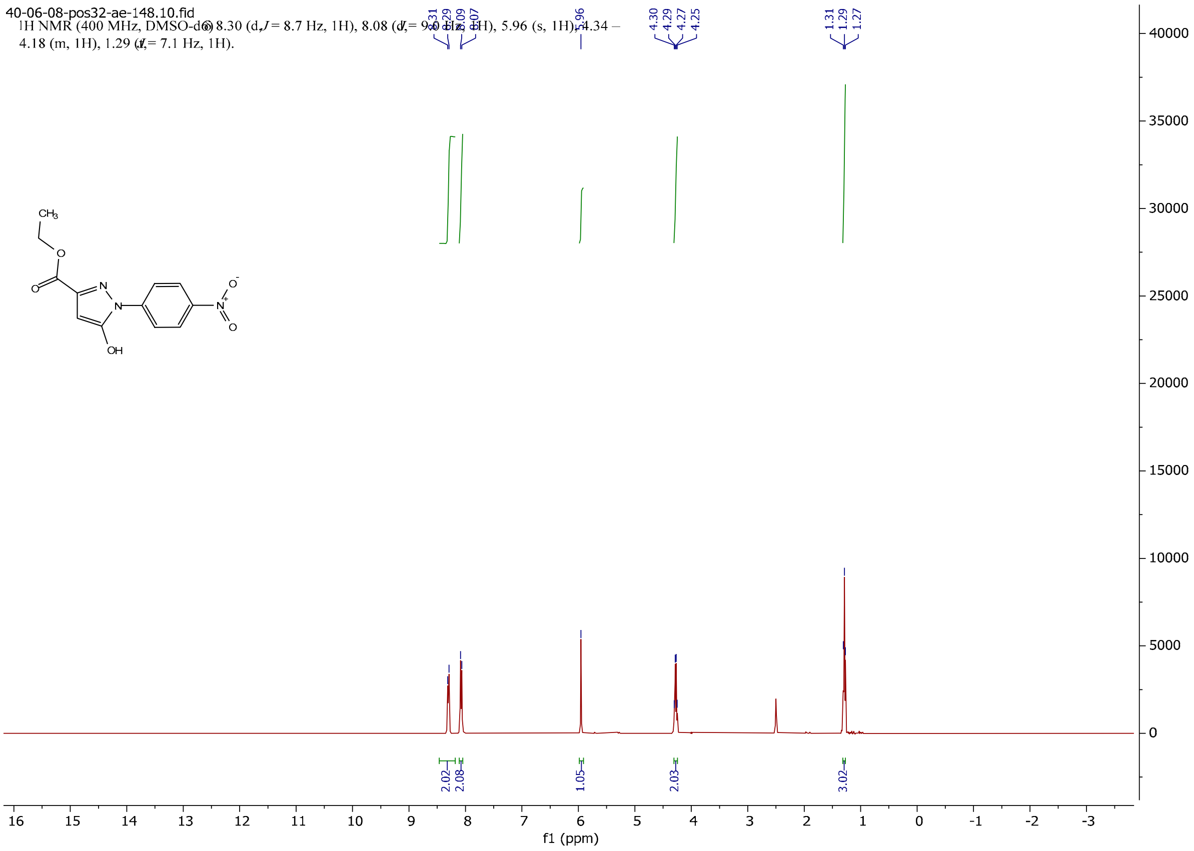

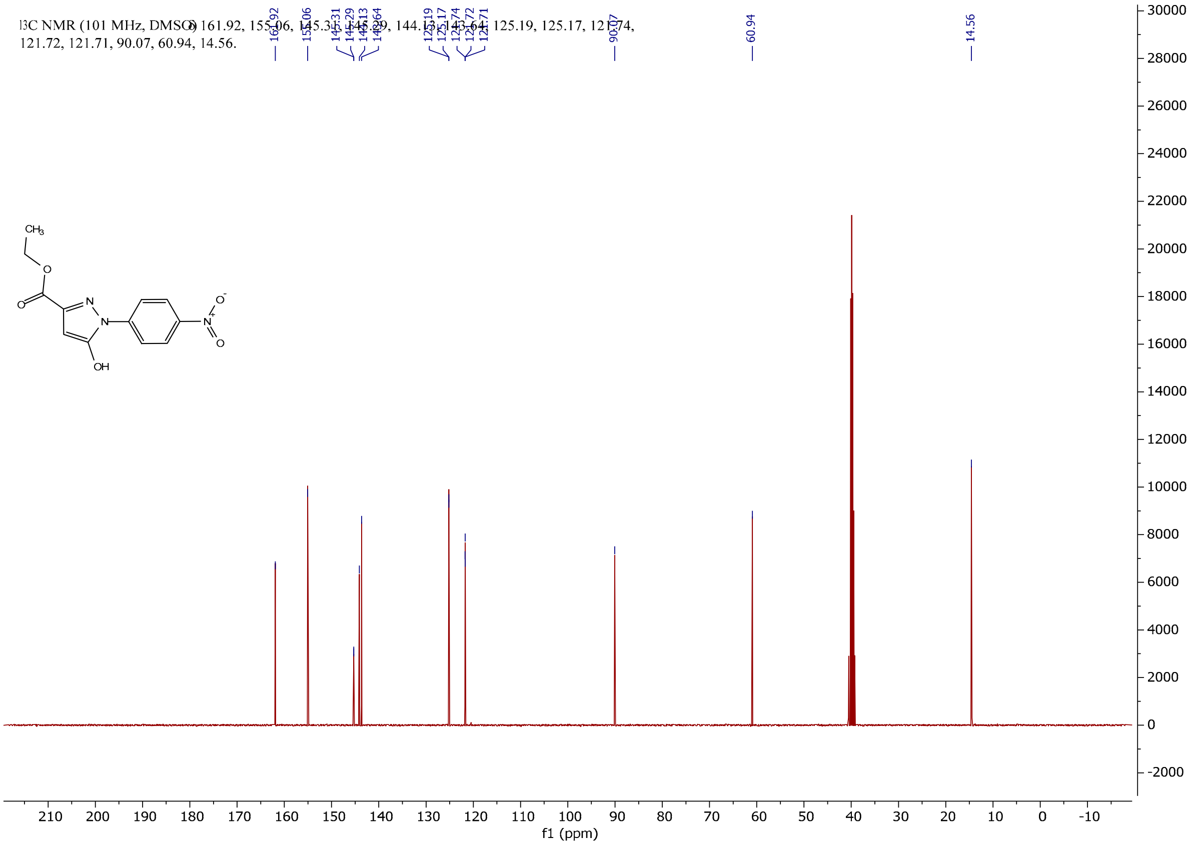

Figure S3: Ethyl 5-chloro-4-formyl-1-(p-tolyl)-1*H*-pyrazole-3-carboxylate (3)

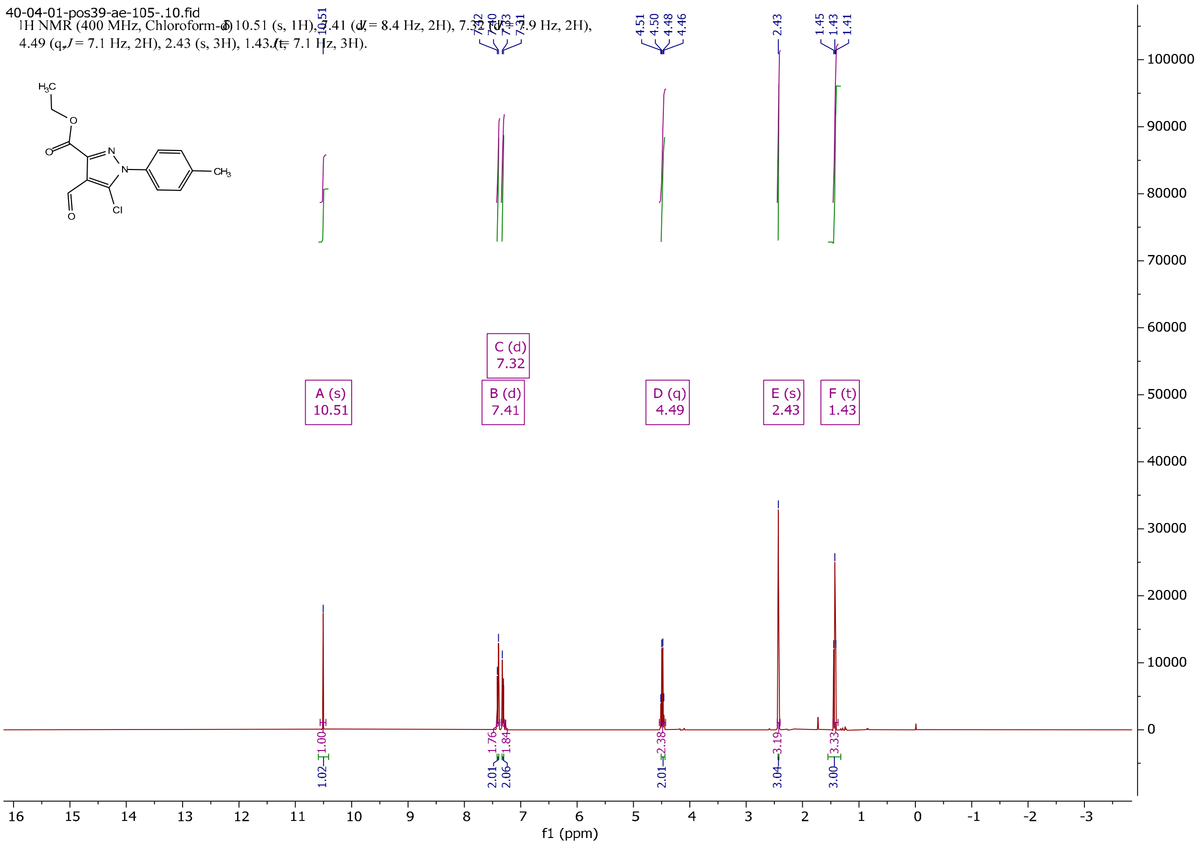

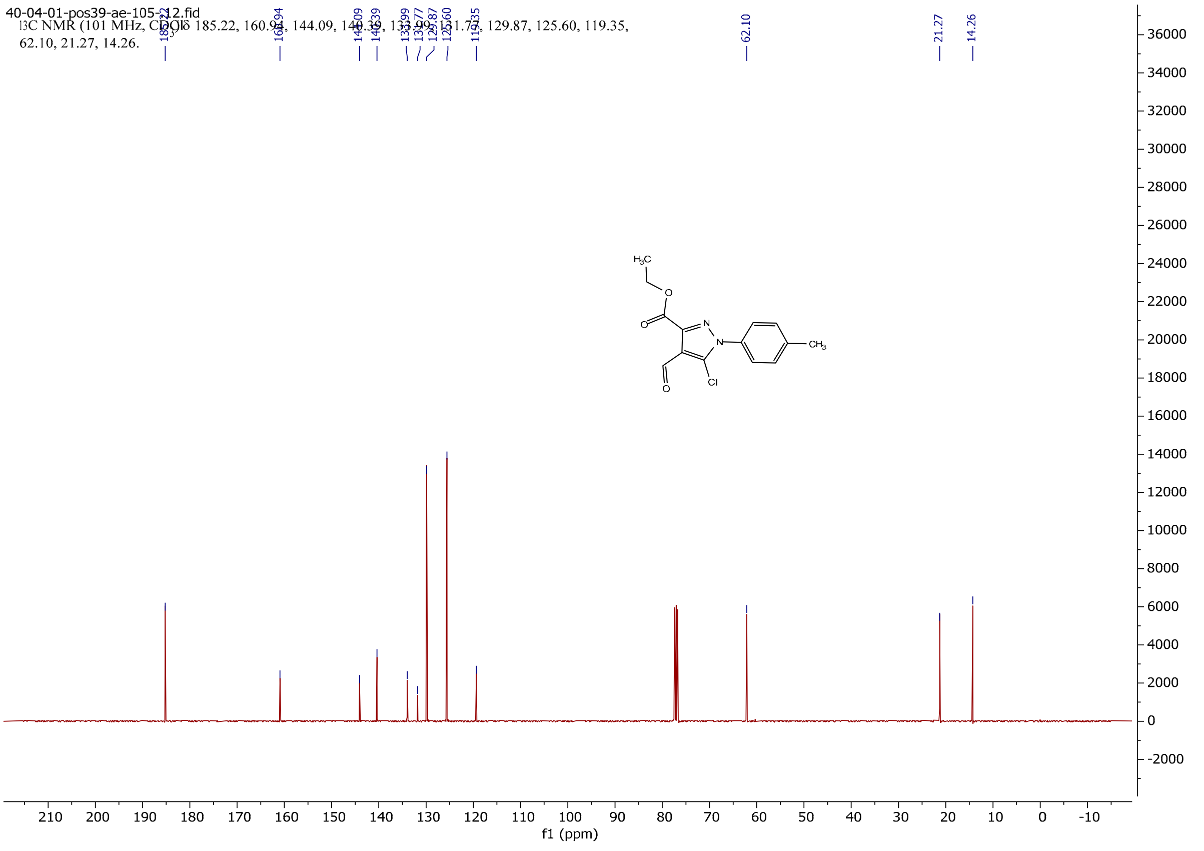

Figure S4: Ethyl 5-chloro-4-formyl-1-(4-nitrophenyl)-1*H*-pyrazole-3-carboxylate) (4)

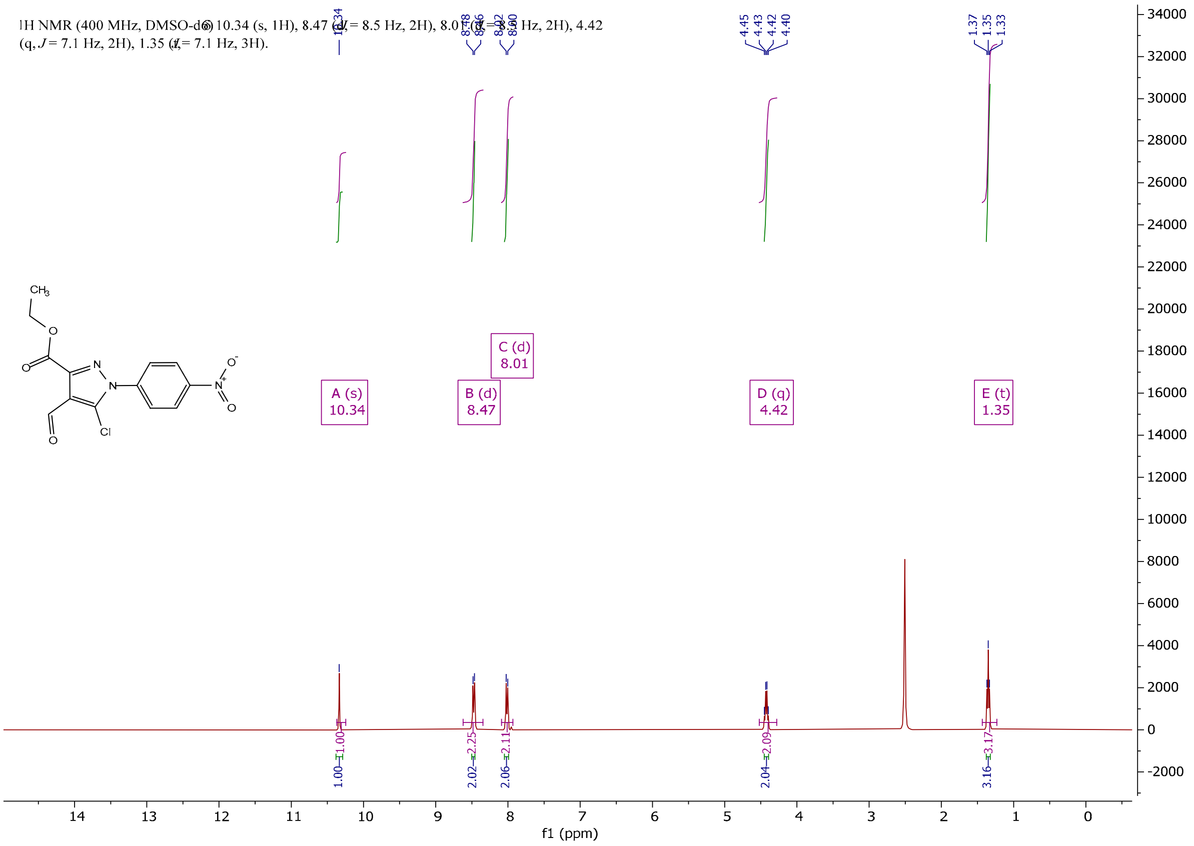

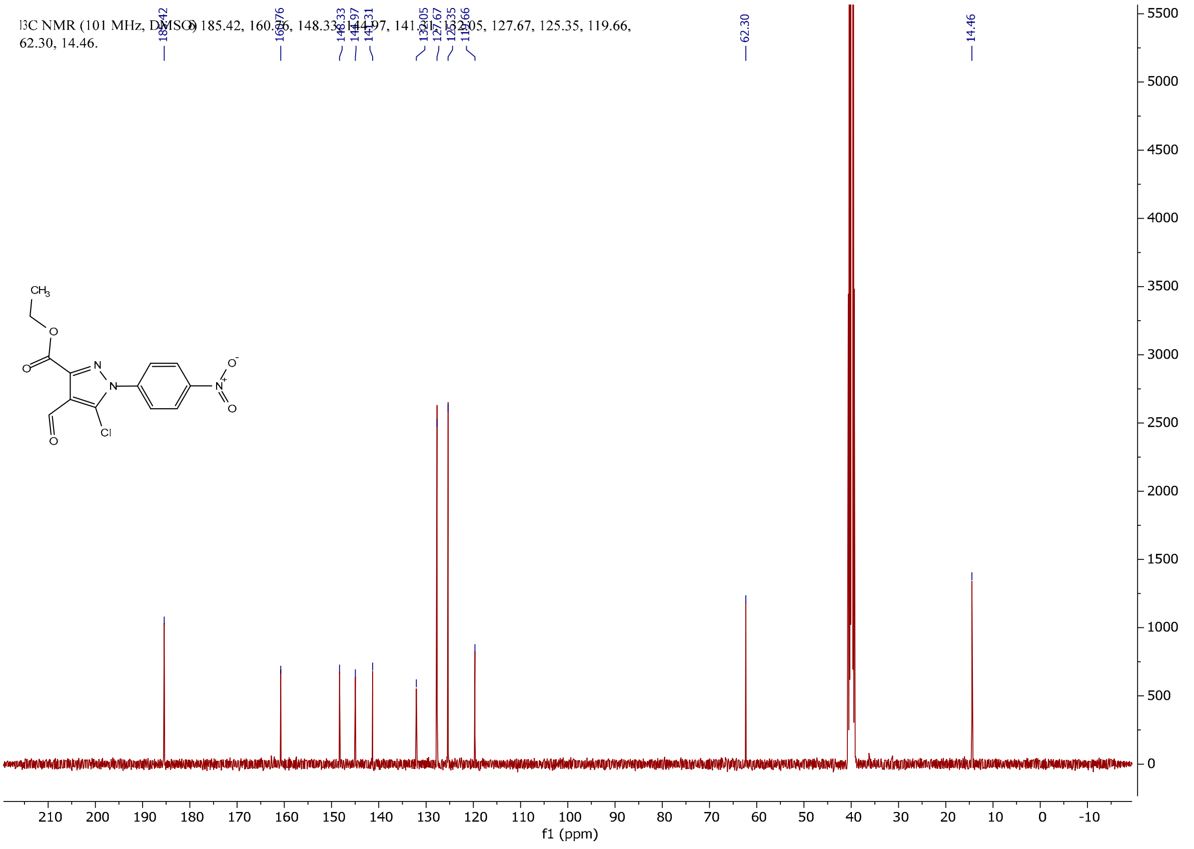

Figure S5: 5-Chloro-3-(ethoxycarbonyl)-1-(p-tolyl)-1*H*-pyrazole-4-carboxylic acid (5)

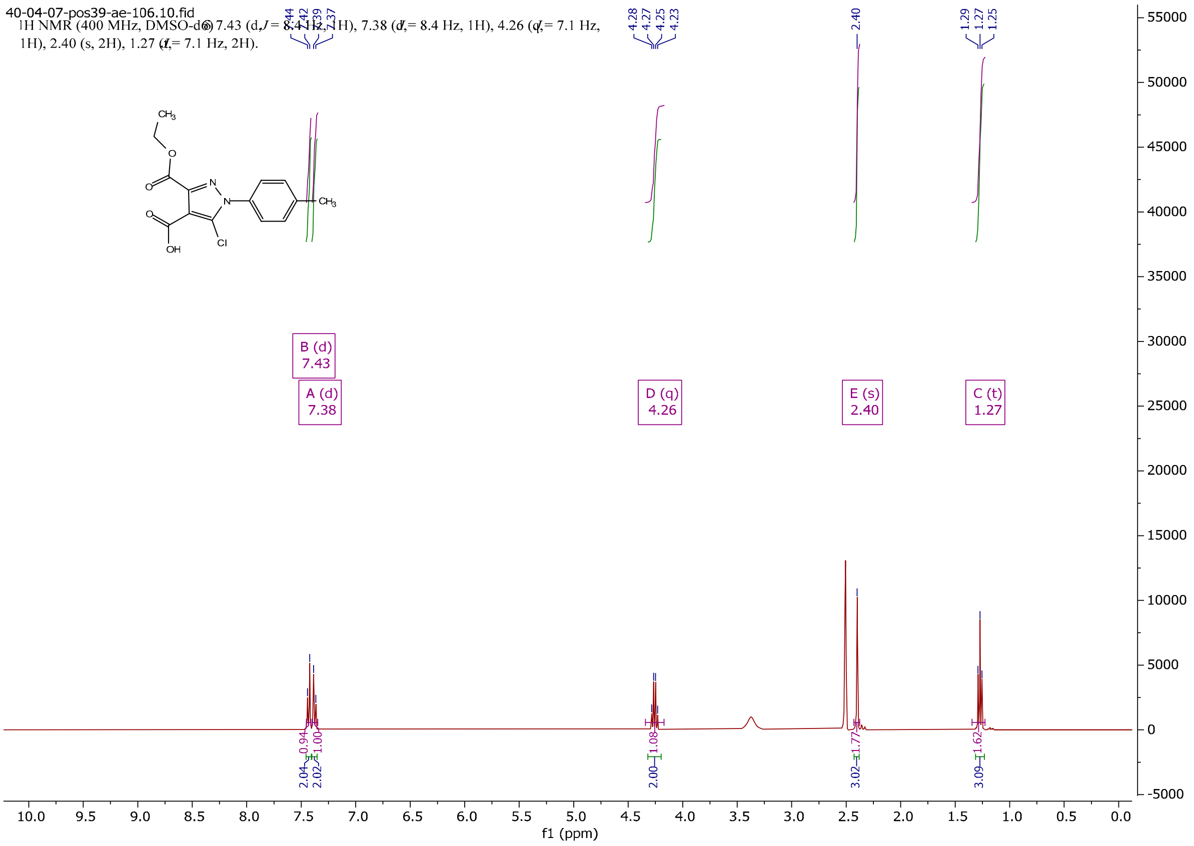

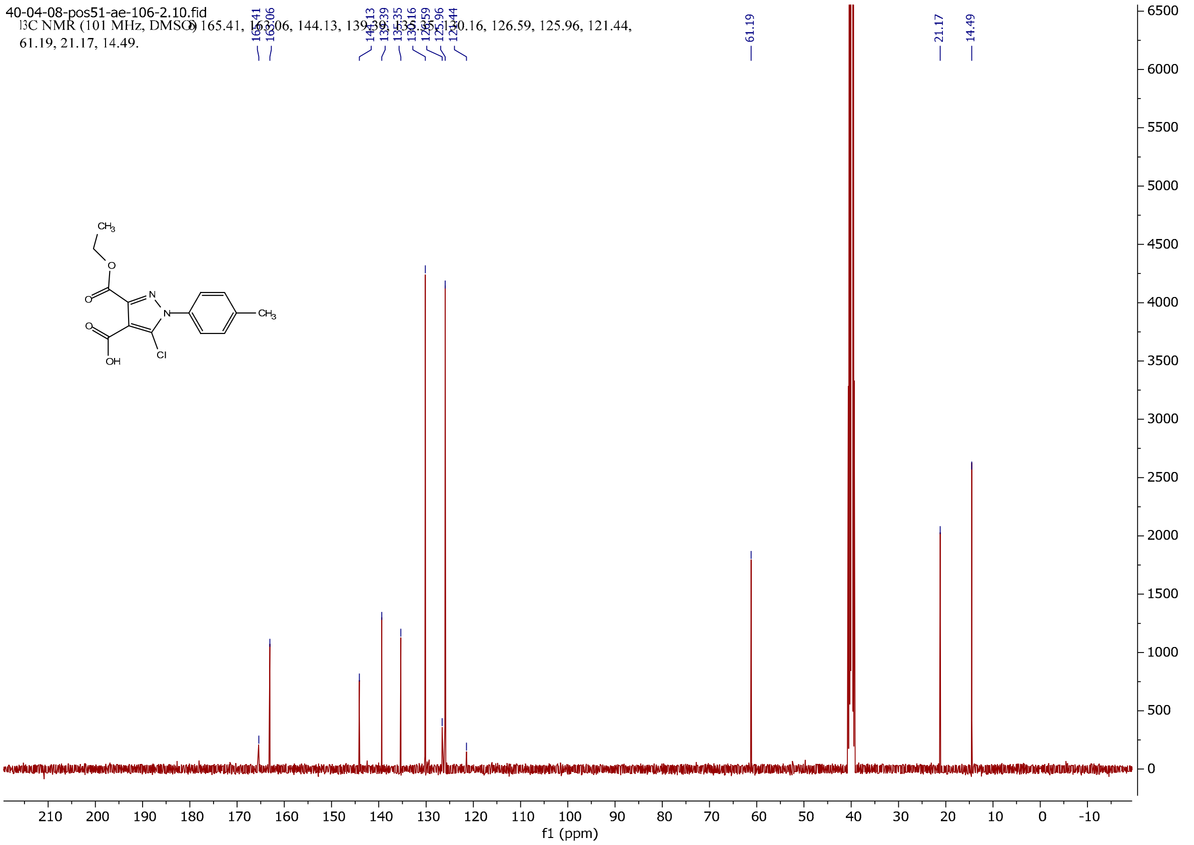

Figure S6: 5-Chloro-3-(ethoxycarbonyl)-1-(4-nitrophenyl)-1*H*-pyrazole-4-carboxylic acid (6)

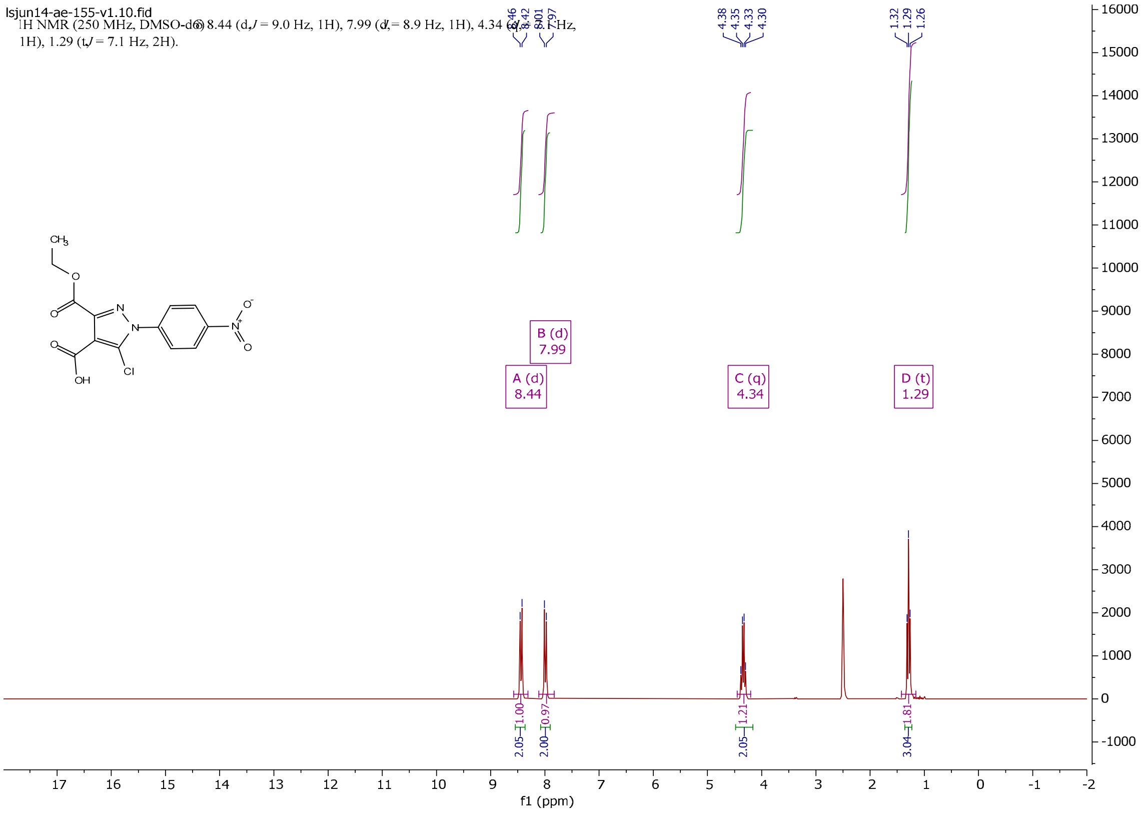

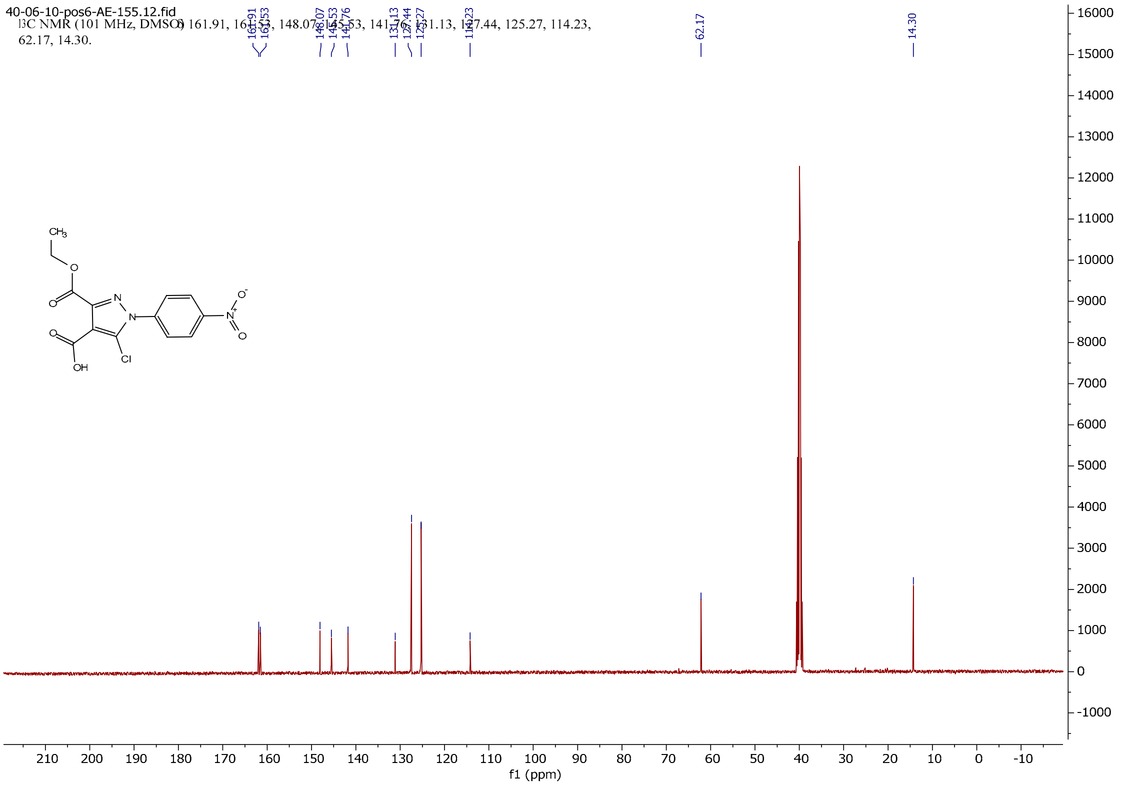

Figure S7: Ethyl 5-chloro-4-(methylcarbamoyl)-1-(p-tolyl)-1*H*-pyrazole-3-carboxylate (7)

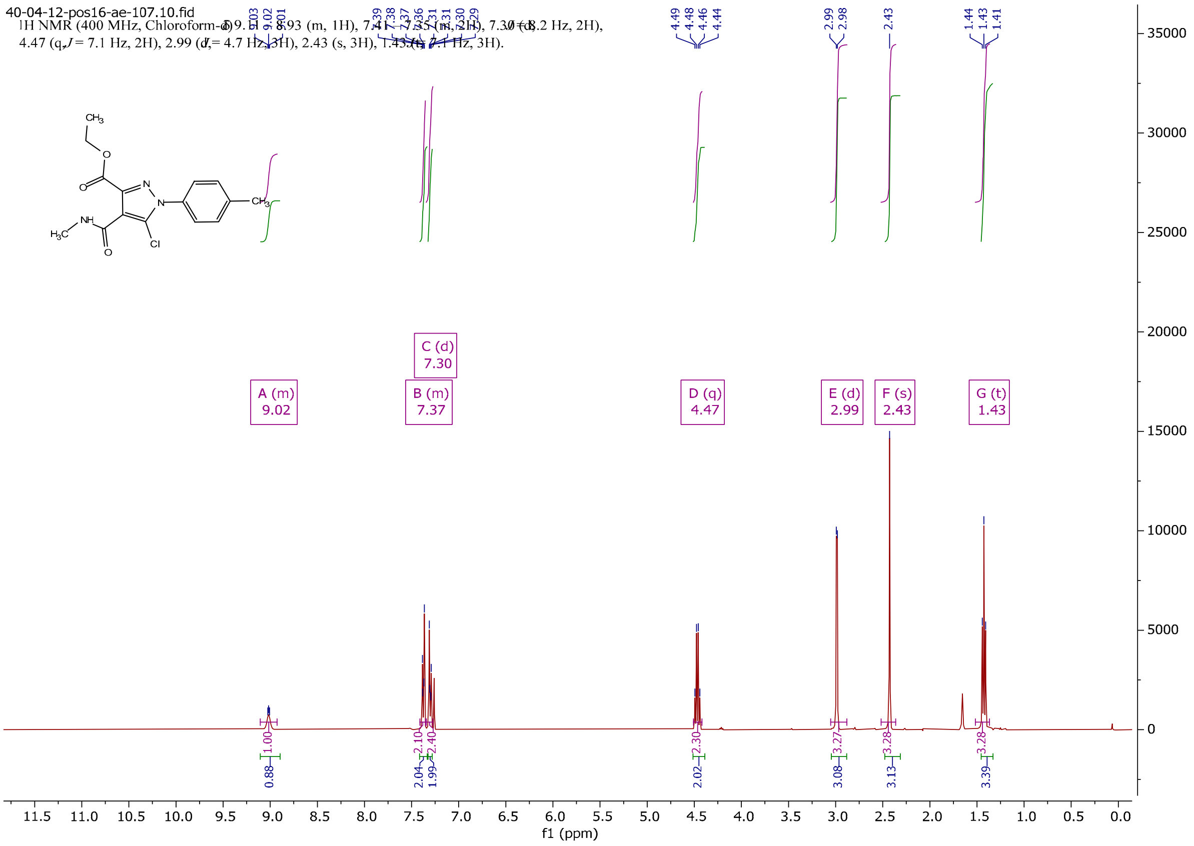

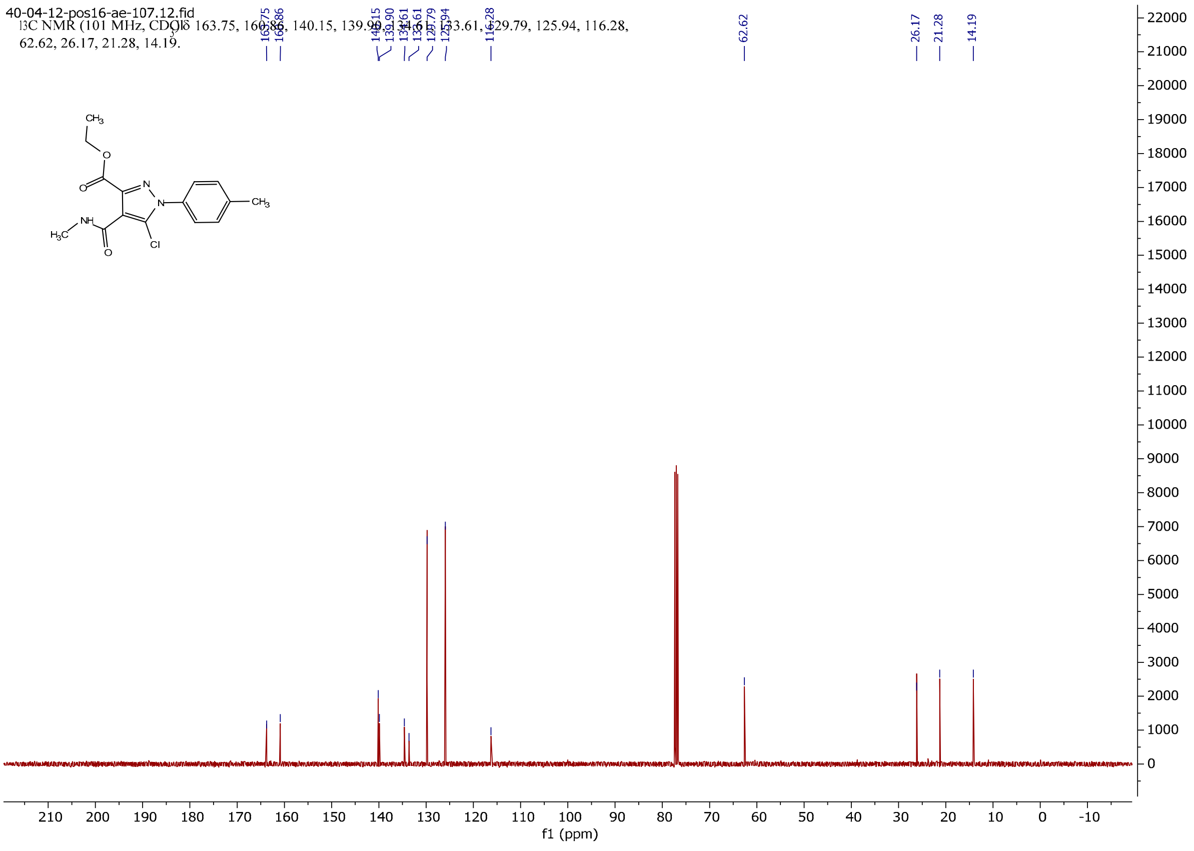

Figure S8: Ethyl 5-chloro-4-[(4-methoxyphenyl)methylcarbamoyl]-1-(p-tolyl)-1*H*-pyrazole-3-carboxylate (8)

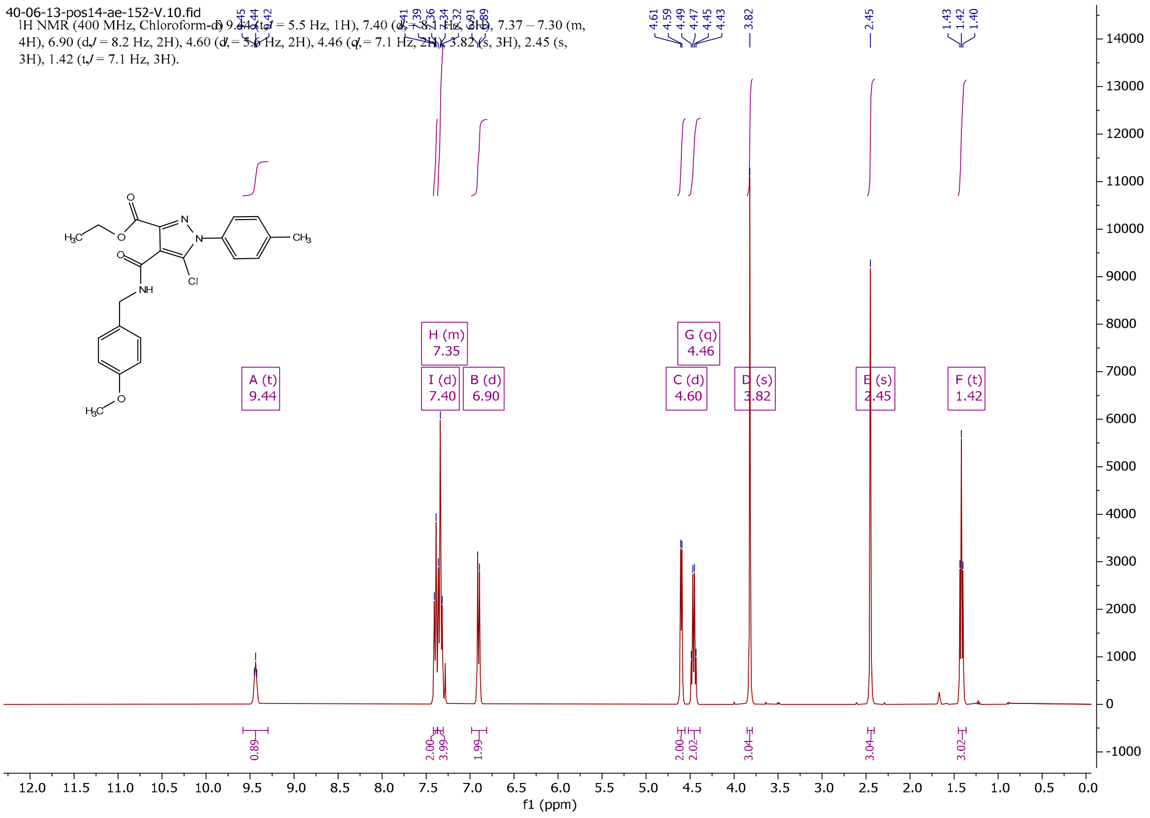

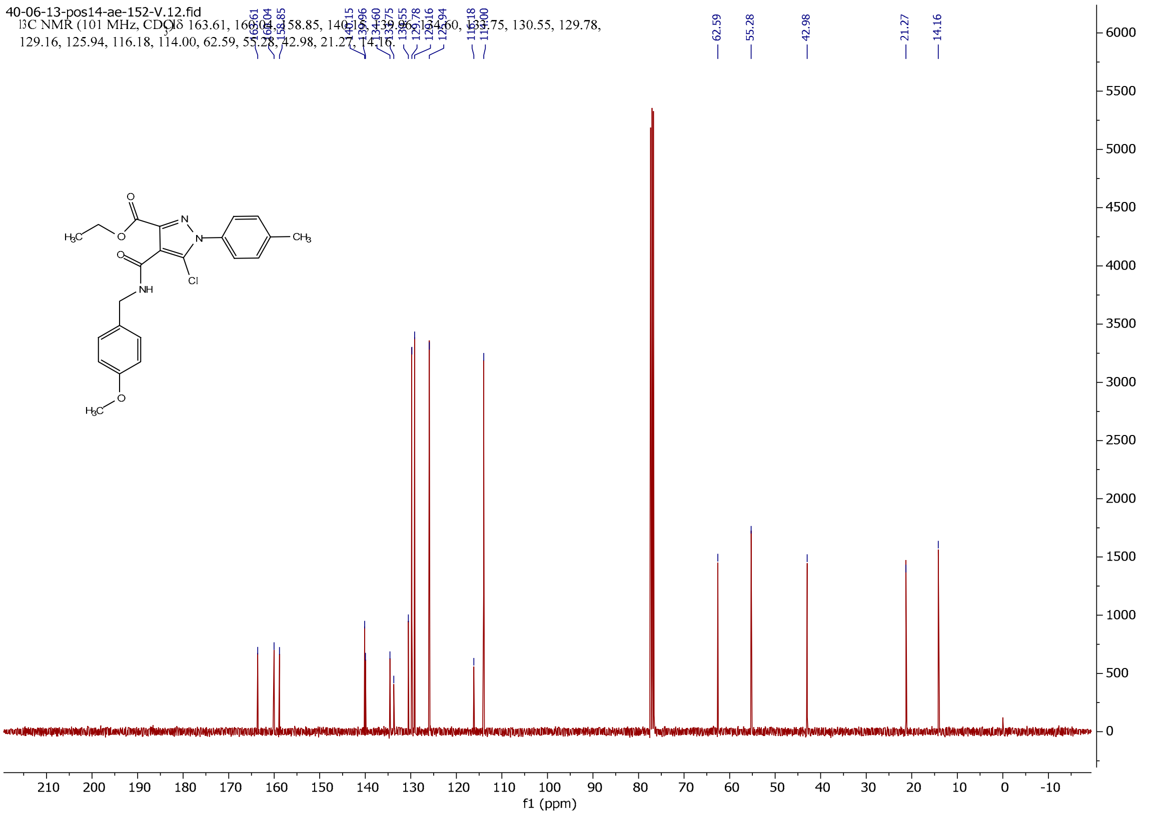

Figure S9: Ethyl 5-chloro-4-(methylcarbamoyl)-1-(4-nitrophenyl)-1*H*-pyrazole-3-carboxylate (9)

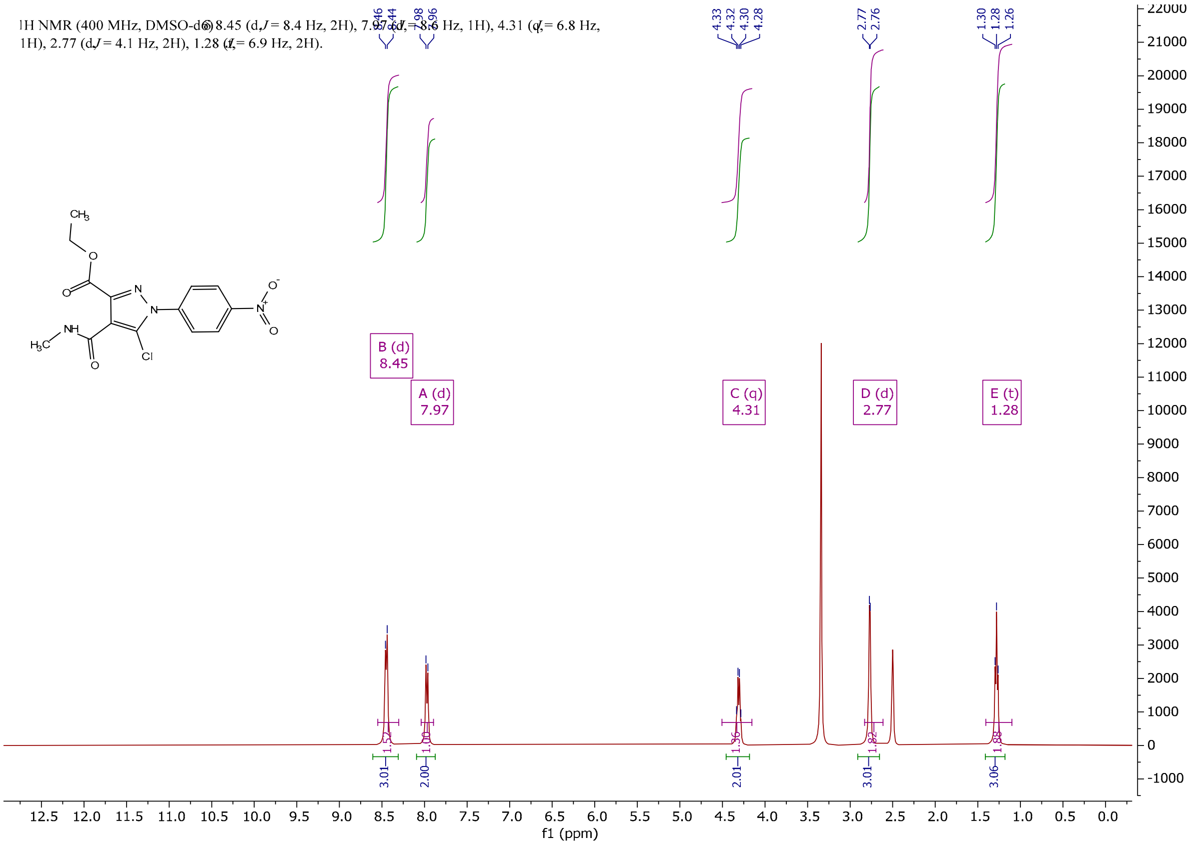

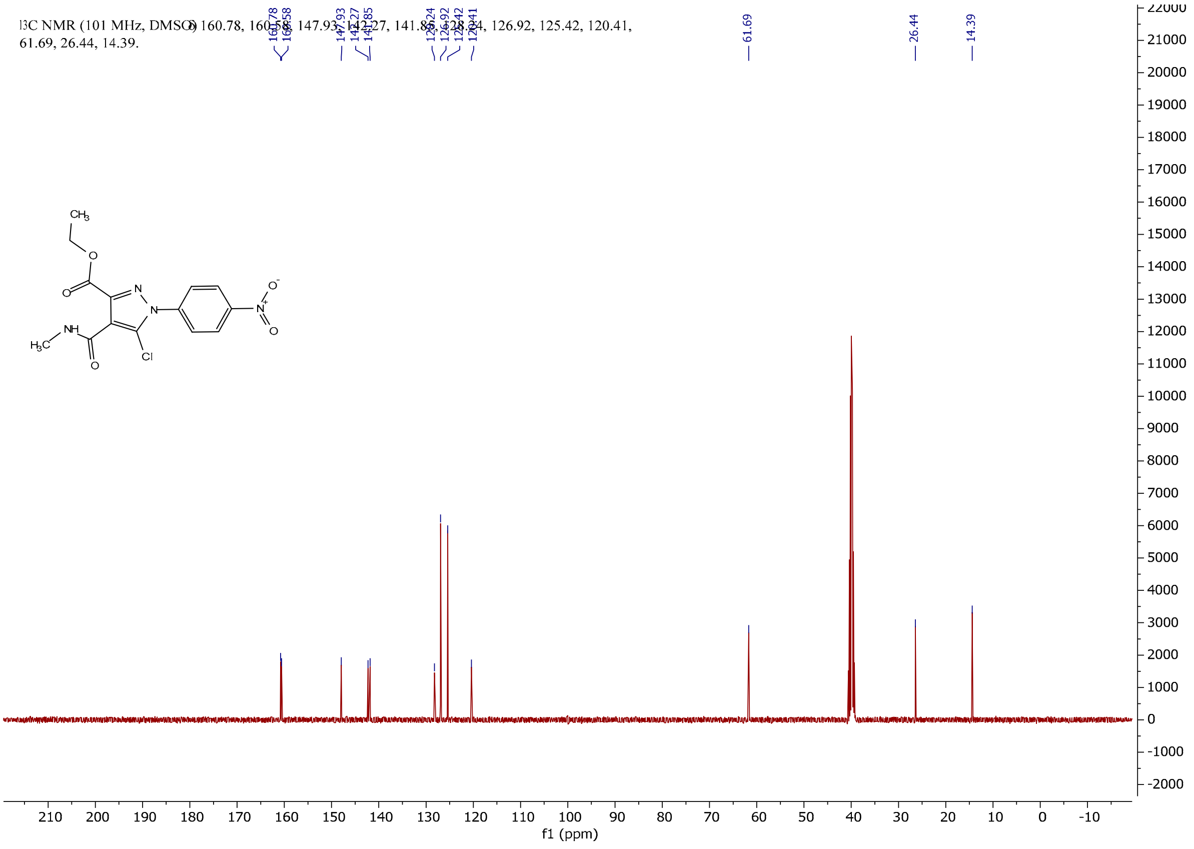

Figure S10: Ethyl 5-chloro-4-(phenylcarbamoyl)-1-(p-tolyl)-1*H*-pyrazole-3-carboxylate (10)

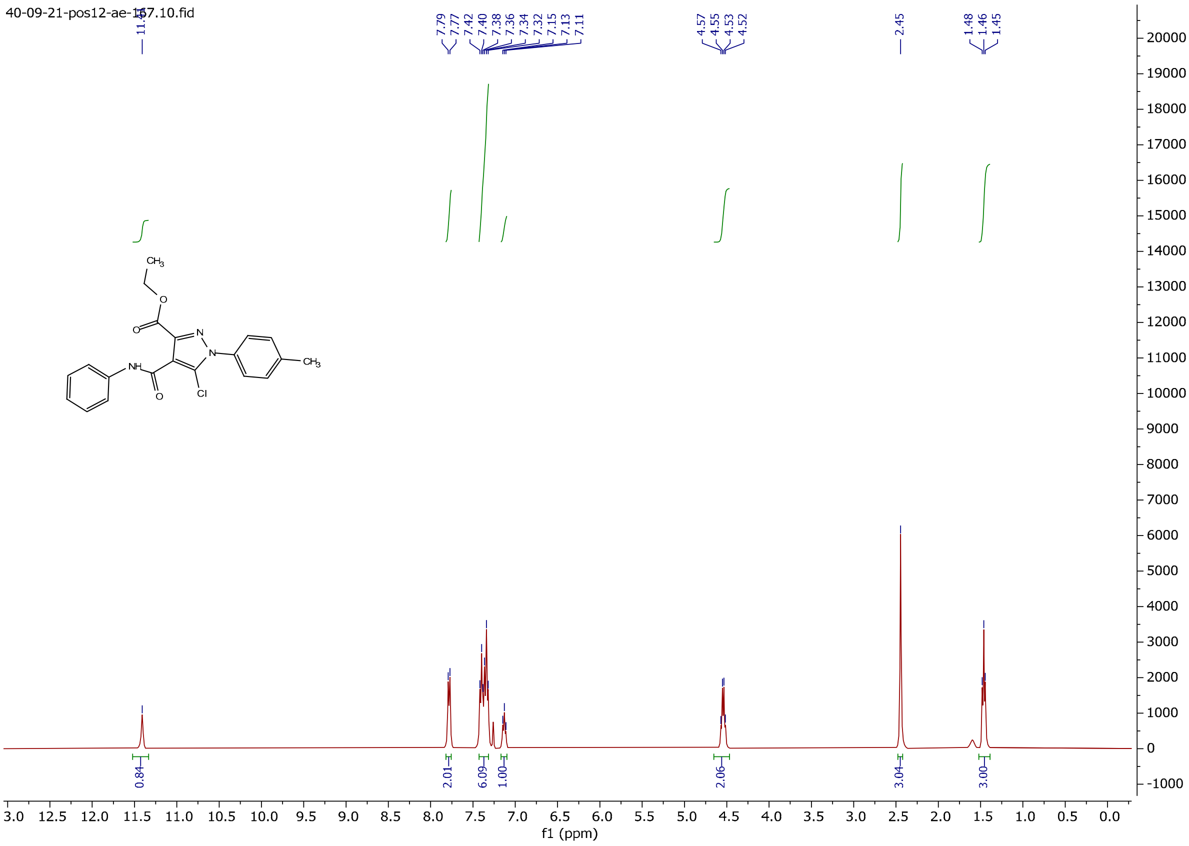

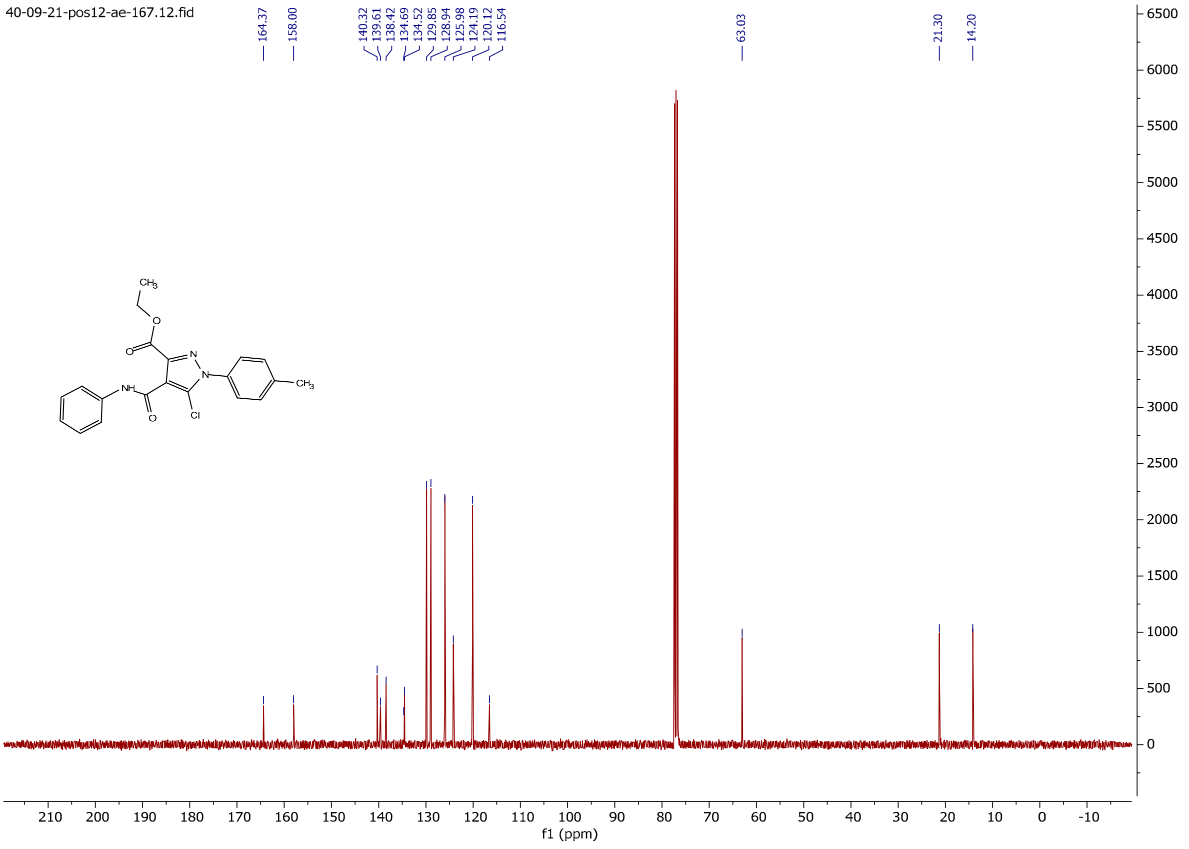

Figure S11: 5-Chloro-4-(methylcarbamoyl)-1-(p-tolyl)-1*H*-pyrazole-3-carboxylic acid (11)

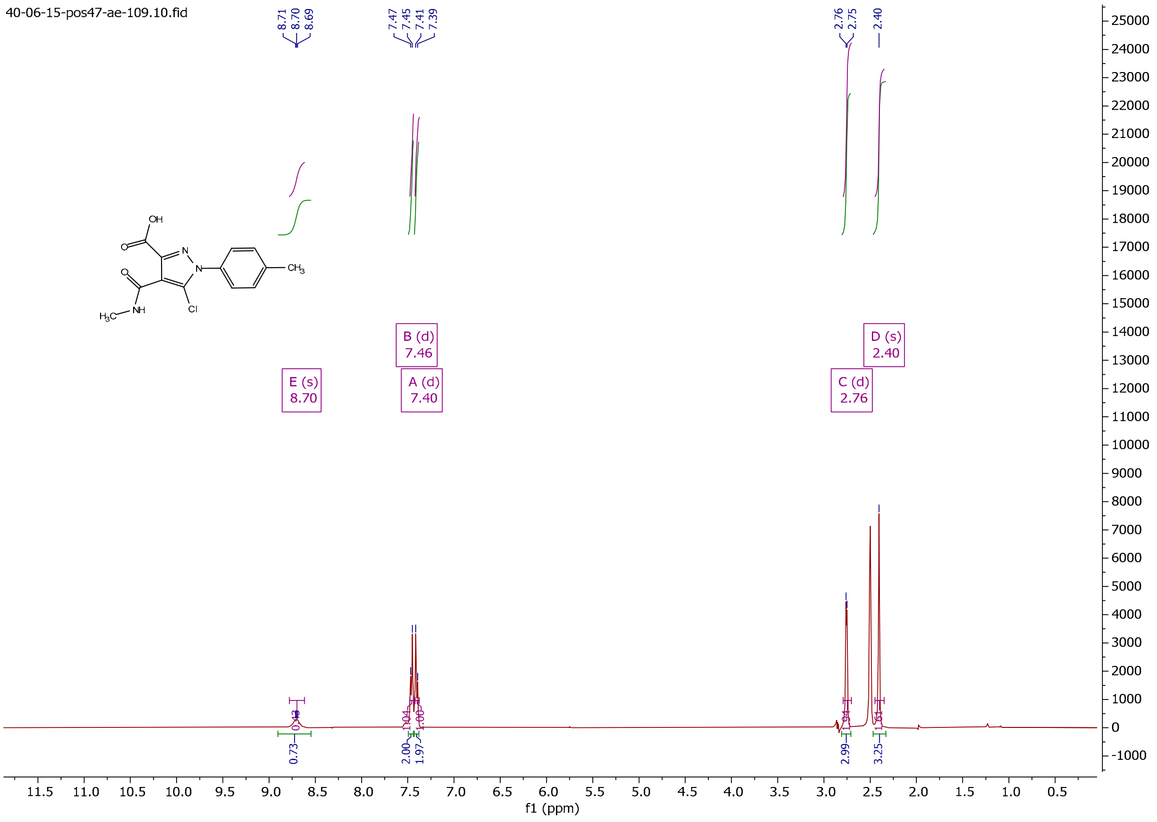

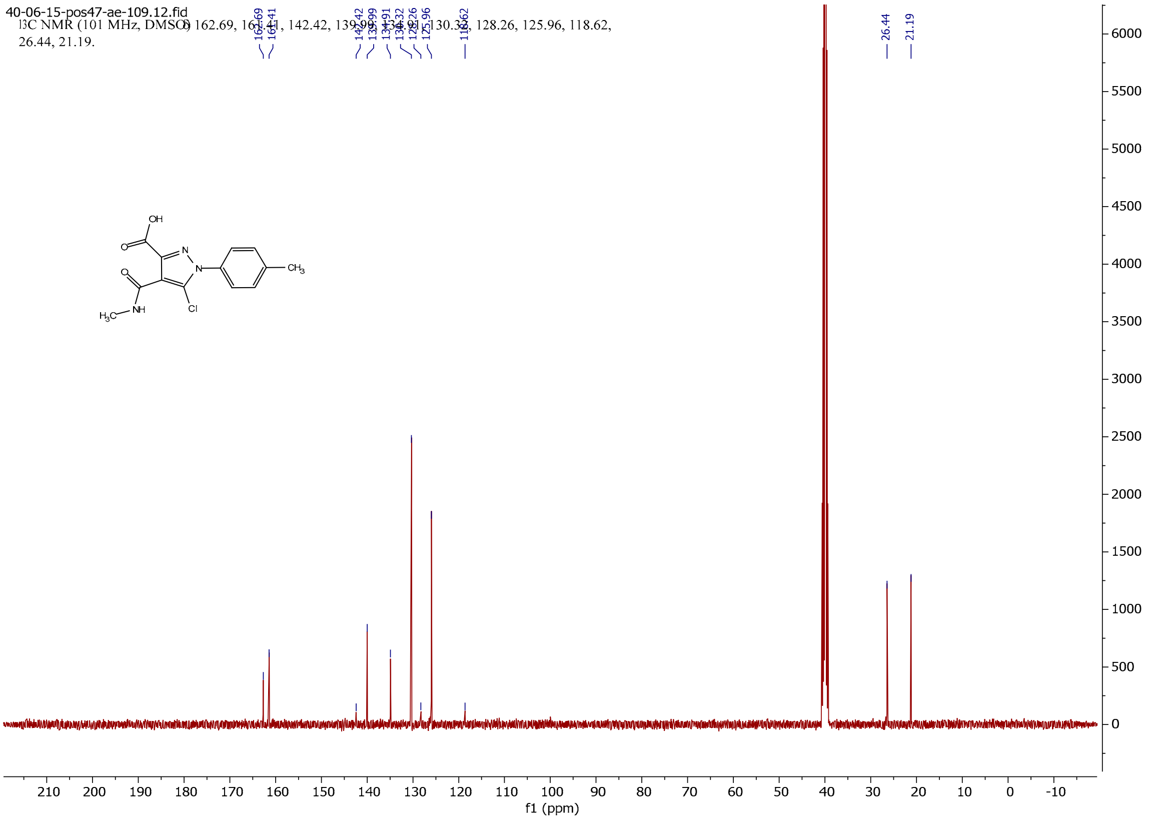

Figure S12: 5-Chloro-4-((4-methoxybenzyl)carbamoyl)-1-(p-tolyl)-1*H*-pyrazole-3-carboxylic acid (12)

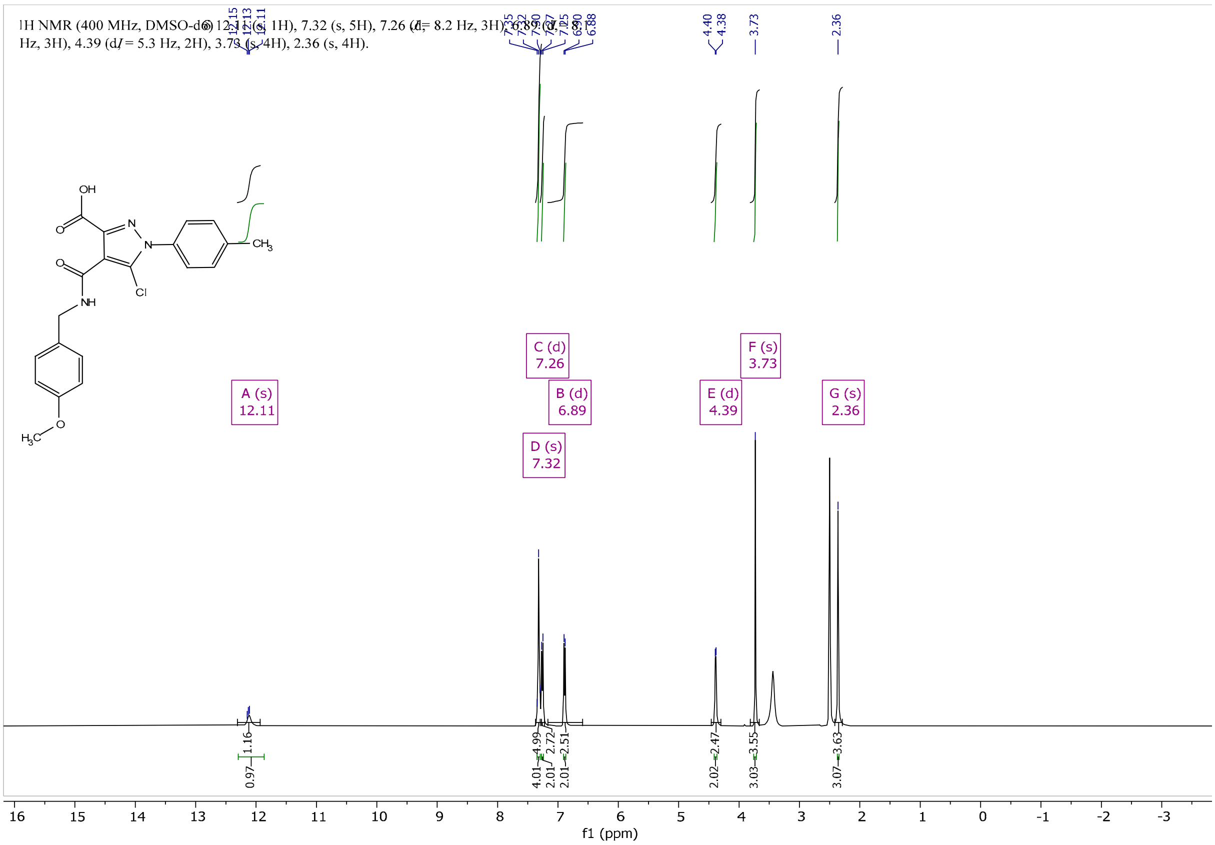

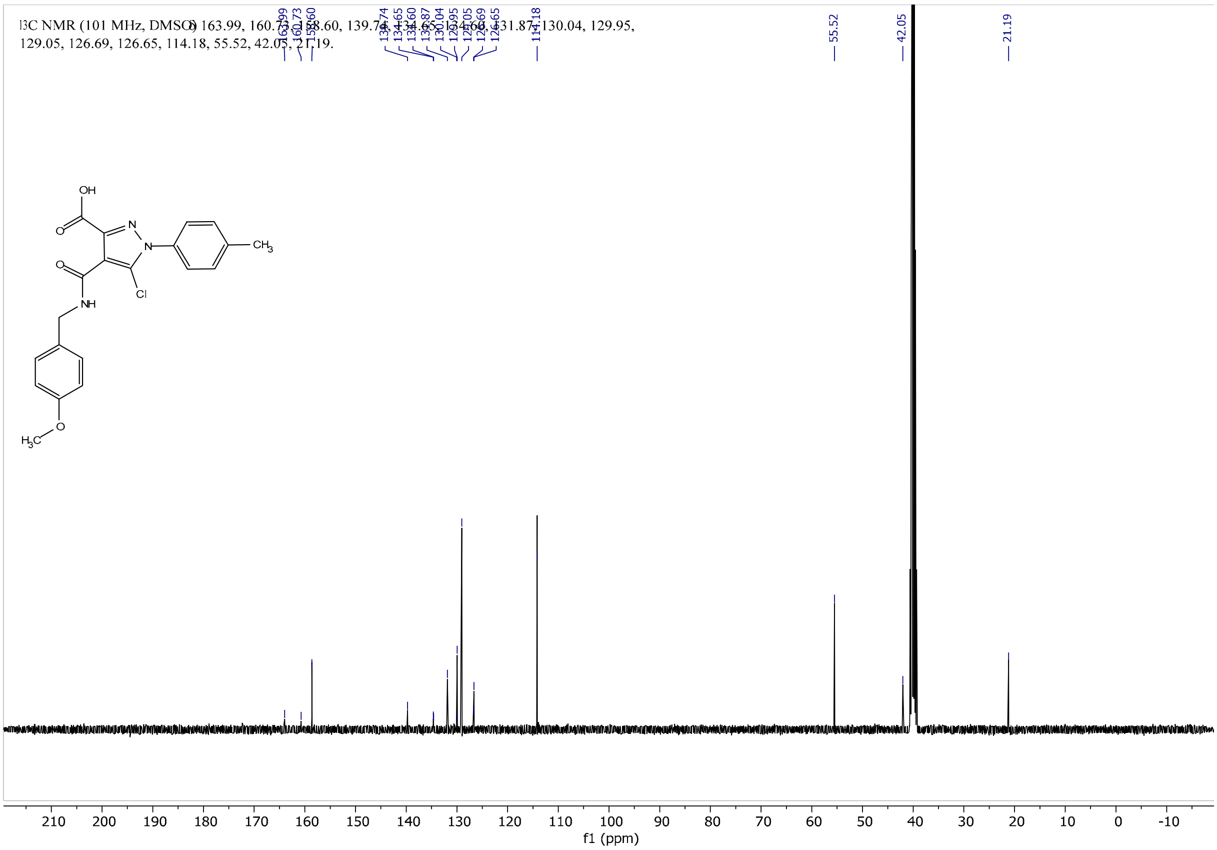

Figure S13: 5-Chloro-4-(methylcarbamoyl)-1-(4-nitrophenyl)-1*H*-pyrazole-3-carboxylic acid (13)

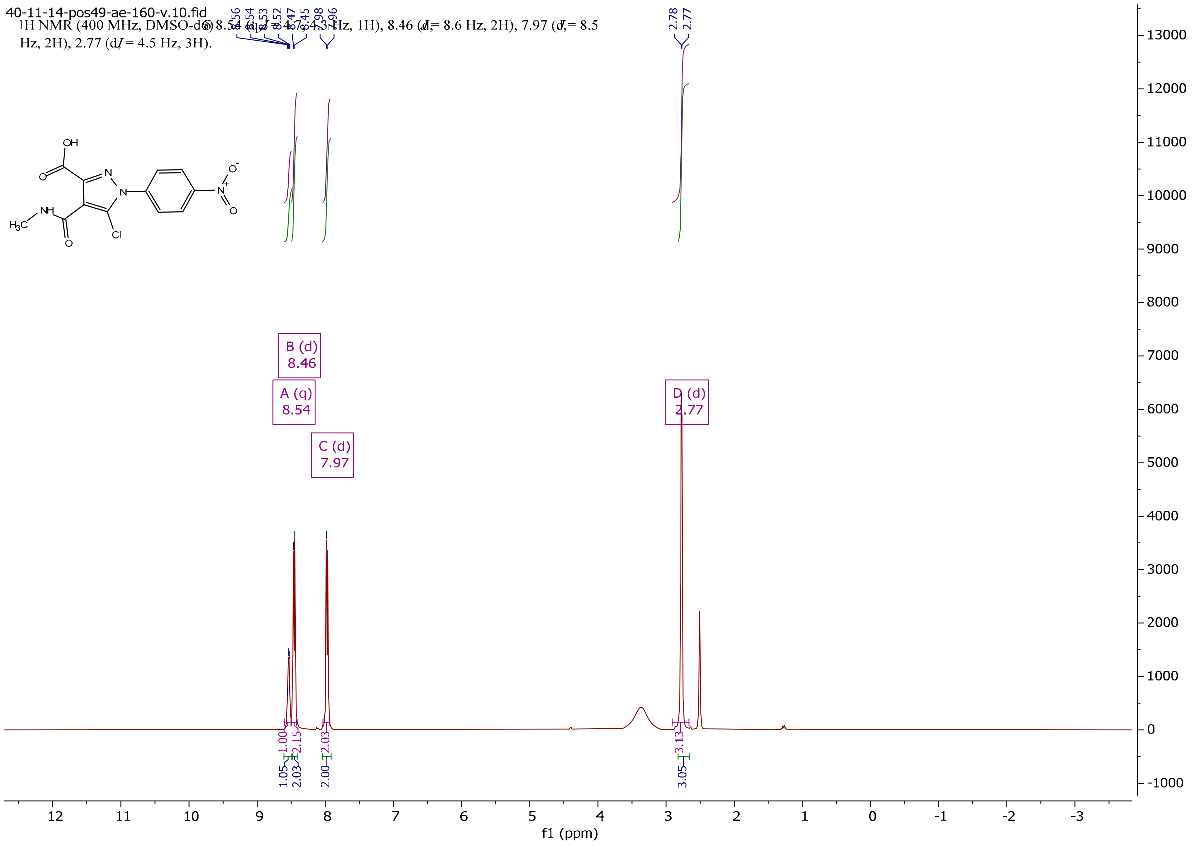

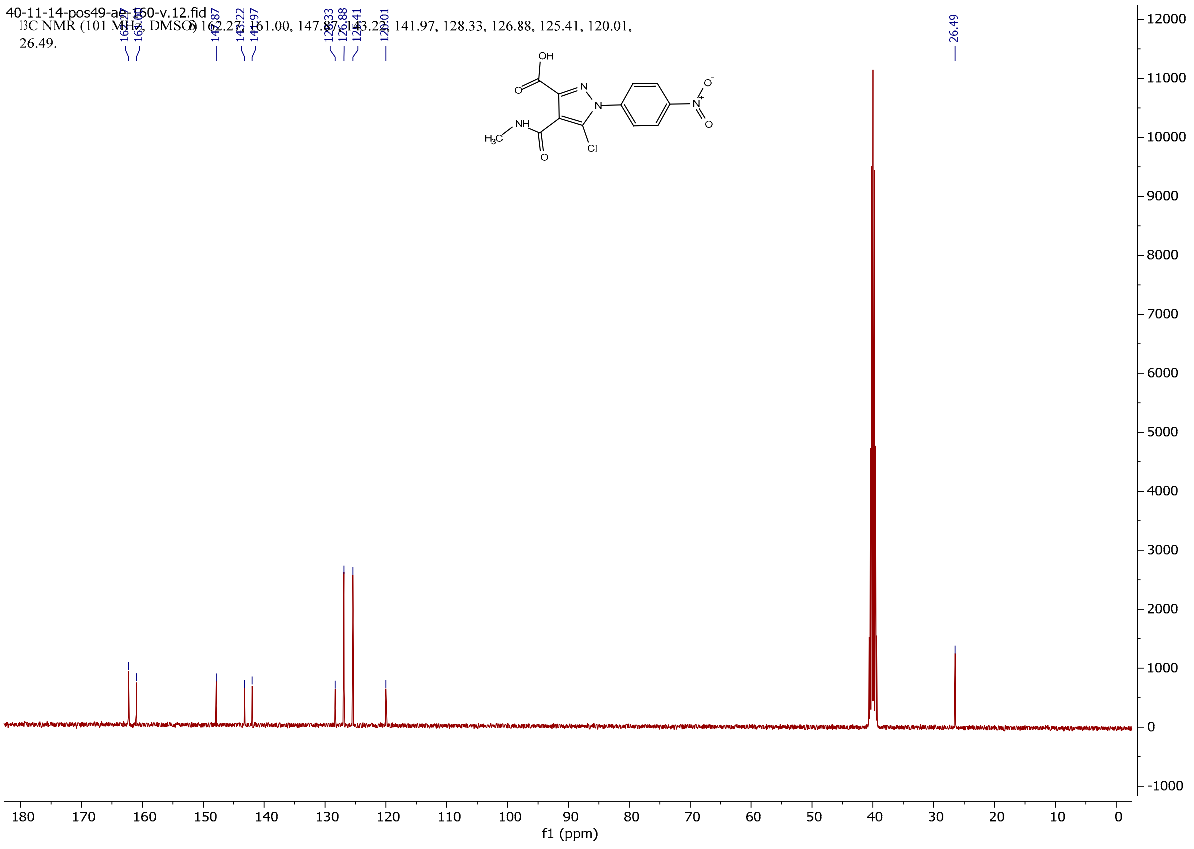

Figure S14: 5-Chloro-4-(phenylcarbamoyl)-1-(p-tolyl)-1*H*-pyrazole-3-carboxylic acid (14)

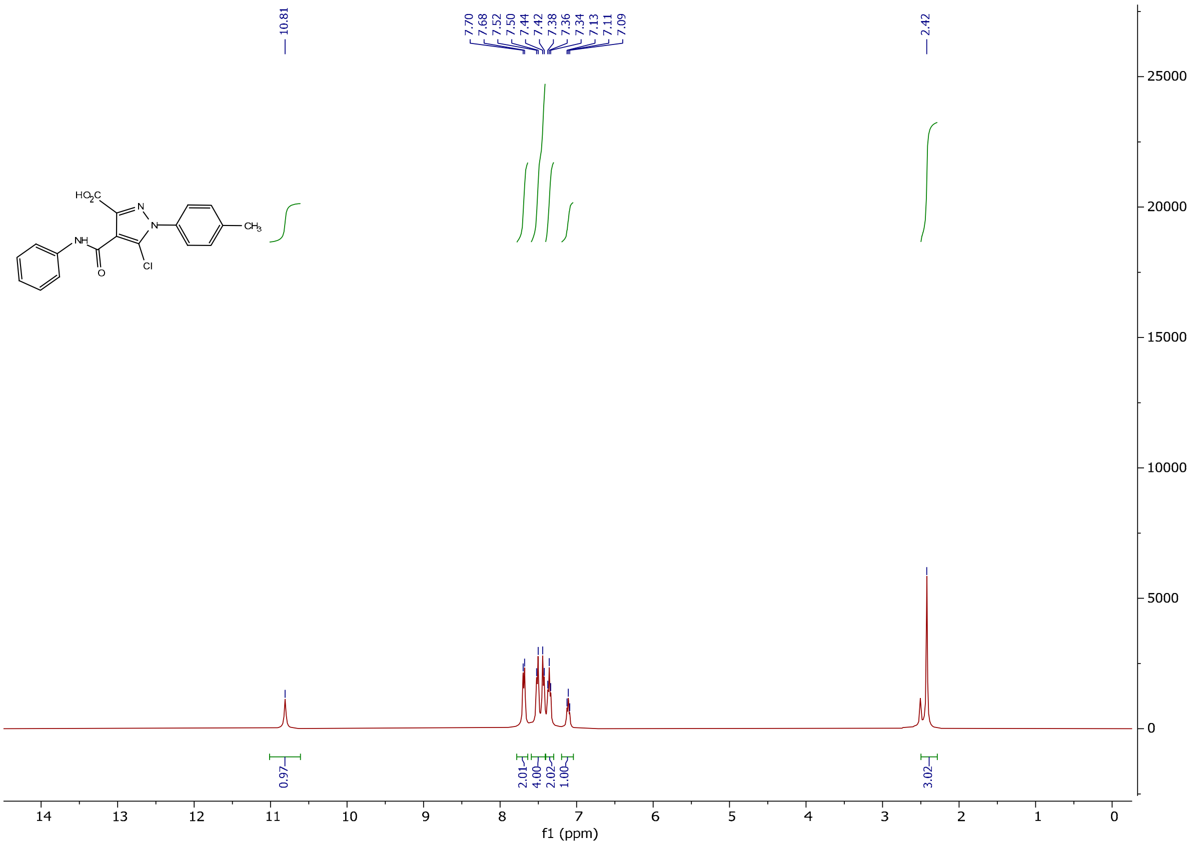

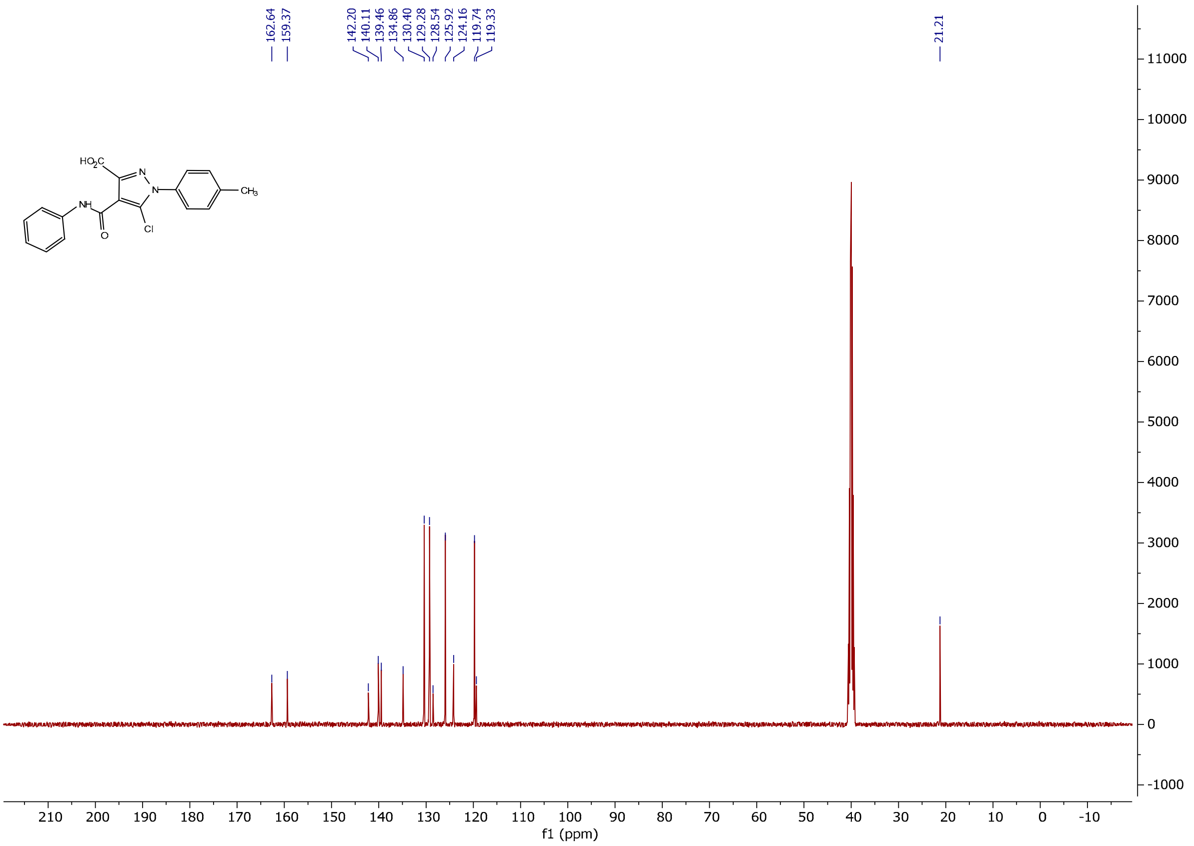

Figure S15: 3-Chloro-5-methyl-2-(p-tolyl)pyrrolo[3,4-c]pyrazole-4,6-(*2H,5H*)-dione (15)

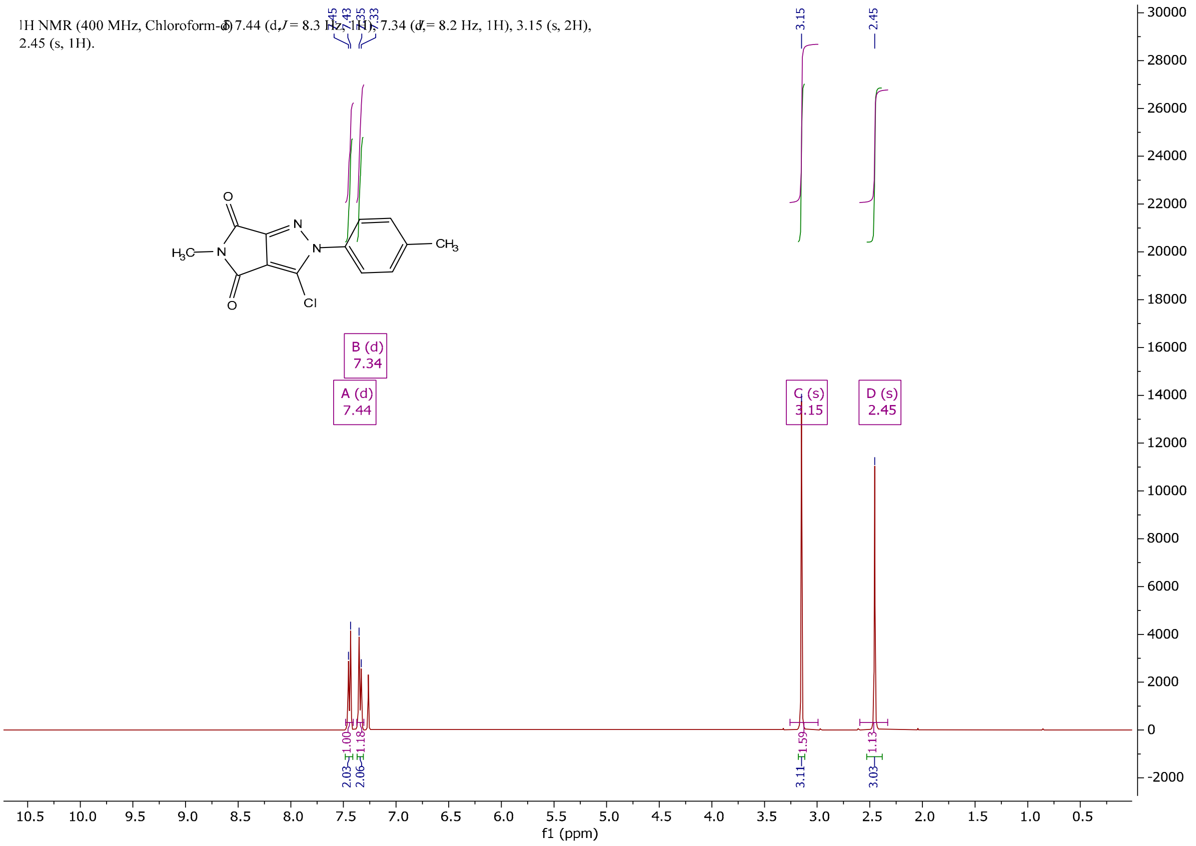

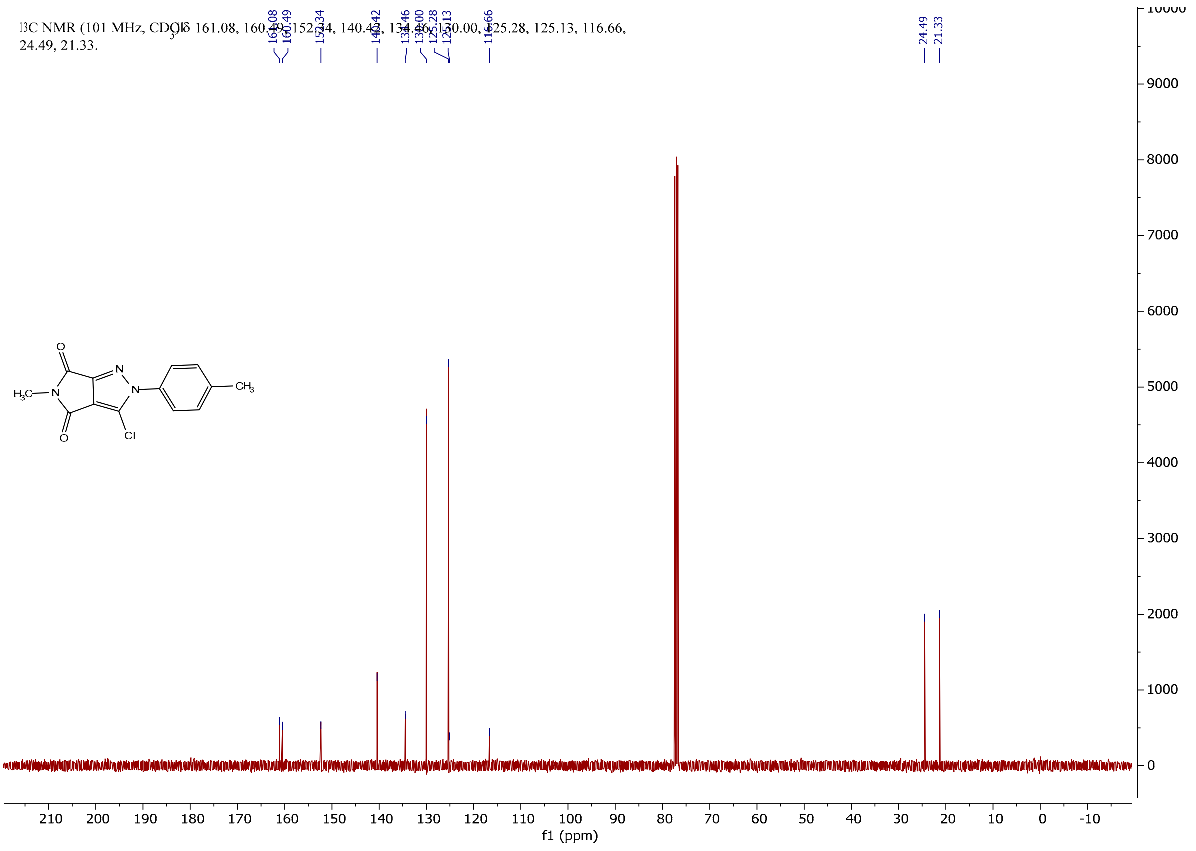

Figure S16: 3-Chloro-5-(4-methoxybenzyl)-2-(p-tolyl)pyrrolo[3,4-c]pyrazole-4,6-(*2H,5H*)-dione (16)

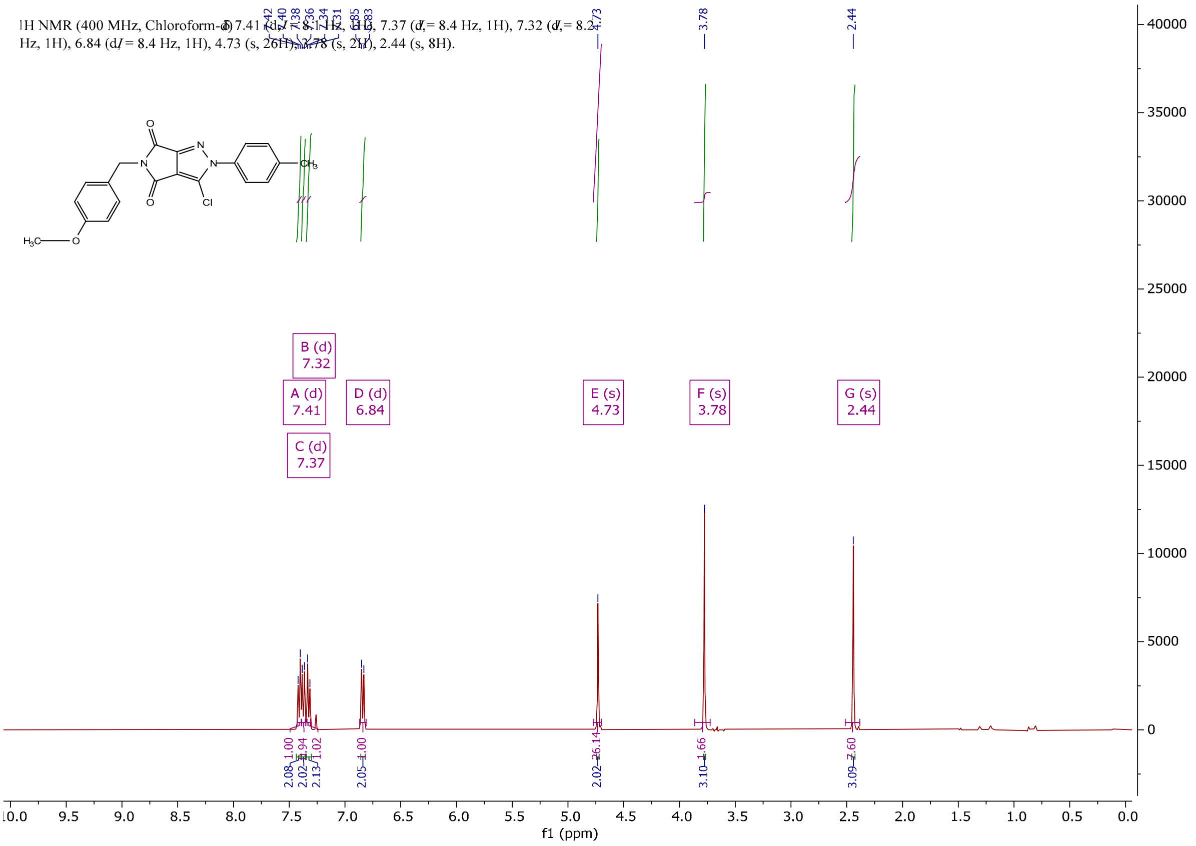

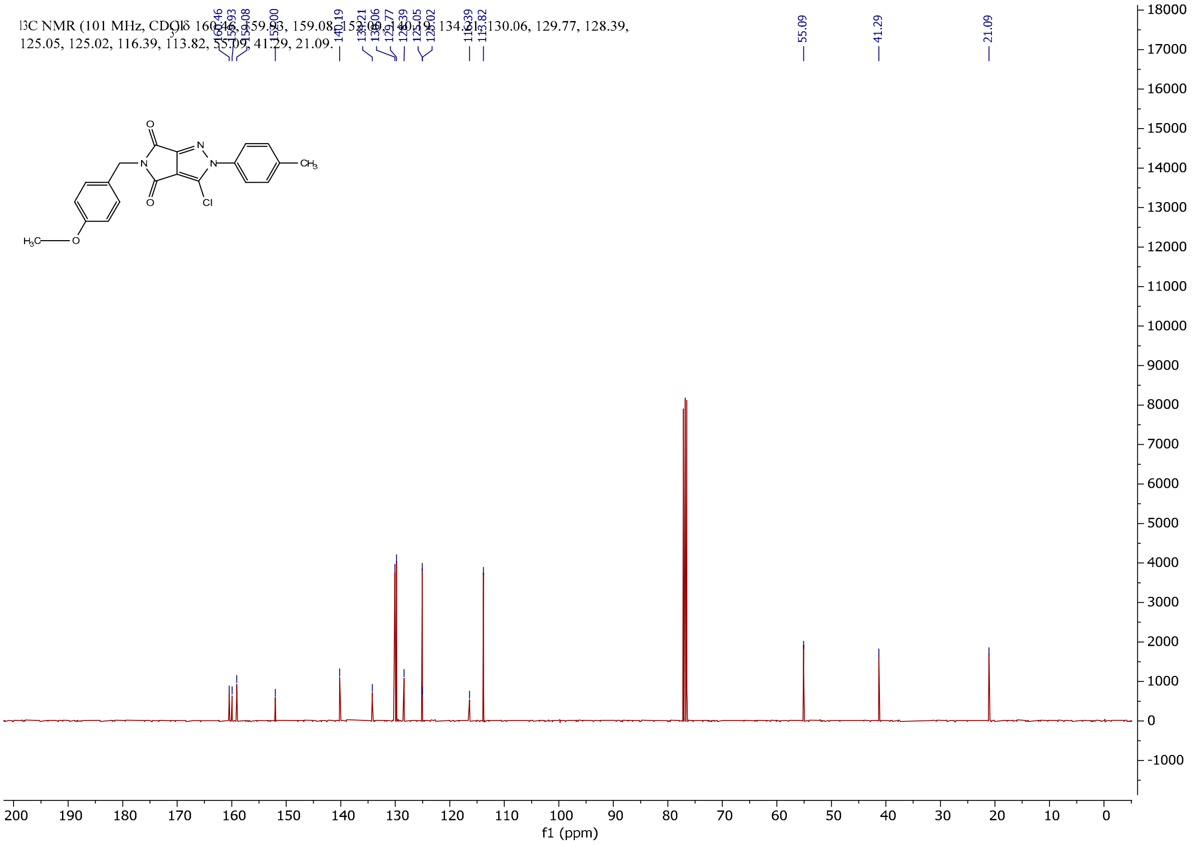

Figure S17: 3-Chloro-5-methyl-2-(4-nitrophenyl)pyrrolo[3,4-c]pyrazole-4,6-(2*H*,5*H*)-dione (17)

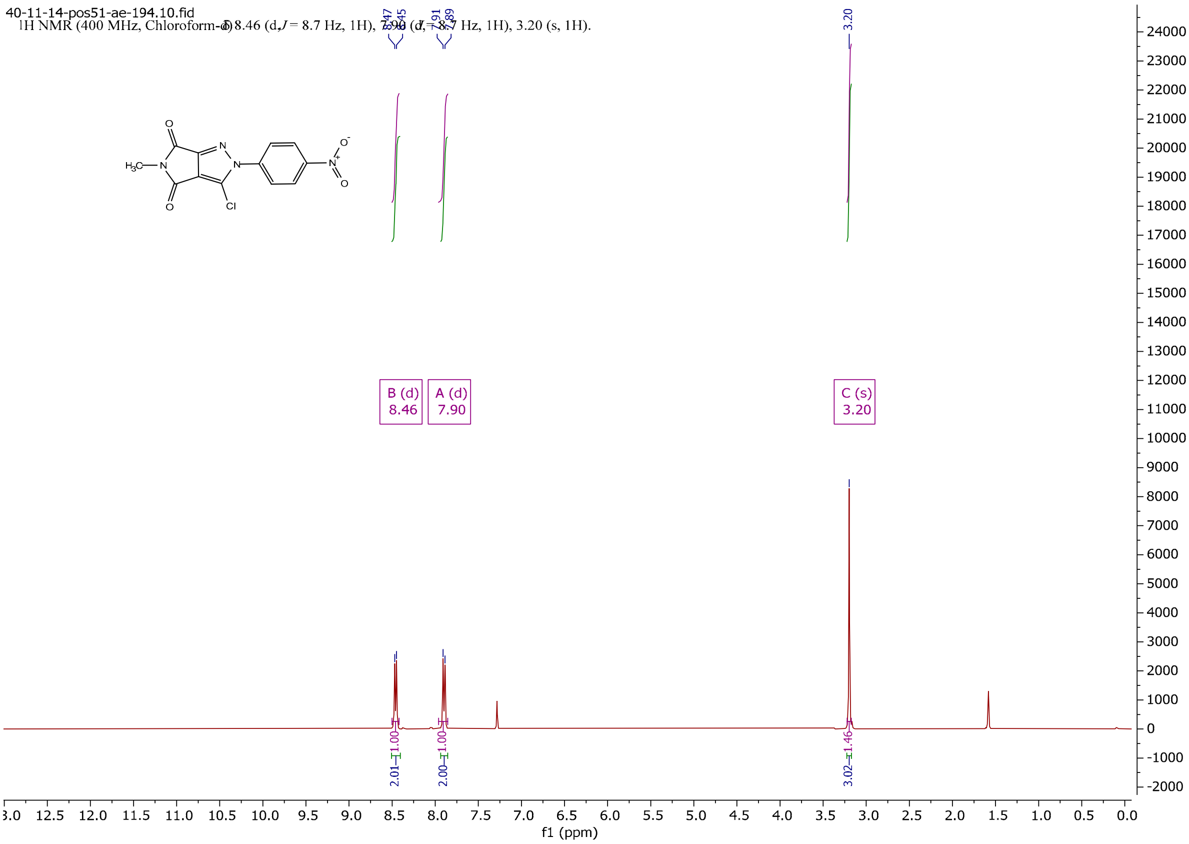

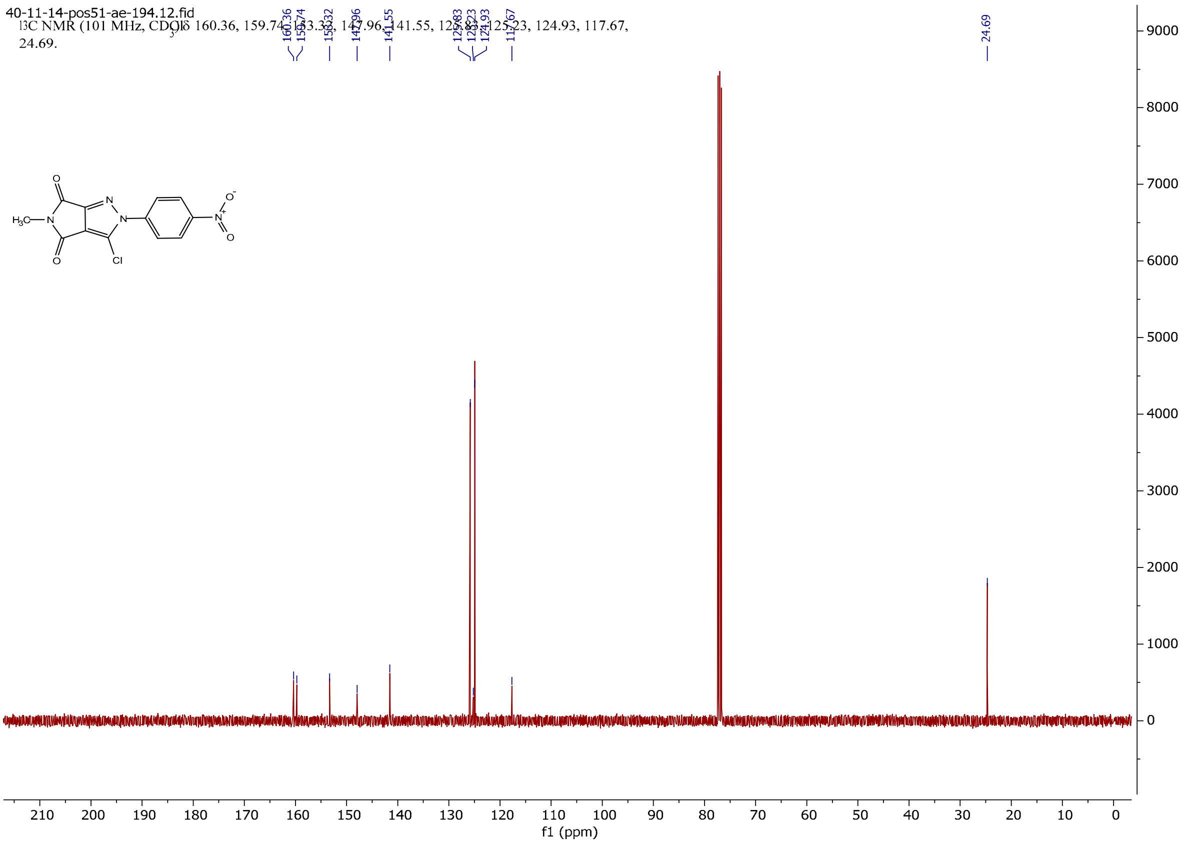

Figure S18: 5-Methyl-2,3-di-p-tolylpyrrolo[3,4-c]pyrazole-4,6-(*2H,5H*)-dione (19)

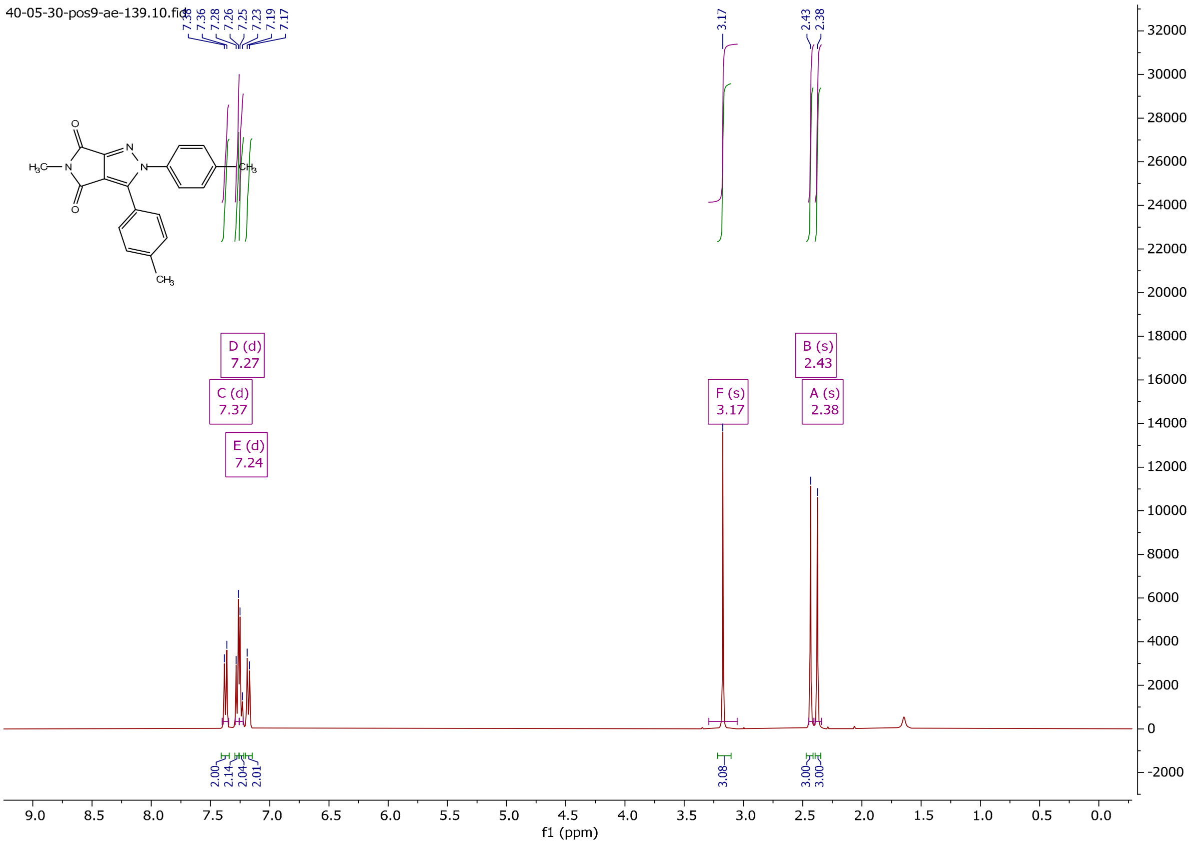

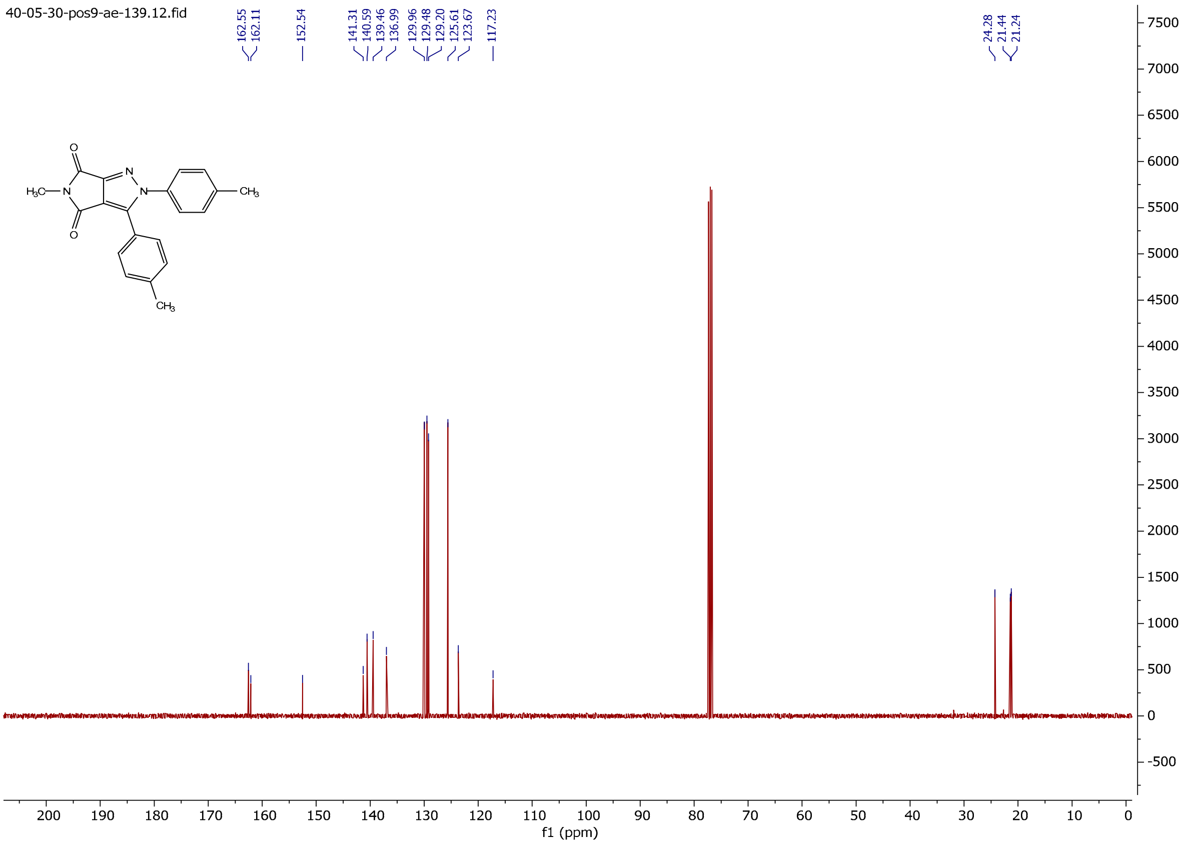

Figure S19: 3-(4-Methoxyphenyl)-5-methyl-2-(p-tolyl)pyrrolo[3,4-c]pyrazole-4,6-(*2H,5H*)-dione (20)

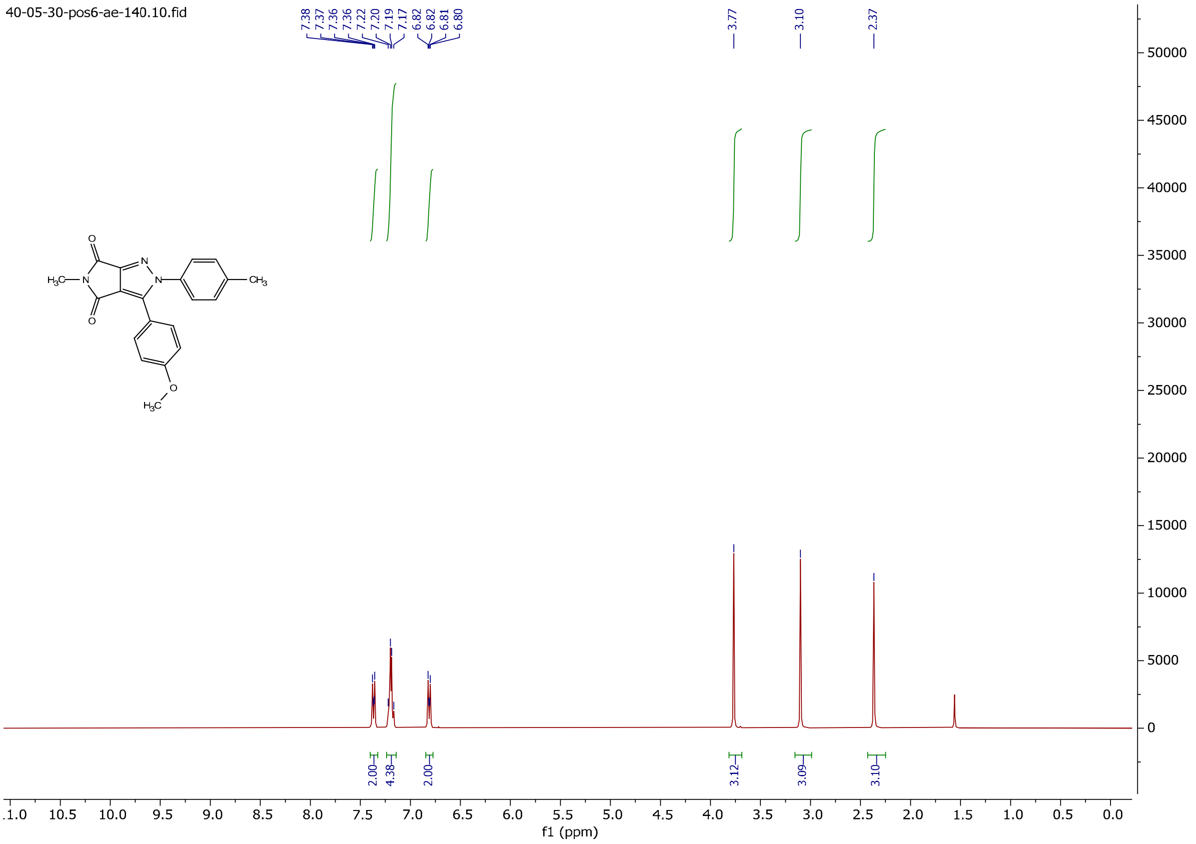

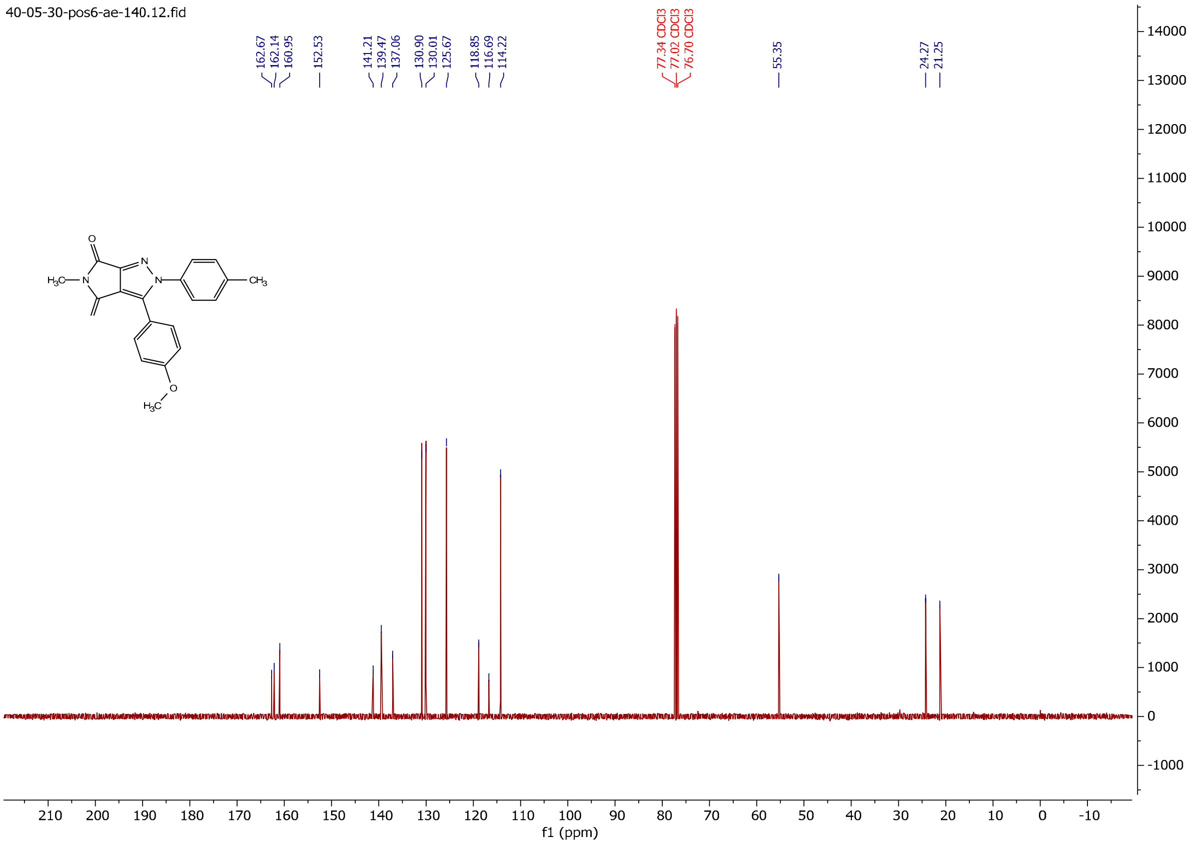

Figure S20: 3-(3-Methoxyphenyl)-5-methyl-2-(p-tolyl)pyrrolo[3,4-c]pyrazole-4,6-(*2H,5H*)-dione (21)

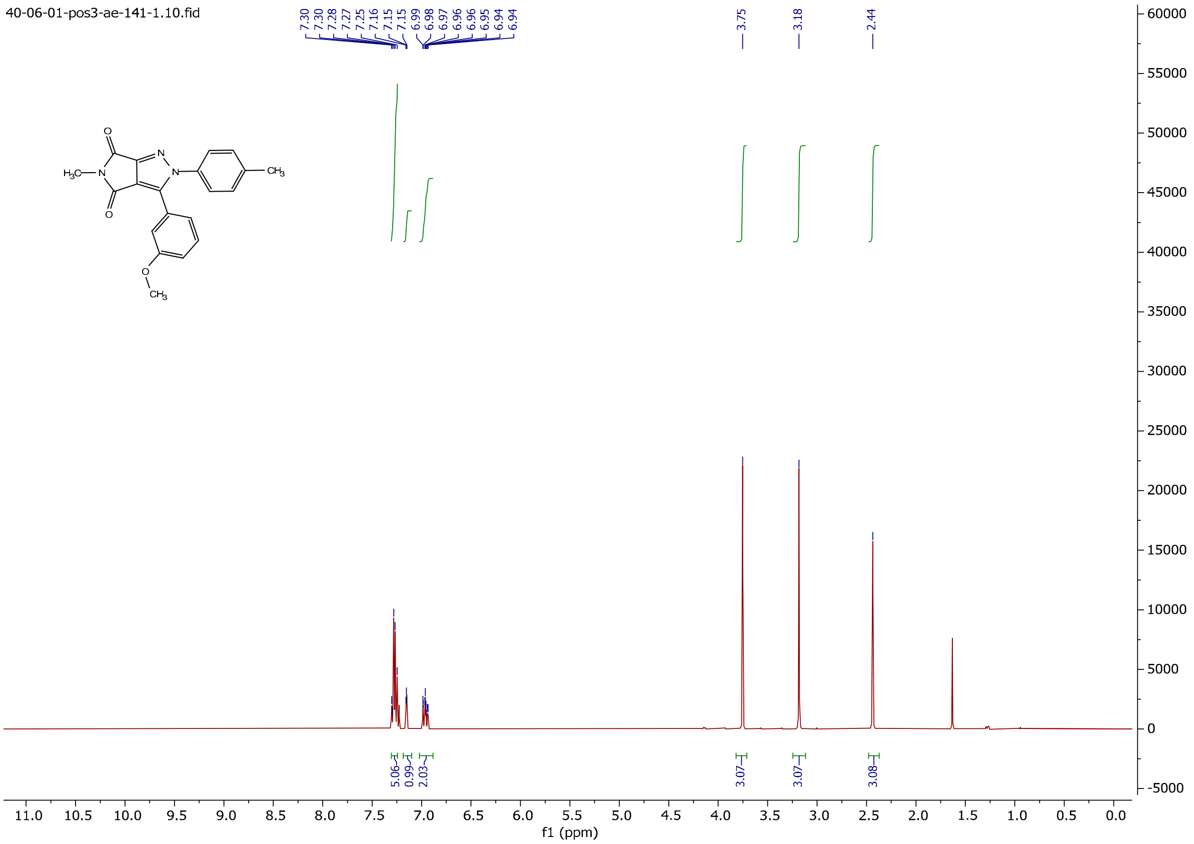

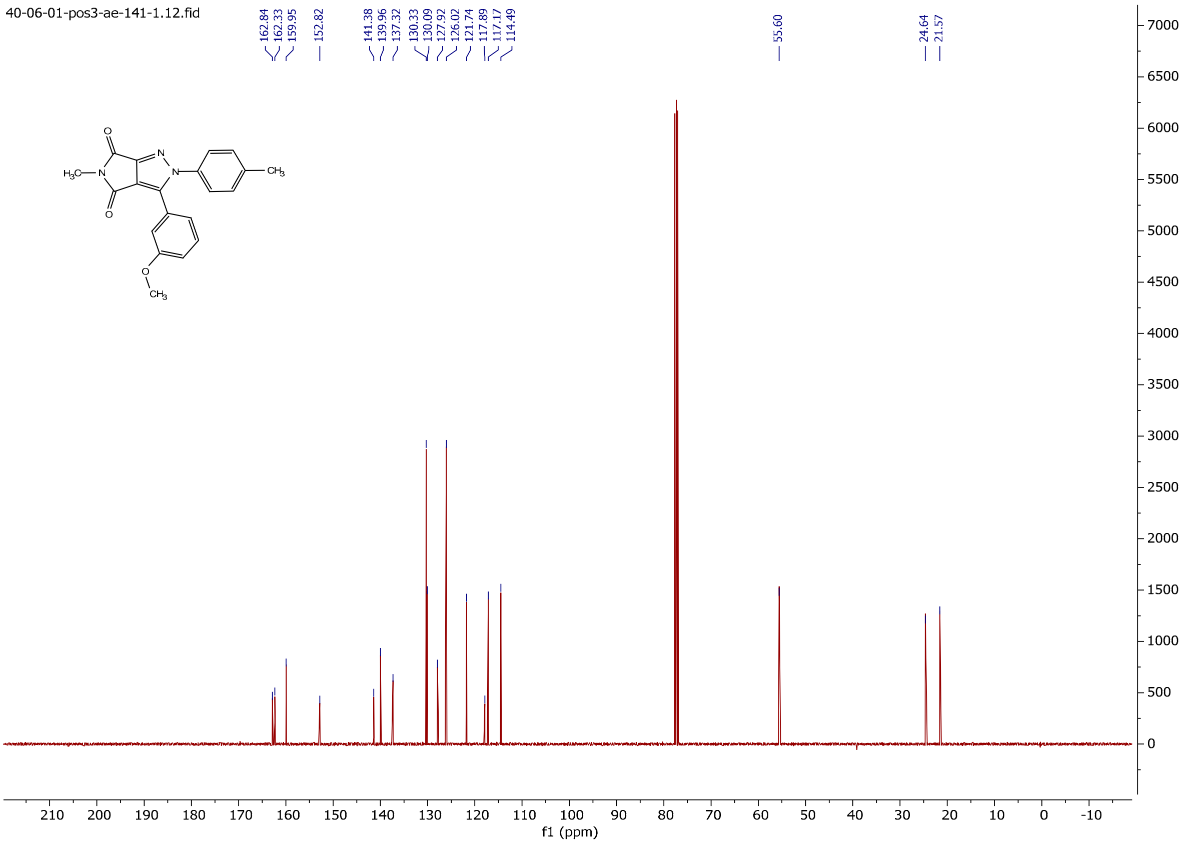

Figure S21:3-(4-Fluorophenyl)-5-methyl-2-(p-tolyl)pyrrolo[3,4-c]pyrazole-4,6-(*2H,5H*)-dione (23)

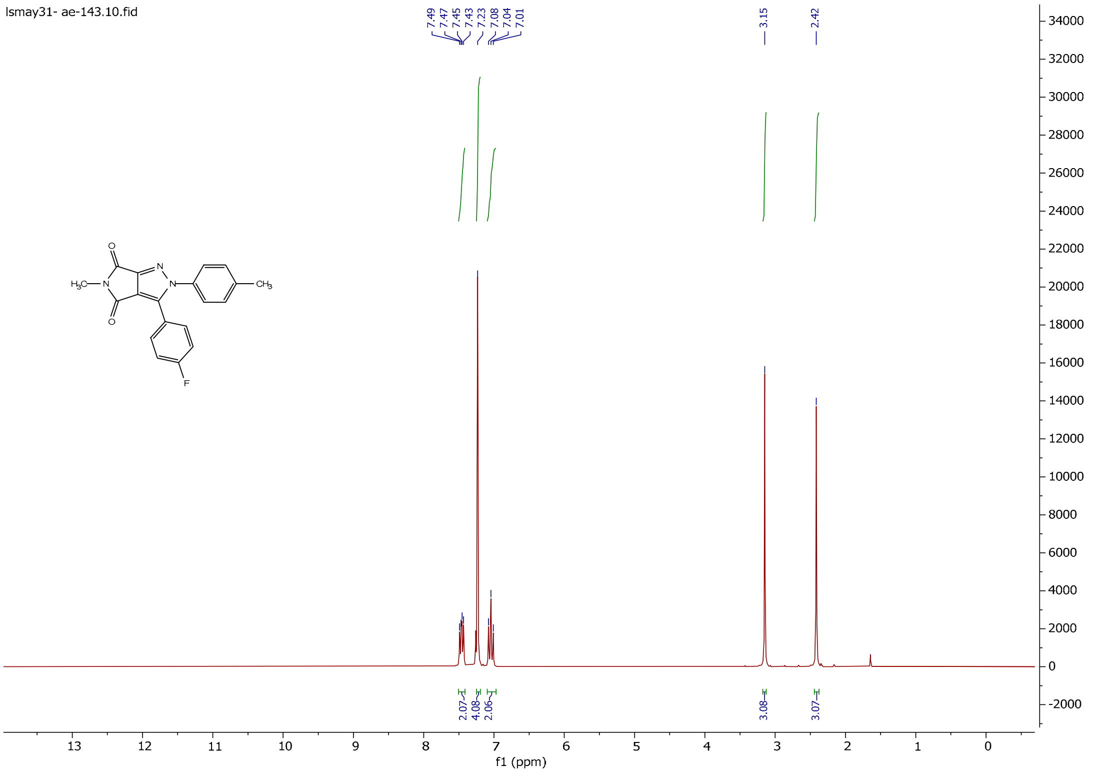

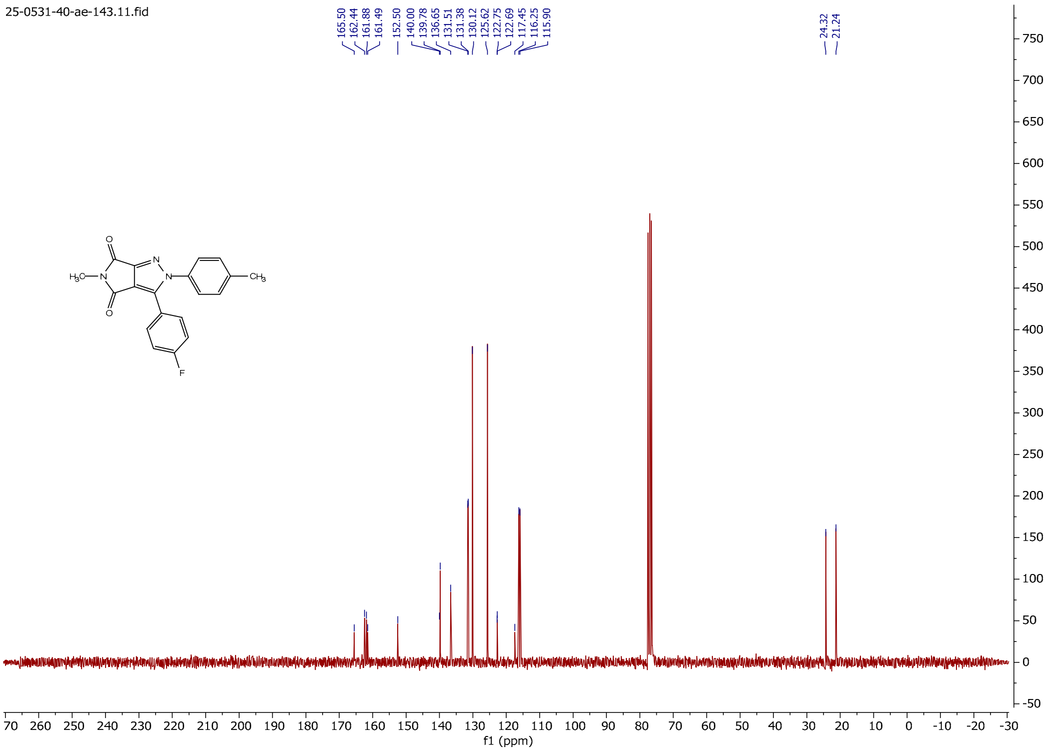

Figure S22:3-(4-Cyanophenyl)-5-methyl-2-(p-tolyl)pyrrolo[3,4-c]pyrazole-4,6-(*2H,5H*)-dione (24)

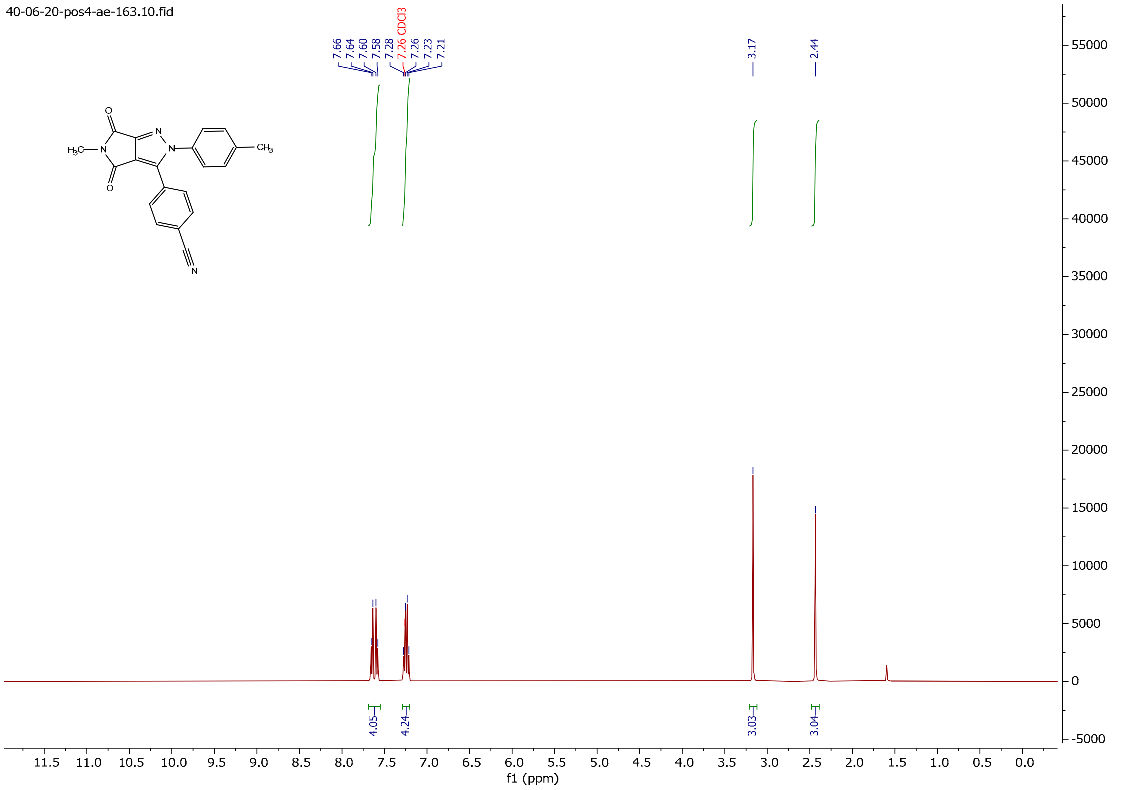

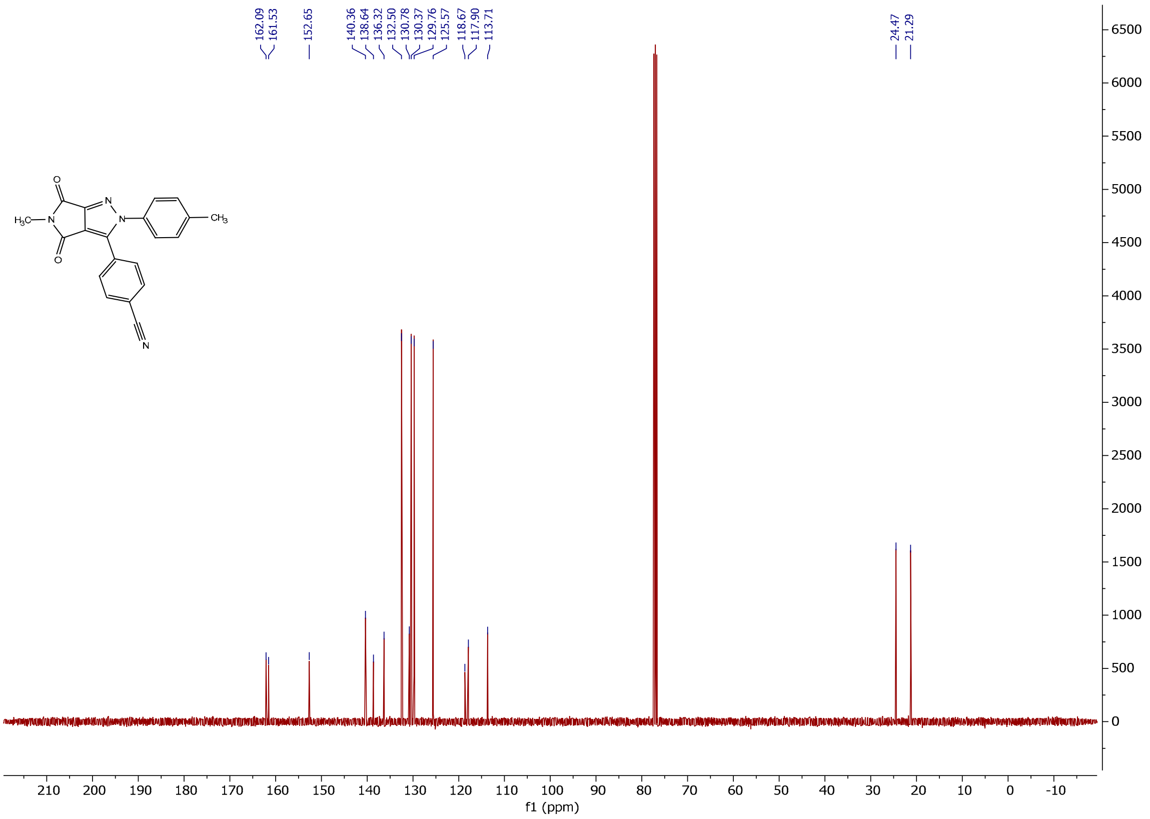

Figure S23:3-(Furan-2-yl)-5-methyl-2-(p-tolyl)pyrrolo[3,4-c]pyrazole-4,6-(*2H,5H*)-dione (25)

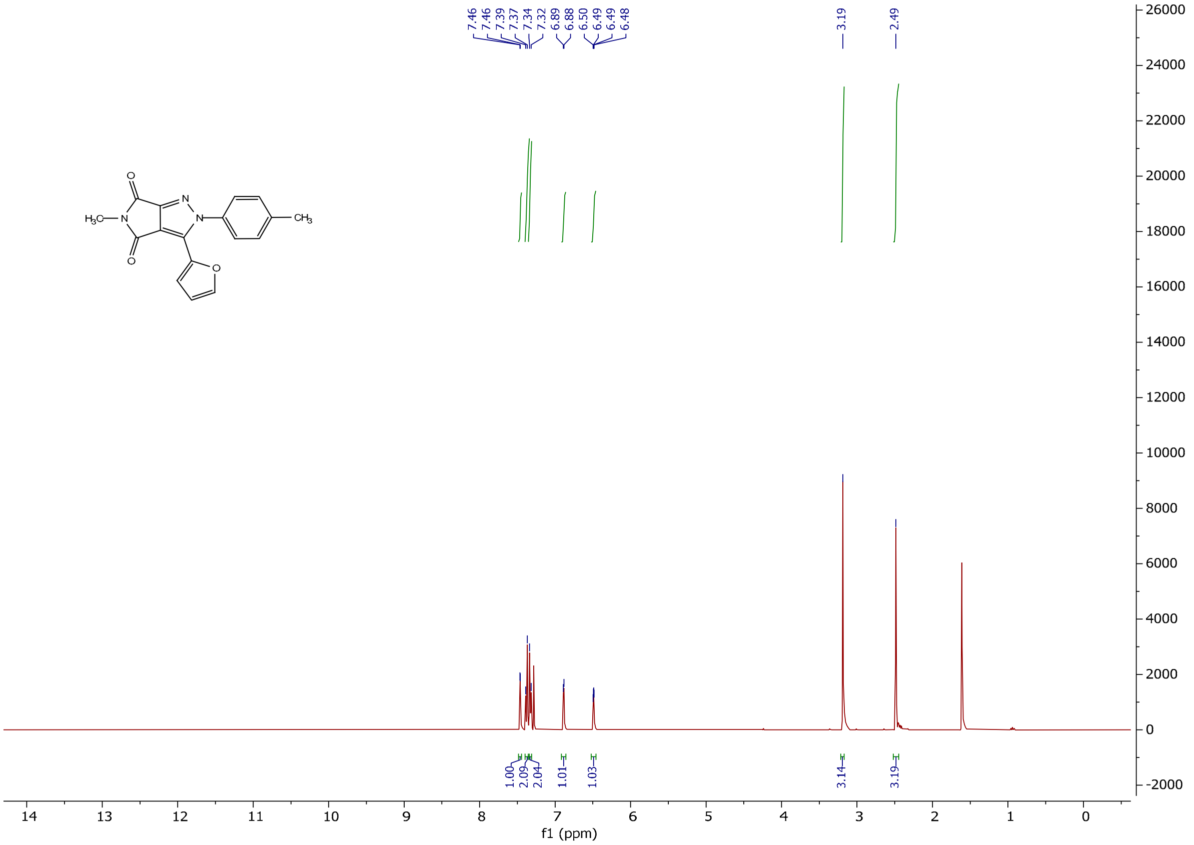
7.46
7.46
7.39
7.37
7.34
7.32
6.89
6.88
6.50
6.49
6.49
6.48
3.19
2.49
1.00
2.09
2.04
1.01
1.03
3.14
3.19
f1 (ppm)

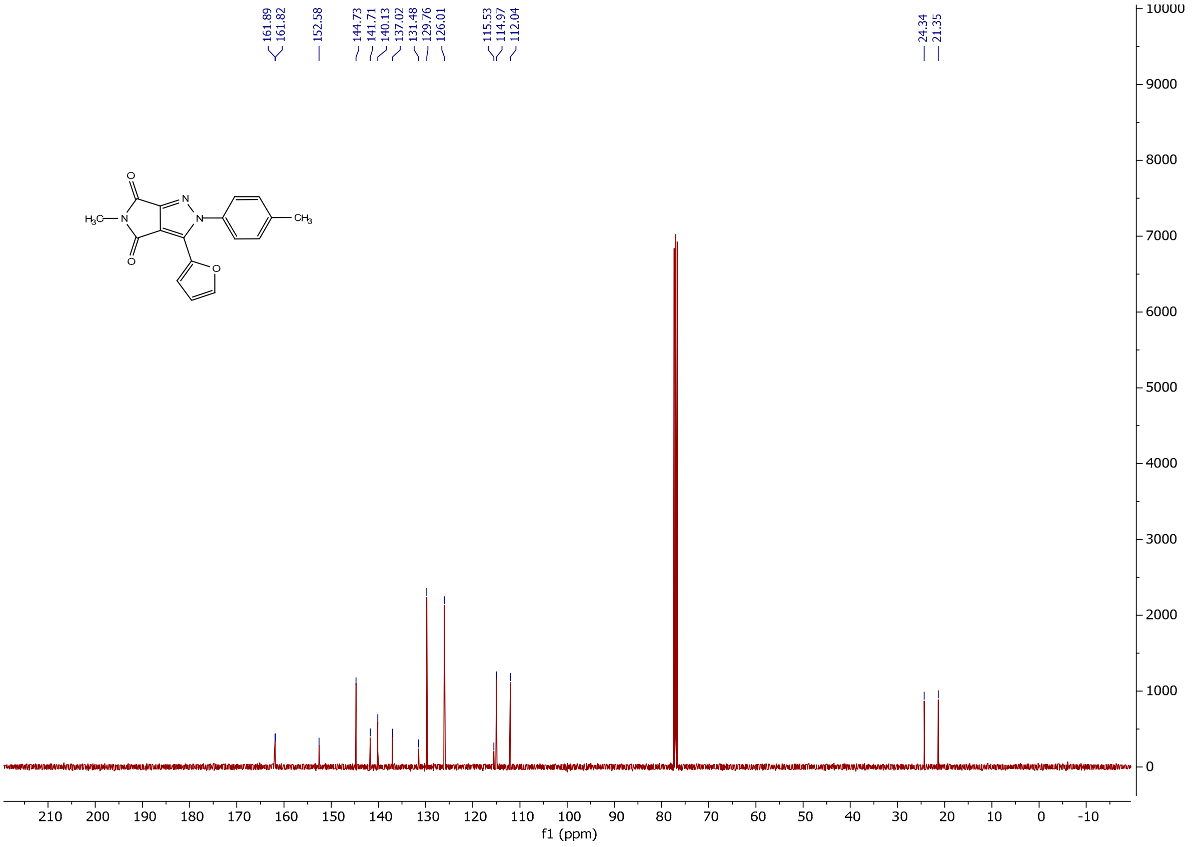
161.89
161.82
152.58
144.73
141.71
140.13
137.02
131.48
129.76
126.01
115.53
114.97
112.04
24.34
21.35
f1 (ppm)

Figure S24:3-(Furan-3-yl) -5-methyl-2-(p-tolyl)pyrrolo[3,4-c]pyrazole-4,6-(*2H,5H*)-dione (26)

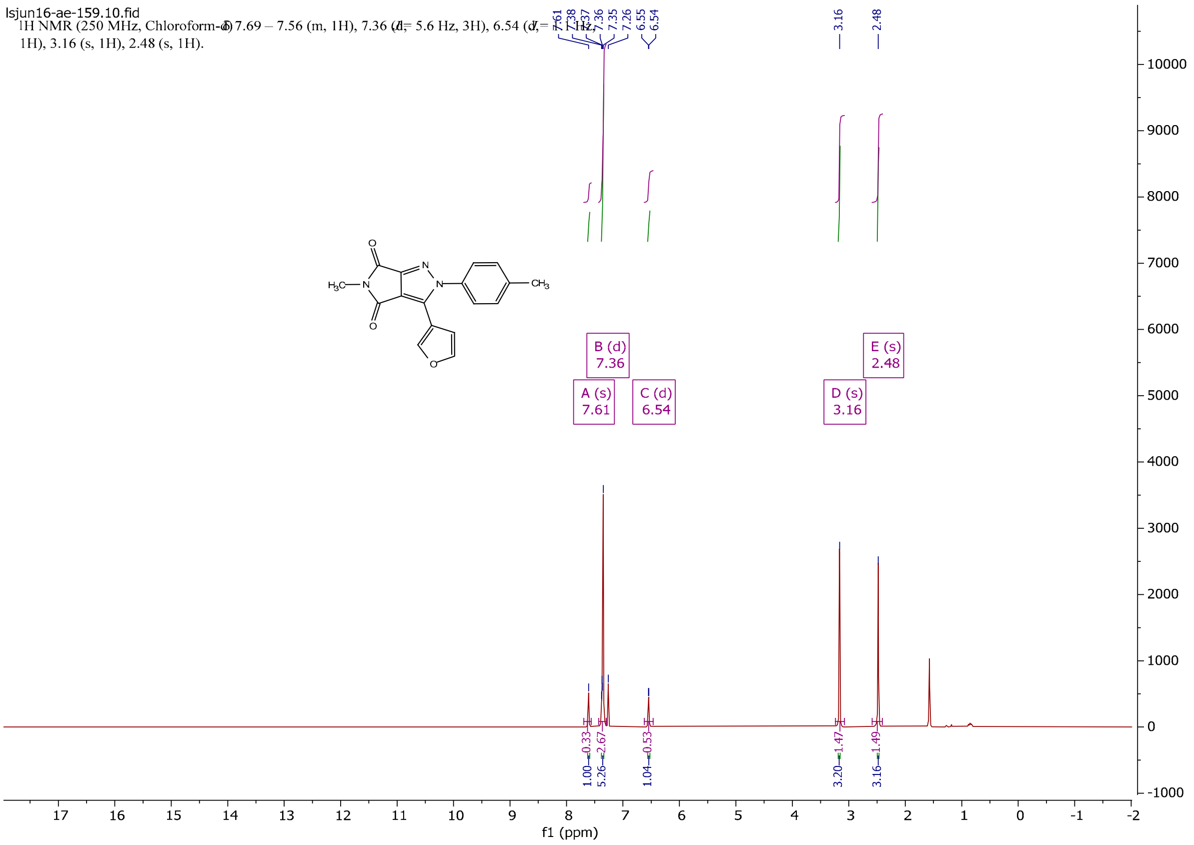

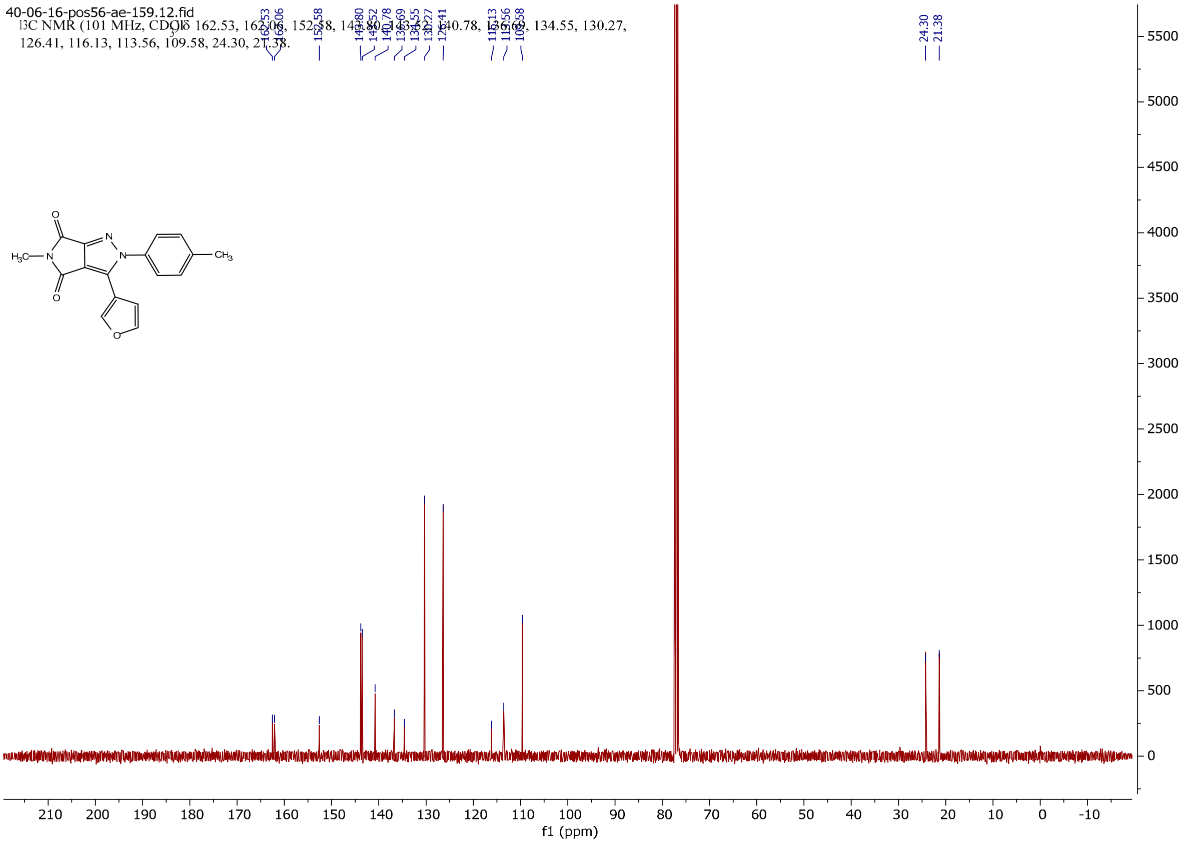

Figure S25:5-Methyl-3-(thiophen-3-yl)-2-(p-tolyl)pyrrolo[3,4-c]pyrazole-4,6-(2*H*,5*H*)-dione (27)

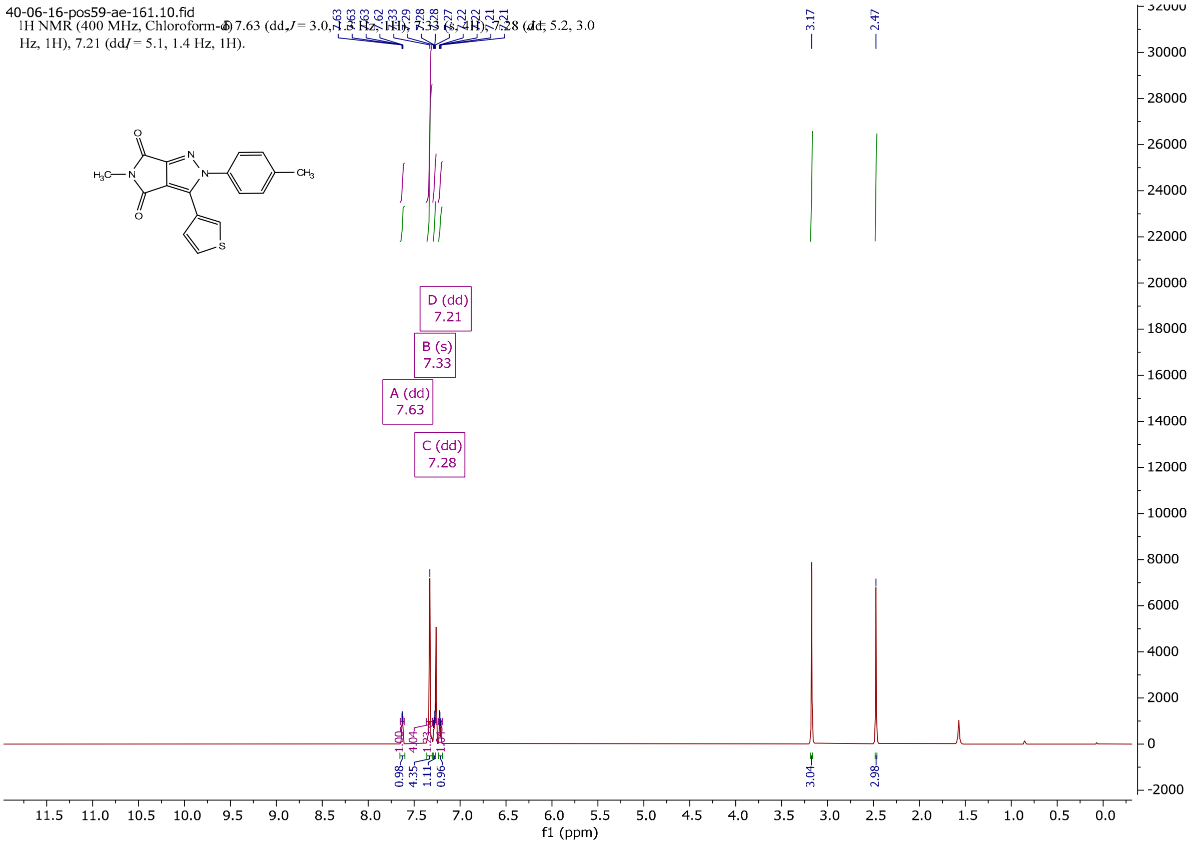

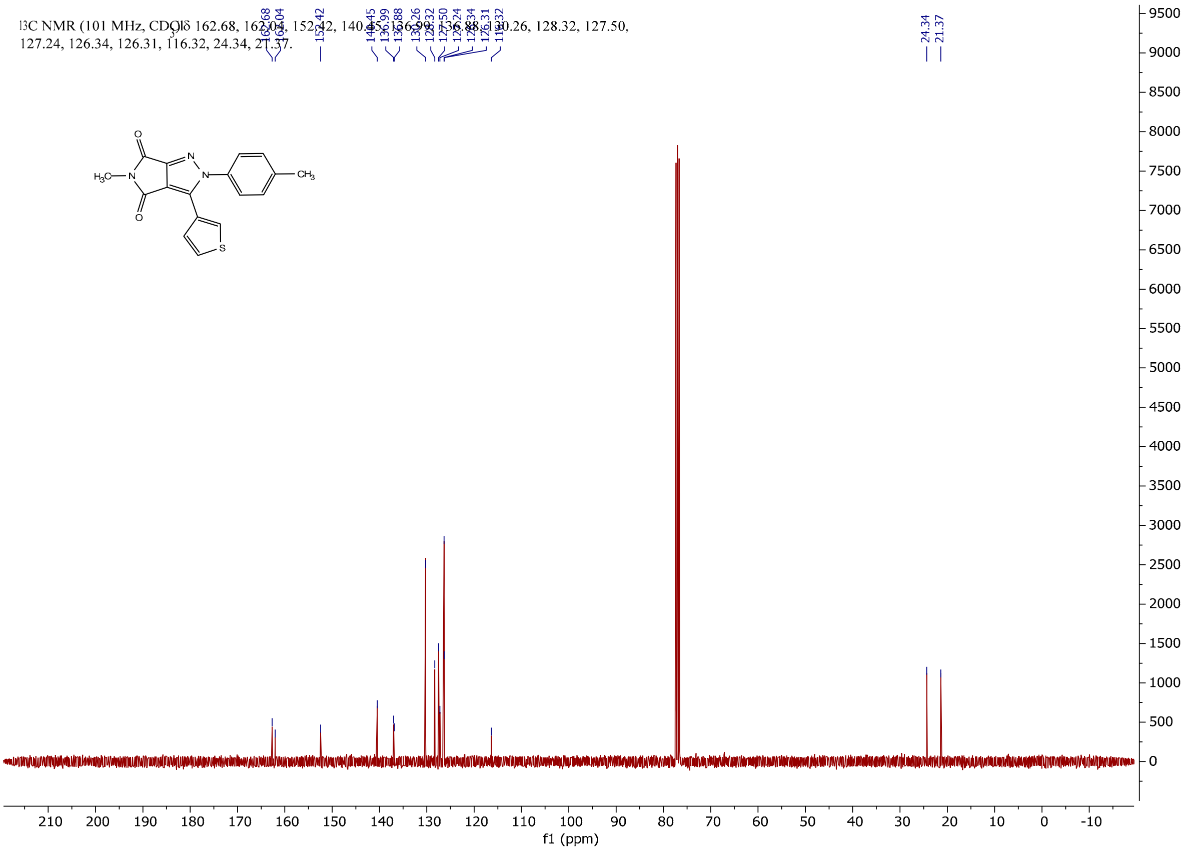

Figure S26:5-(4-Methoxybenzyl)-2,3-di-p-tolylpyrrolo[3,4-c]pyrazole-4,6-(*2H,5H*)-dione (29)

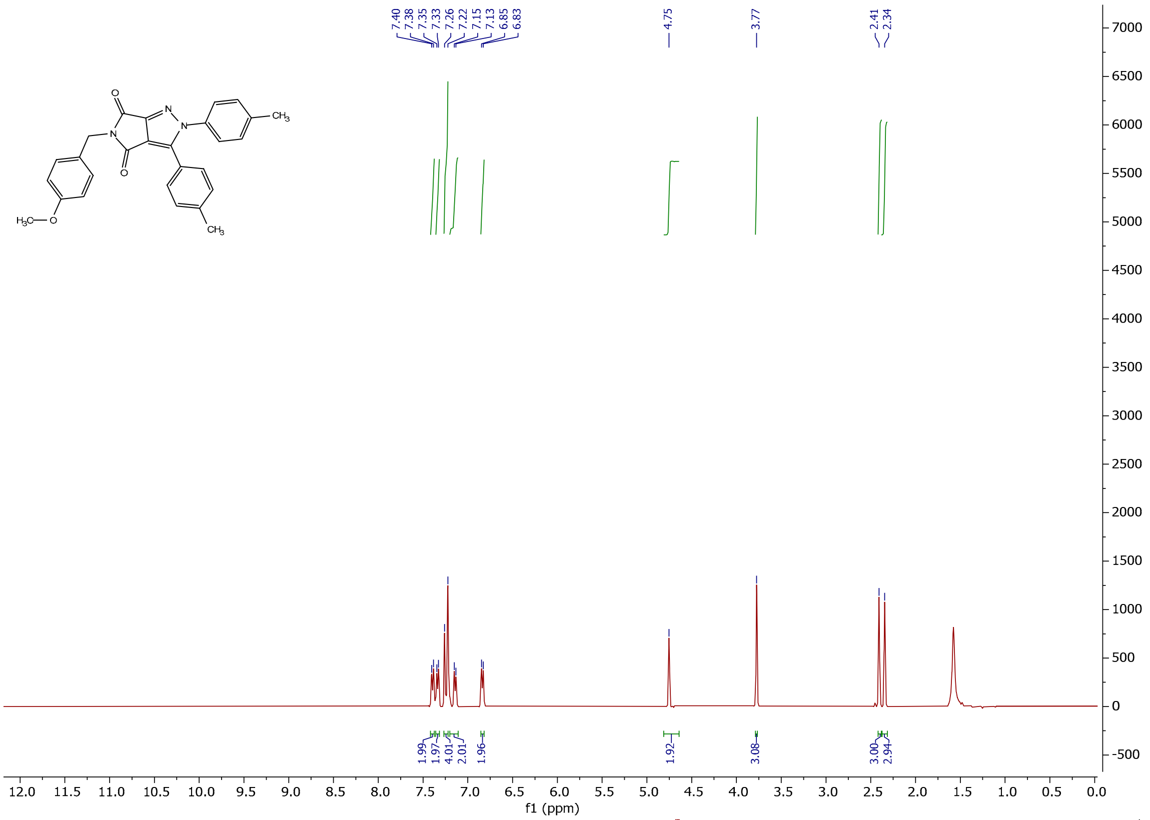

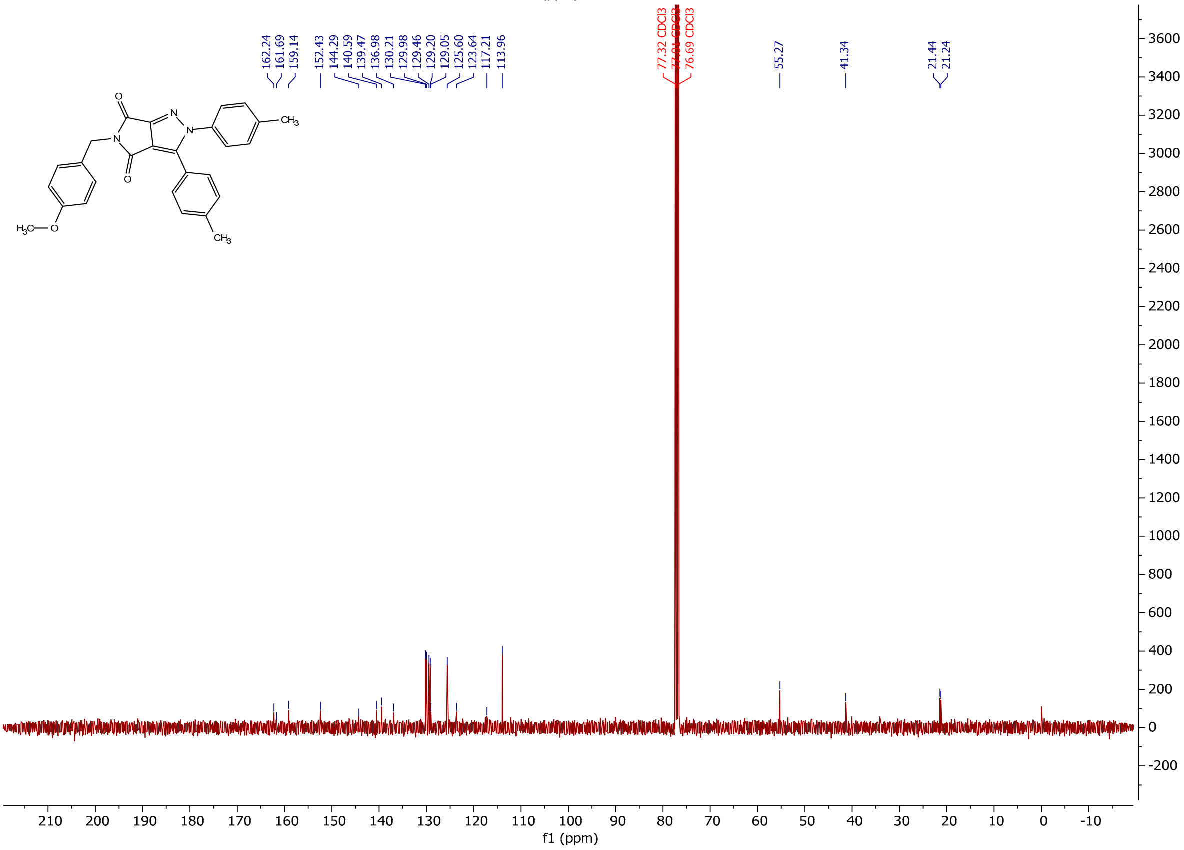

Figure S27: 5-Methyl-2-(4-nitrophenyl)-3-(p-tolyl)pyrrolo[3,4-c]pyrazole-4,6-(2*H*,5*H*)-dione (30)

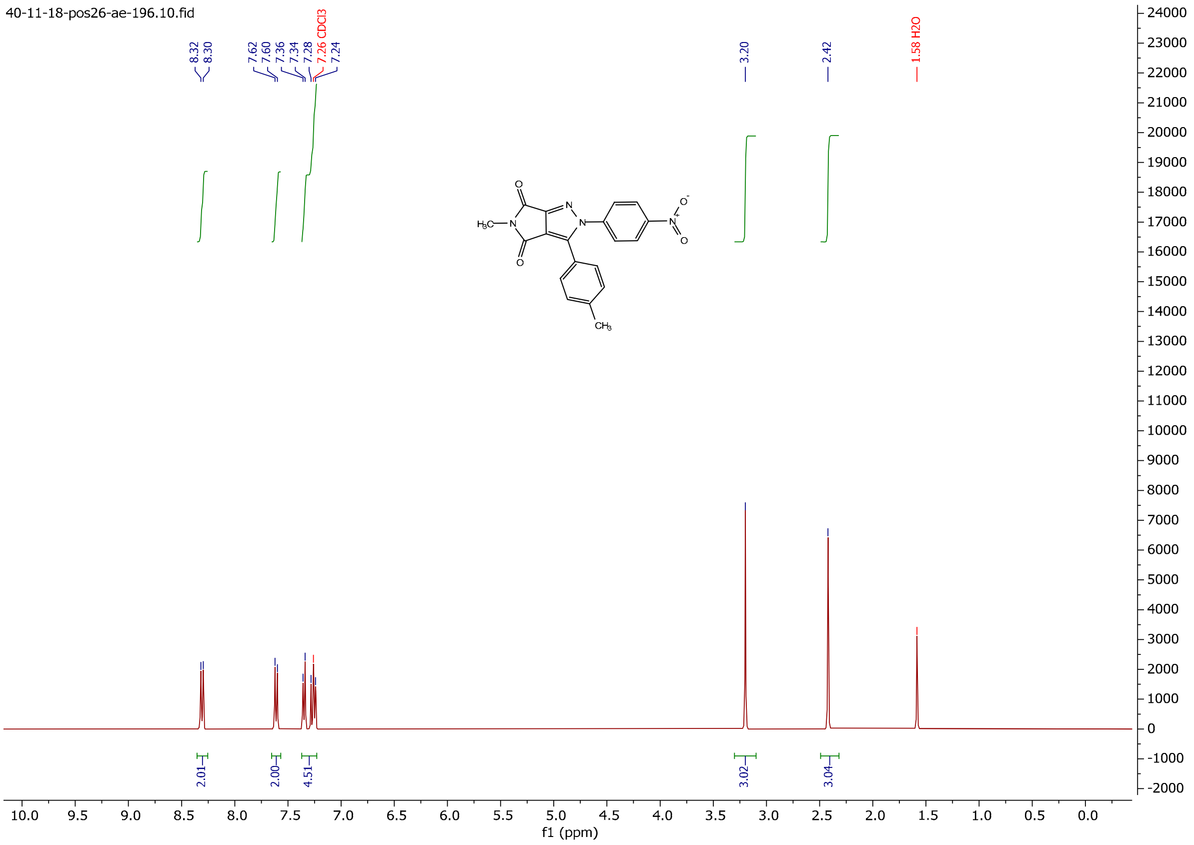

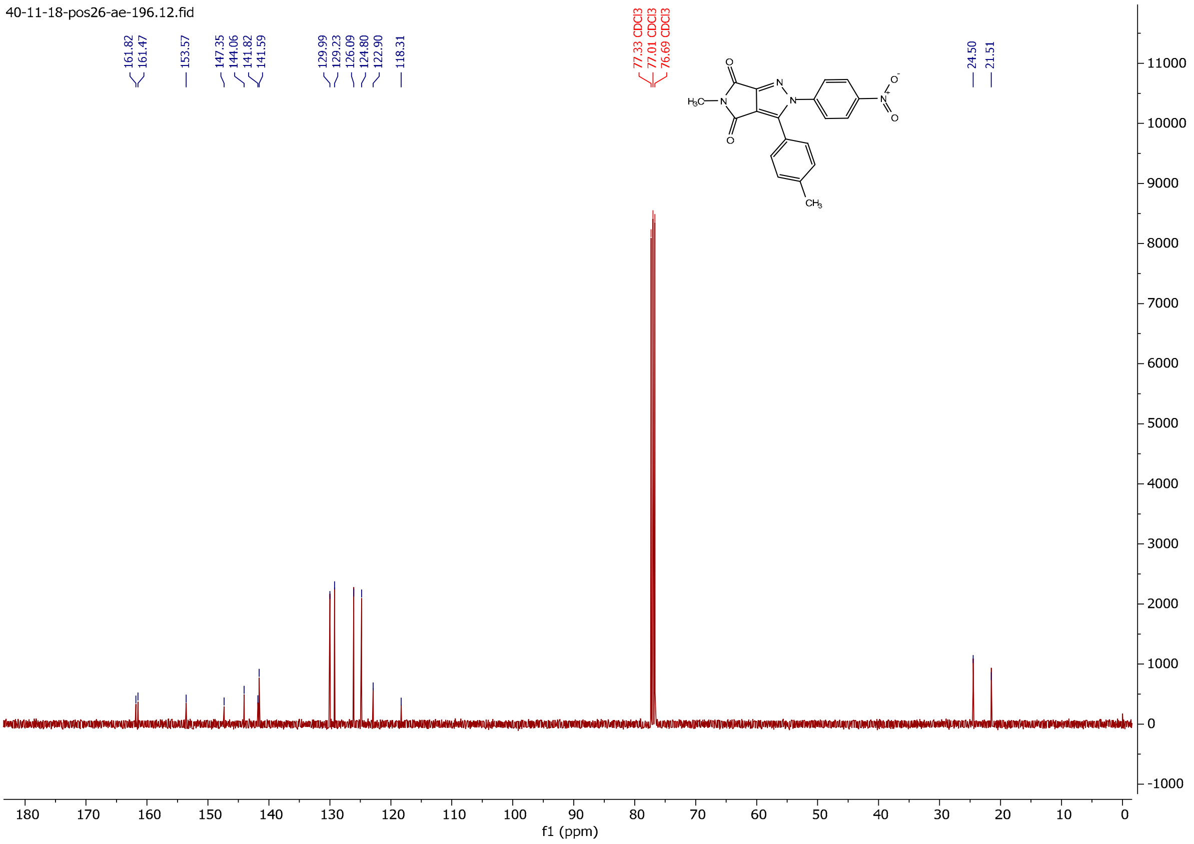

Figure S28:5-Methyl-3-(phenylamino)-2-(p-tolyl)pyrrolo[3,4-c]pyrazole-4,6-(*2H,5H*)-dione (31)

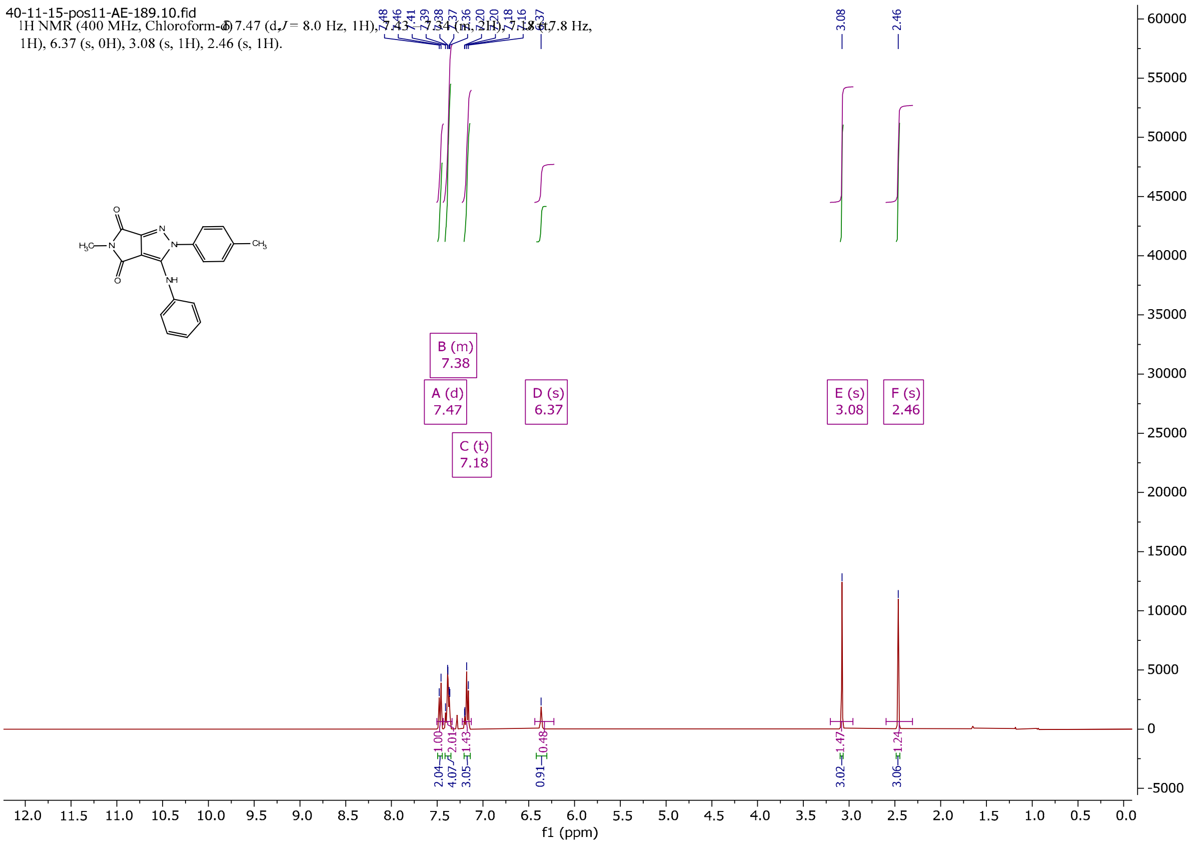

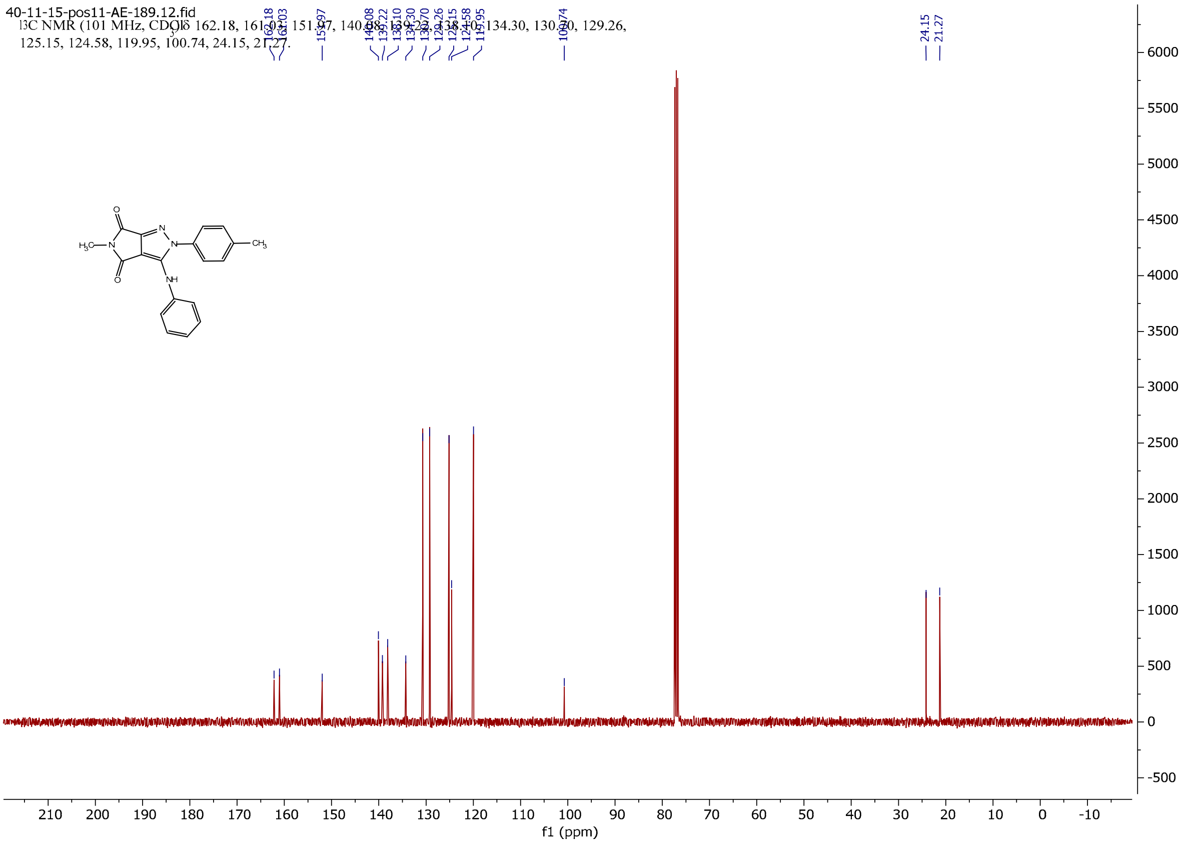

Figure S29: 3-((4-Methoxyphenyl)amino)-5-methyl-2-(p-tolyl)pyrrolo[3,4-c]pyrazole-4,6-(*2H,5H*)-dione (32)

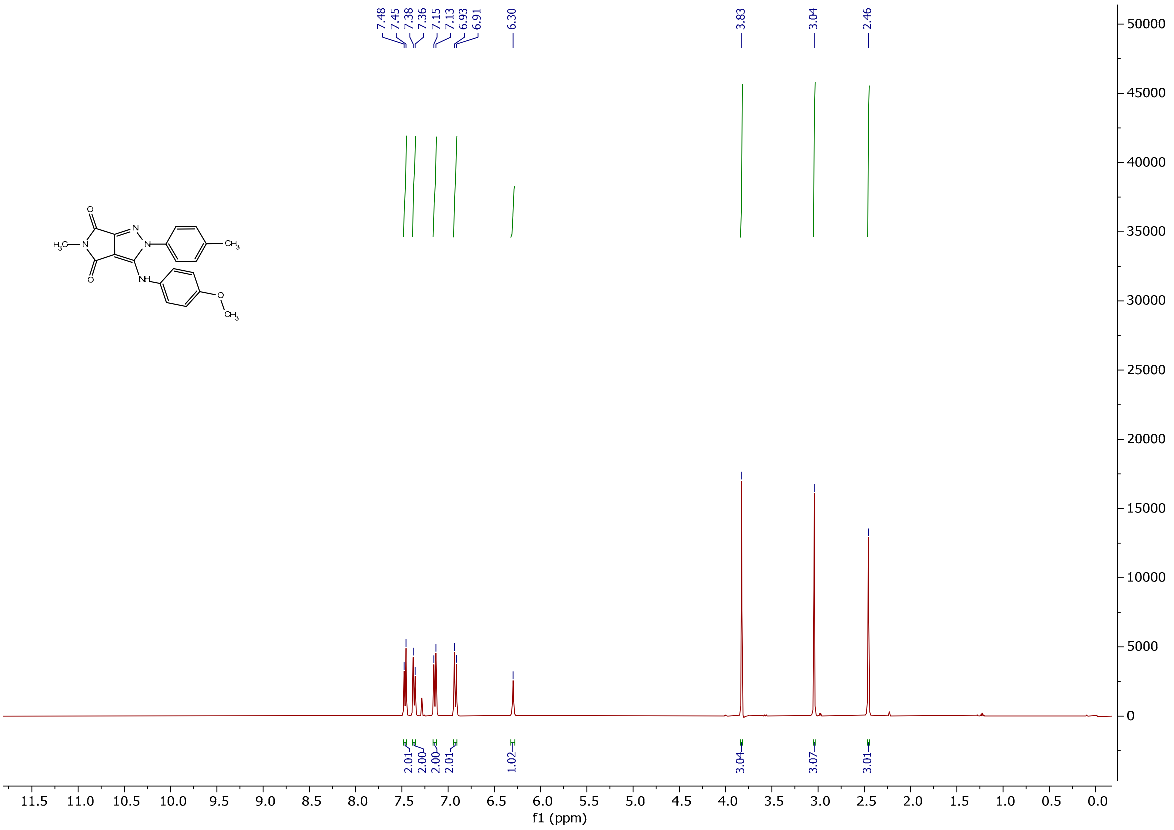

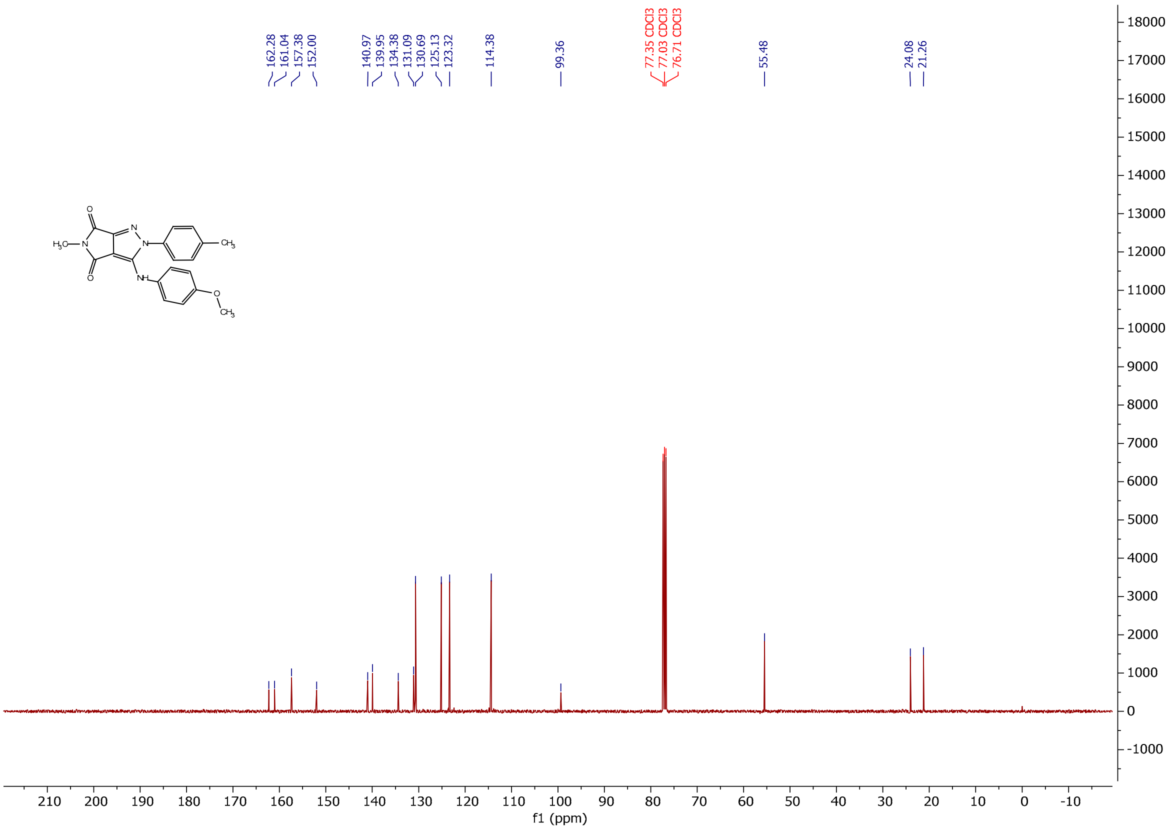

Figure S30: 3-((3-Methoxyphenyl)amino)-5-methyl-2-(p-tolyl)pyrrolo[3,4-c]pyrazole-4,6-(*2H,5H*)-dione (33)

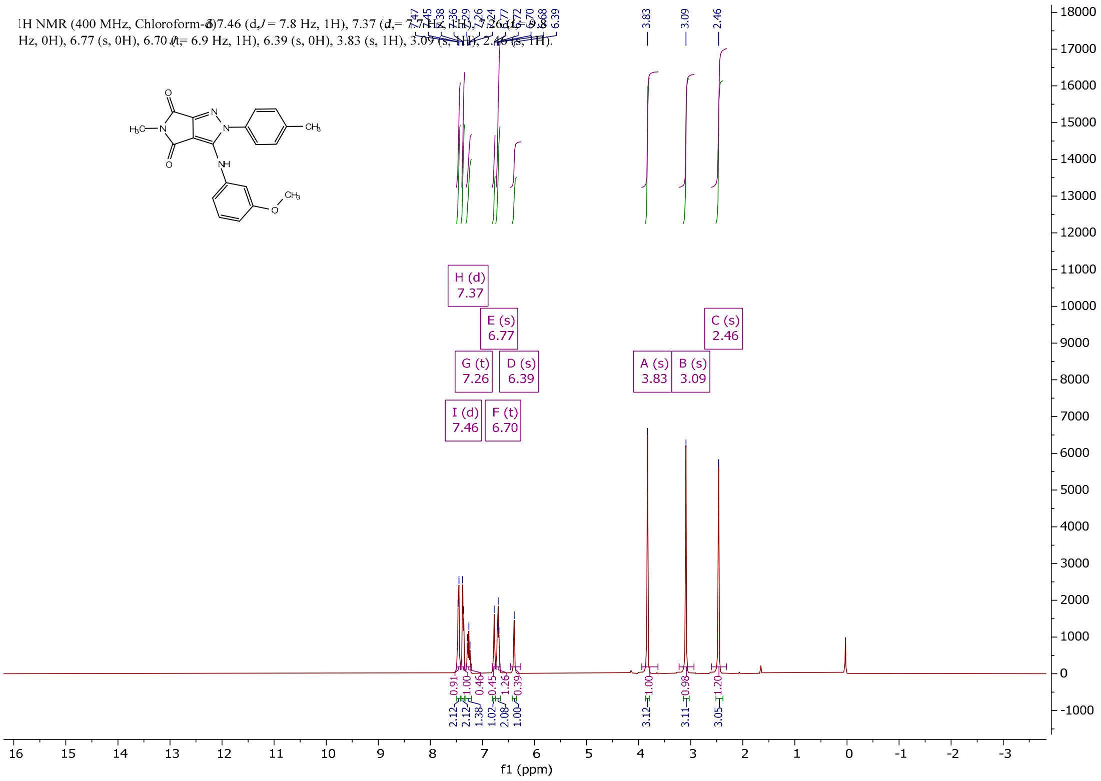

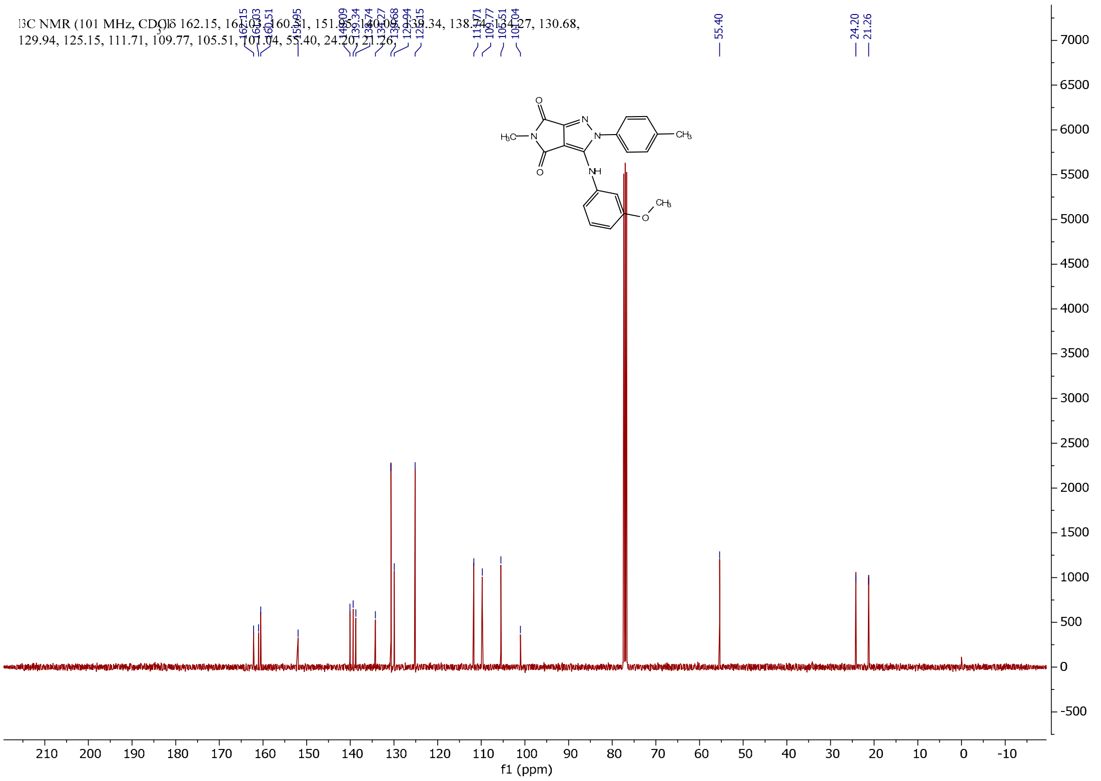

Figure S31: 3-((2-Methoxyphenyl)amino)-5-methyl-2-(p-tolyl)pyrrolo[3,4-c]pyrazole-4,6-(*2H,5H*)-dione (34)

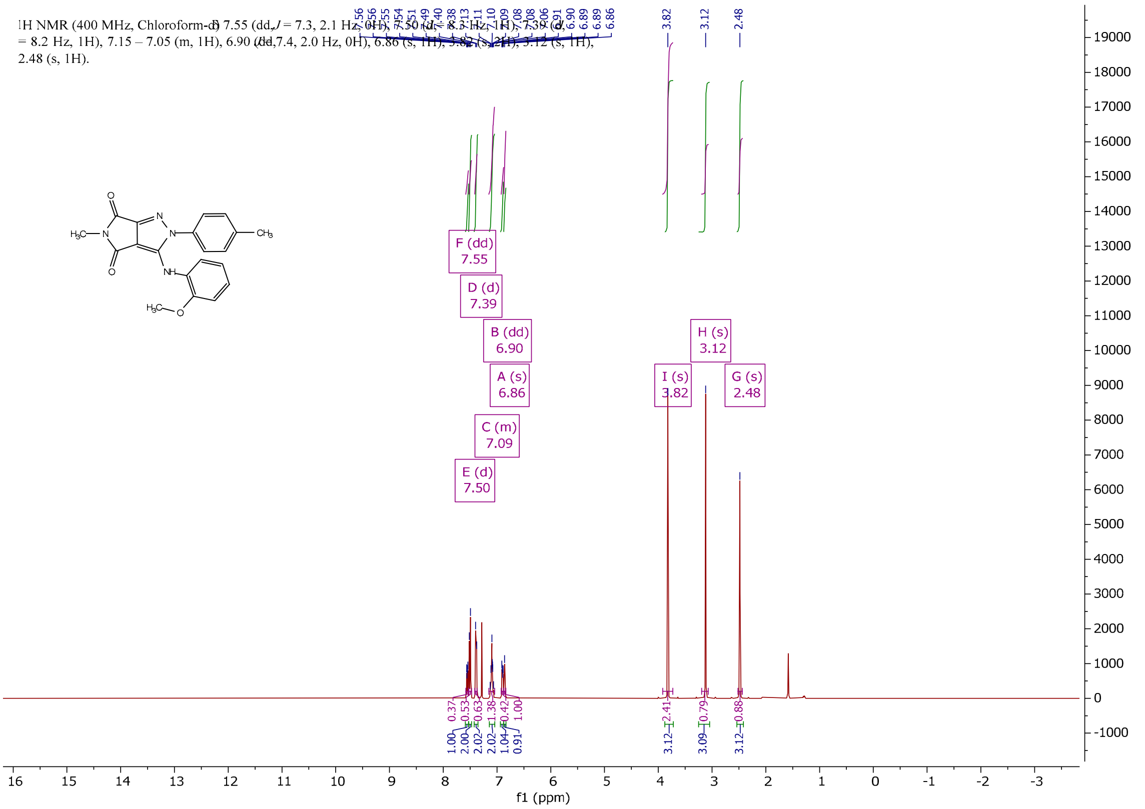

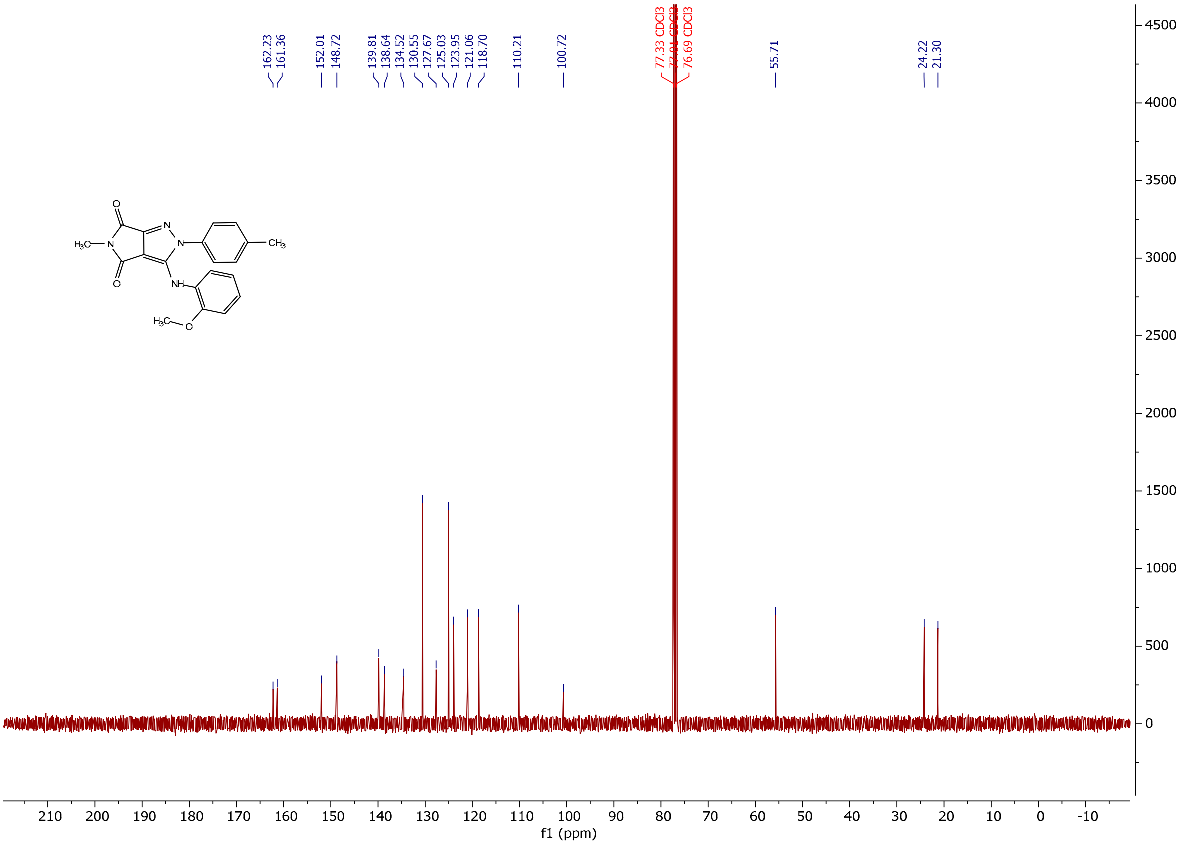

Figure S32: 5-Methyl-2-(p-tolyl)-3-((4-(trifluoromethyl)phenyl)amino)pyrrolo[3,4-c]pyrazole-4,6-(*2H,5H*)-dione (35)

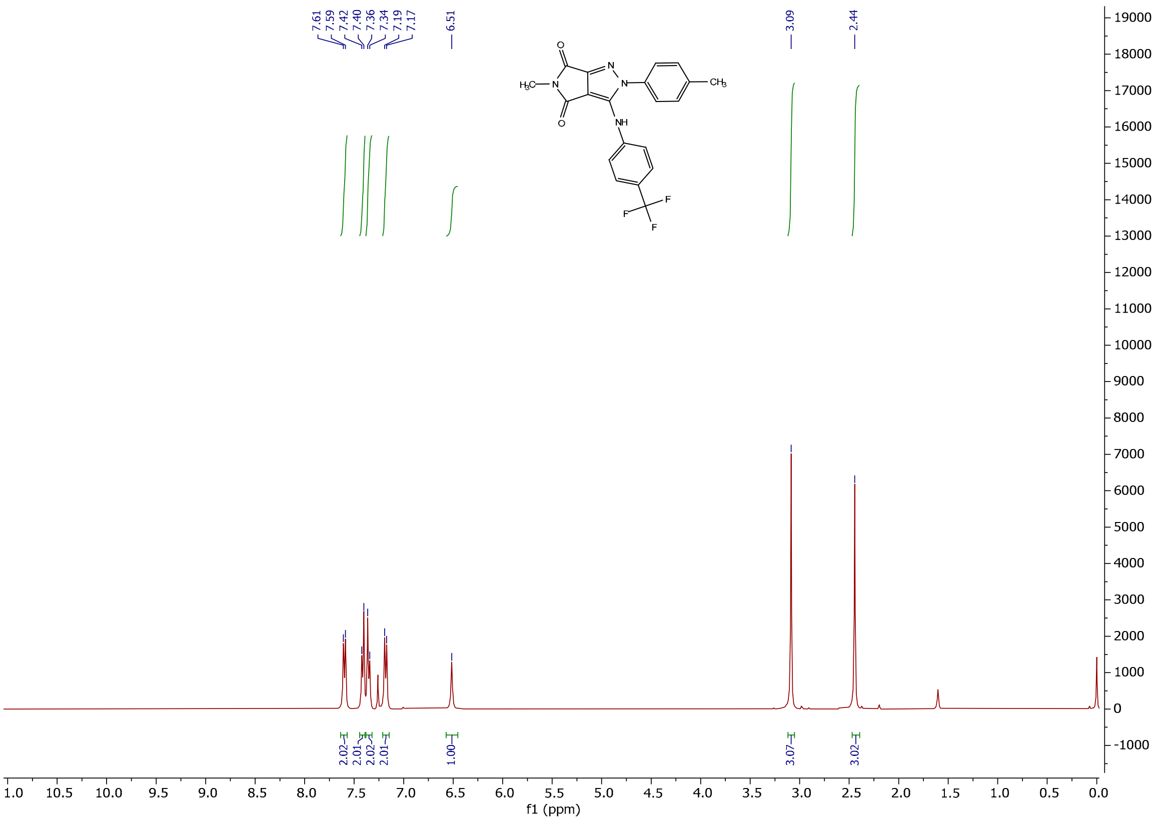

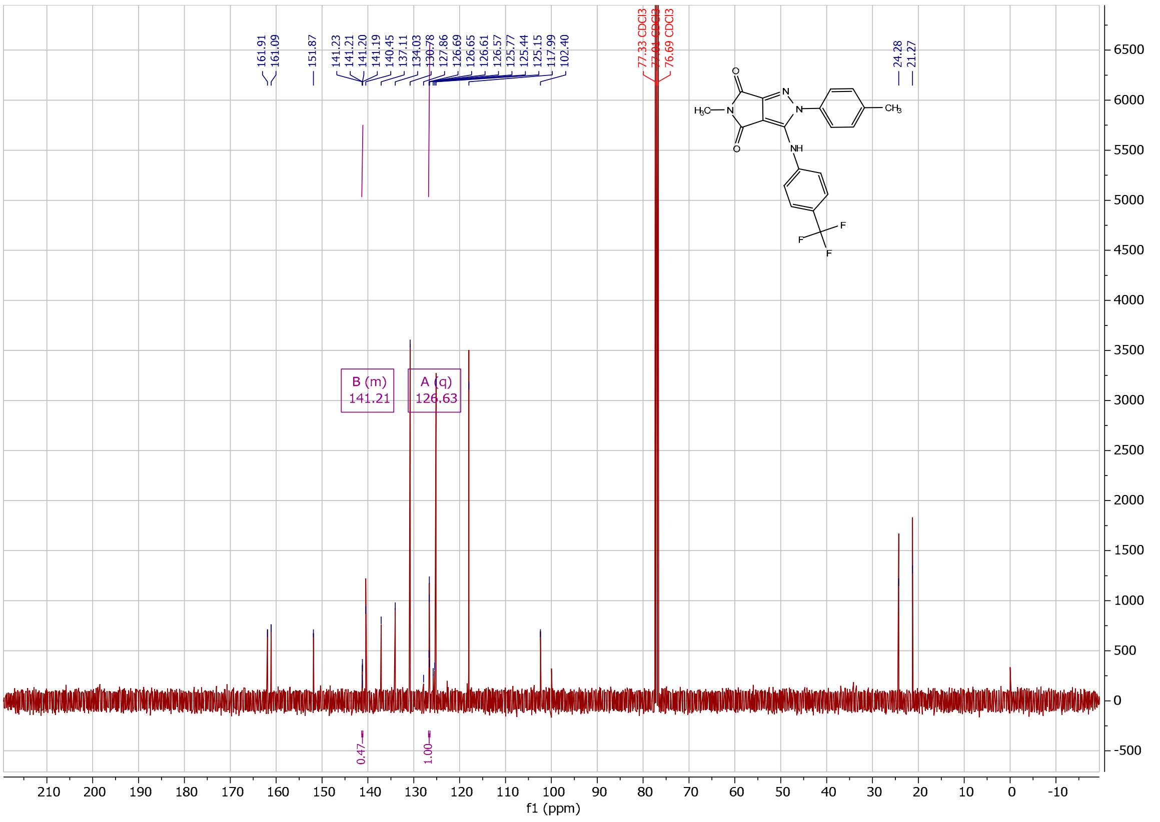

Figure S33: 5-(4-Methoxybenzyl)-3-(anilino)-2-(p-tolyl)pyrrolo[3,4-c]pyrazole-4,6-(*2H,5H*)-dione (39)

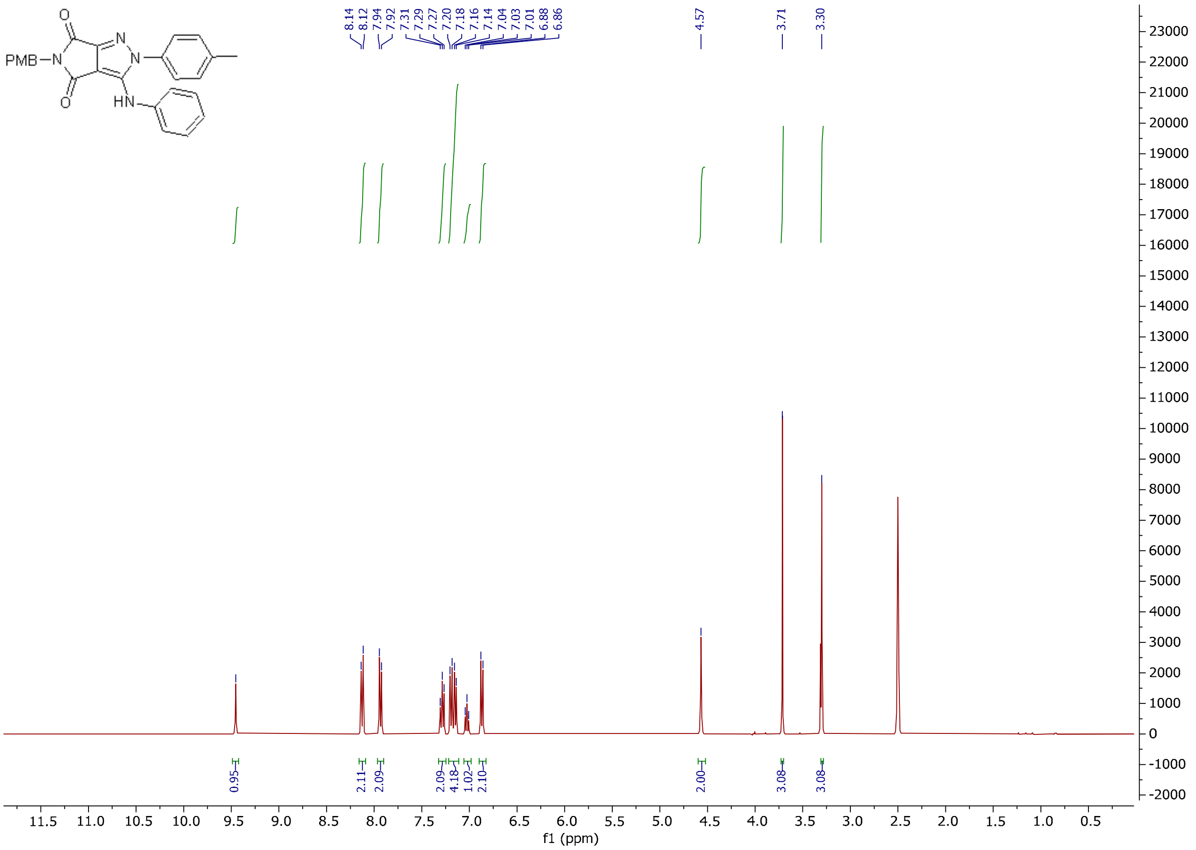

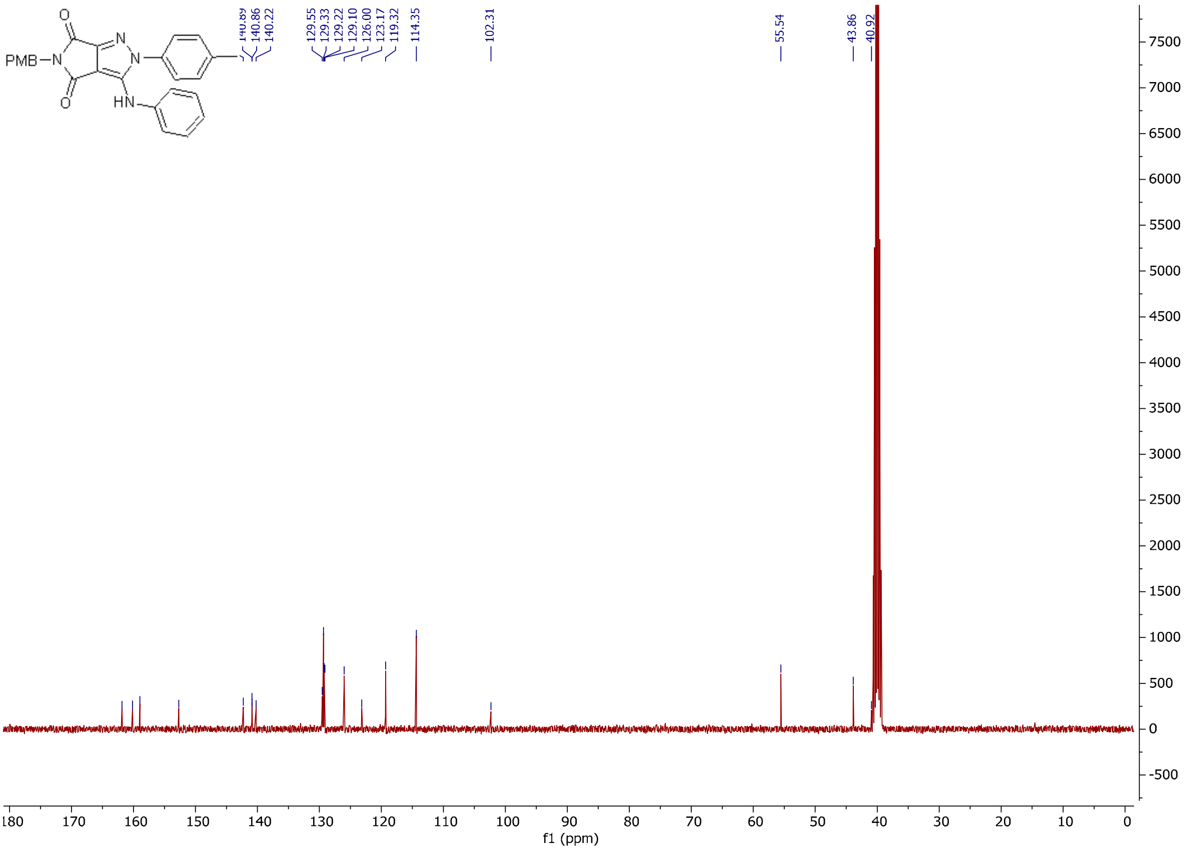

Figure S34: 5-Methyl-2-(4-nitrophenyl)-3-(phenylamino)pyrrolo[3,4-c]pyrazole-4,6-(*2H,5H*)-dione (40)

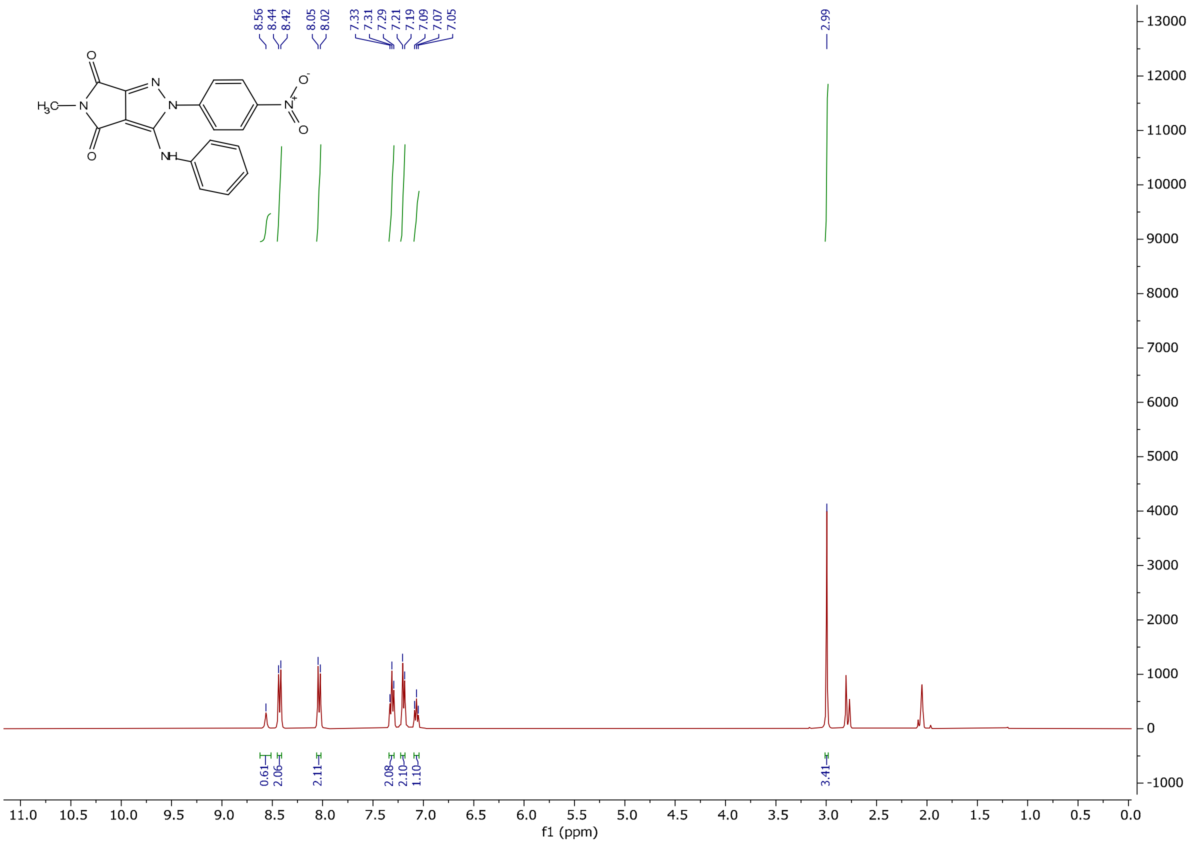

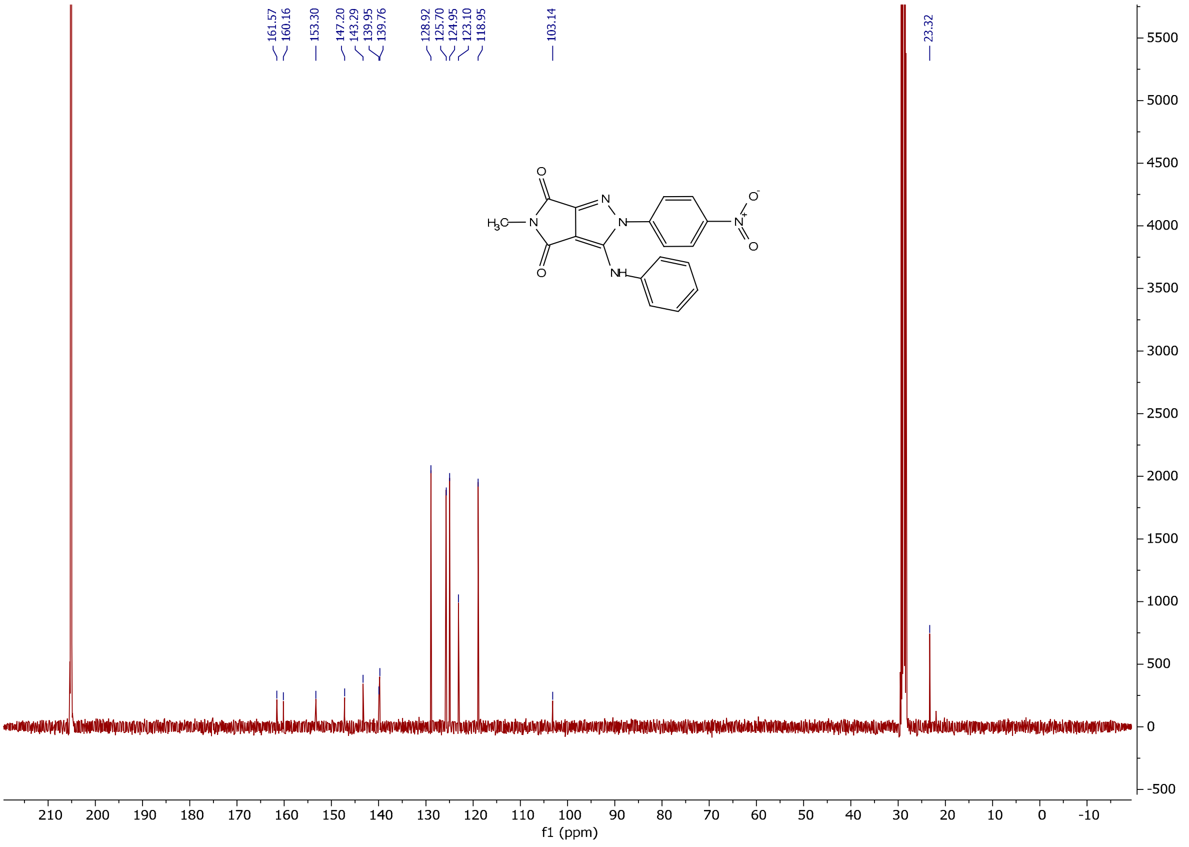

Supplement: Supplementary file 1 [file molecules-28-05811-s001.zip › molecules-2519445-supplementary.pdf]
